# Impact of a single water molecule on the atmospheric oxidation of thiophene by hydroperoxyl radical

**DOI:** 10.1038/s41598-022-22831-8

**Published:** 2022-11-08

**Authors:** Hamed Douroudgari, Maryam Seyed Sharifi, Morteza Vahedpour

**Affiliations:** Department of Chemistry, University of Zanjan, PO Box 38791-45371, Zanjan, Iran

**Keywords:** Computational chemistry, Environmental chemistry, Environmental impact

## Abstract

Water as an important assistant can alter the reactivity of atmospheric species. This project is designed to investigate the impact of a single water molecule on the atmospheric reactions of aromatic compounds that have not been attended to comprehensively. In the first part, the atmospheric oxidation mechanisms of thiophene initiated by hydroperoxyl radical through a multiwell-multichannel potential energy surface were studied to have useful information about the chemistry of the considered reaction. It was verified that for the thiophene plus HO_2_ reaction, the addition mechanism is dominant the same as other aromatic compounds. Due to the importance of the subject and the presence of water molecules in the atmosphere with a high concentration that we know as relative humidity, and also the lack of insight into the influence of water on the reactions of aromatic compounds with active atmospheric species, herein, the effect of a single water molecule on the addition pathways of the title reaction is evaluated. In another word, this research explores how water can change the occurrence of reactions of aromatic compounds in the atmosphere. For this, the presence of one water molecule is simulated by higher-level calculations (BD(T) method) through the main interactions with the stationary points of the most probable pathways. The results show that the mechanism of the reaction with water is more complicated than the bare reaction due to the formation of the ring-like structures. Also, water molecule decreases the relative energies of all addition pathways. Moreover, atoms in molecule theory (AIM) along with the kinetic study by the transition state (TST) and the Rice–Ramsperger–Kassel–Marcus (RRKM) theories demonstrate that the overall interactions of a path determine how the rate of that path changes. In this regard, our results establish that the interactions of water with HO_2_ (thiophene) in the initial complex 1WHA (1WTA or 1WTB) are stronger (weaker) than the sum of its interactions in transition states. Also, for the water-assisted pathways, the ratio of the partition function of the transition state to the partition functions of the reactants is similar to the respective bare reaction. Therefore, the reaction rates of the bare pathways are more than the water-assisted paths that include the 1WHA complex and are less than the paths that involve the 1WTA and 1WTB complexes.

## Introduction

As is well known, the most abundant atmospheric species are N_2_, O_2_, and H_2_O. The concentration of water in the lower troposphere is high (around 10^18^ molecules cm^−3^)^1^. Also, it is proved that water can alter the rate of some atmospheric reactions due to its unique properties, hydrogen bonding. In the gas phase reactions, a water molecule due to the partial charges of O and H atoms plays a dual role to form hydrogen bonds. In some situations, it acts as a hydrogen bond donor or hydrogen bond acceptor and in others, both roles are seen simultaneously, especially in forming ring-like structures^2^. This property causes the atmospheric particles to have a complex structure with water. Thus, it facilitates the elimination of heterogeneous atmospheric compounds and changes their chemical and photochemical properties. Furthermore, these complexes lead to the formation of water droplets and secondary aerosols. Therefore, water can influence the chemistry of the atmosphere^3–7^.

In the gas phase, Hamilton and Lii reported the first experimental evidence of the water (in vapor form) impact on the self-reaction of HO_2_. Their results showed that the formation of the HO_2_…H_2_O and HO_2_…NH_3_ complexes lead to an increase in the reactivity of HO_2_ compared to the naked state^8^. Aloisio and Francisco through a theoretical study predicted that up to 30% of HO_2_ is complex in the presence of water. They concluded that ignoring this percent makes large errors in atmospheric models^9^. The HO_2_…H_2_O complex has also been involved in the heterogeneous uptake of hydroperoxyl radical by water^10^. In the last decade, the effects of water vapor on the main reaction pathways of methane, formic acid, acetaldehyde, acetone, HOCl, glyoxal, dimethyl sulfide (DMS), propionaldehyde, sulfuric acid nitric acid, and HCl with hydroxyl radicals have been investigated by several researchers^11–20^. In the dimethyl sulfide, dimethyl sulfoxide, and dimethyl sulfone reactions with OH, the presence of the water molecule reduces the barrier height of the H-abstraction channel. Also, the results indicated that the transition states are more stabilized in the DMSO + OH…H_2_O reaction than the DMS + OH…H_2_O reaction compared to the respective naked reactions^17^. However, in some simulated atmospheric reactions, an increase in the calculated rate constant was obtained for the water-assisted reactions compared to corresponding bare reactions. For example, the rate of H_2_SO_4_ + OH reaction in the presence of water is higher than the naked reaction (about 10^3^times)^19^. In contrast, water not only cannot accelerate the rate of the NCO plus HCHO reaction, but also it has a negative influence on that reaction^21^. The same trend is also reported for the HNO_3_ + OH reaction rate. The main reason is a rearrangement in the geometry of the hydrogen-bonded pre-reactive complex, which causes the kinetics of the reaction to be more complicated, and ultimately entire reaction process is slow^12^.

The simple and volatile organic compounds containing a sulfur atom in the field of heterocyclic chemistry, thiophene, and its derivatives, are emitted into the atmosphere by numerous sources such as the combustion of fossil fuels, burning plants, and biomasses^22,23^. The detection of sulfur compounds like thiophenes over seaweed fields and oceans was reported previously. Also, oxidation of these compounds occurs (with a high likelihood) near the surface of oceans in the marine boundary layer. It should be noted that the water concentration in this region is too high. So, it may form hydrated complexes with those compounds^17,24^.

It is worth mentioning that the removal of aromatic compounds is the main atmospheric issue. So, the oxidation of these chemicals plays a pivotal role in both atmospheric chemistry and the combustion process. In this regard, the oxidations of thiophene with active species have been studied widely^25–27^.

As we know, atmospheric compounds react preferably with hydroxyl radicals. So, the gas phase oxidation of thiophene is widely considered by hydroxyl radical experimentally and theoretically^27–35^. Some research showed that other active radical species such as ^3^O^36–39^, ^3^O_2_^26,35,40^, ^3^O_3_^29,35^, NO_3_^41–43^, and Cl^44,45^ contribute to removing the atmospheric thiophene. Computationally, in the thiophene plus ^3^O, ^3^O_2_, and NO_3_ reactions, the most suitable paths are energetically related to the addition/elimination reactions because of having a small barrier than the H abstraction reactions^26,39,43^. So, theoretical results proved that the main reaction channel is for the addition/elimination pathways. It is better to say that hydroperoxyl radical has high reactivity in the gas phase after hydroxyl radical. So, HO_2_ plays an important role in the degradation of atmospheric pollutants which has been considered by others^46–48^. Also, the atmospheric hydroperoxyl radical play a pivotal role in hydrocarbon combustions^49^. Therefore, HO_2_ is a key atmospheric species.

In addition, in atmospheric chemistry, the well-known oxidants are HO_X_ family (OH and HO_2_ radicals). They play a crucial role in the degradation of atmospheric pollutants due to stratospheric ozone. Also, these species are important in tropospheric oxidation reactions^50,51^. Furthermore, it is demonstrated that oxidation reactions with HO_2_ are the main way to lose many important molecules and radicals in the troposphere^52,53^.

On one hand, according to the experimental and theoretical results, up to 30% of HO_2_ forms the hydrated complexes with water, and neglecting it causes significant errors in the atmospheric models^8,9^. On the other hand, as mentioned above, a large portion of sulfur-containing aromatic compounds are in the hydrated form. So, water will play a significant role in the reactions of thiophene-based compounds when the oxidant is HO_2_.

In this study, we will present an example to show how treatment of aromatic compound reactions changes in a wet gas phase media (a fact that goes beyond this particular reaction). A straightforward approach to accomplish this aim is using simple atmospheric models. But, as we know, the reactions of aromatic compounds are complex. Thus, we select the thiophene reaction with hydroperoxyl radical that has been studied by the same authors in the dry environment^54^. We showed that this reaction begins with the addition of HO_2_ to thiophene and proceeds through different channels. Here, we consider the impact of water on addition reactions and epoxy formation due to OH elimination only. First, we will construct the PES of the title reaction and investigate the influence of water on the relative energies of all stationary points. The geometries of species involved in the PES will be analyzed to find the main reason for the stability of water-assisted reactions. It will be shown that strong interactions due to water presence play an important role in the formation of ring-like structures in the course of the simulated reaction. Also, NBO analysis will be used for elucidating water's role in the stability of stationary points. We will use the AIM theory for clarifying the nature and strength of the interactions in ring-like structures. Second, the kinetic of the designed channels will be investigated to elucidate how water position impacts the rate of reaction. Finally, the results of wet and naked reactions will be compared for judging the water's role in the reactions of aromatic compounds. This study will aid the understanding and rationalization of similar processes that may occur in the gas-phase oxidation of other heterocyclic and aromatic compounds in the atmosphere.

## Theoretical methods

We have previously shown that, for the thiophene plus HO_2_ reaction, the B3LYP^55^ method has good accuracy for simulation results^54^. Consequently, the potential energy surfaces for the addition of HO_2_ to thiophene in the naked and water-assisted reaction are examined by the DFT-B3LYP method in conjunction with the Pople type basis set 6–31 + G(d,p). However, full optimization of the probable structure of all stationary points with and without the presence of water accompanied by vibrational frequencies is carried out at the DFT-B3LYP/6–31 + G(d,p) level of theory. The obtained frequencies along with the intrinsic reaction coordinate^56^ (IRC) calculations are employed to verify the validity of the computed saddle pints structures and their connectivity at the mentioned level of theory. The optimized structures of all saddle points contain just an imaginary frequency that shows an alteration along the reaction coordinate. Also, each transition state is connected to the respective minimum structures, such as pre-and post-reactive complexes by the corresponding IRC^56^ path. Furthermore, a high level of quantum chemical calculations by means of the CBS-QB3^57^ and CCSD(T)^58^ methods are utilized to compute the main energetic parameters with high accuracy. But, some of the calculated T1 diagnostic values^59,60^ from the CCSD(T) method are higher than the threshold value (0.045 for open-shell species), which shows that a higher level calculation is necessary to reach accurate energetic parameters. Thus, we use the Brueckner Doubles calculation with triples contribution, the BD(T) method^61,62^. In addition, for each stationary point, the correction for internal energy, enthalpy, and free energy obtained at the B3LYP/6–31 + G(d,p) level accompanied by the calculated energies at the CCSD(T) and BD(T) methods are mixed to produce thermodynamic parameters with high precision. Also, a similar manner is used for extracting valid kinetic data of the title reaction in which the partition functions are calculated at the B3LYP/6–31 + G(d,p) level and the energies are computed at the BD(T) method. All energetic parameters and harmonic vibrational frequencies are computed by the Gaussian 09 package^63^. The visualization of all structures is carried out by the Chemcraft program^64^. The nature of the line critical points (LCP)^65^ and the ring critical points (RCP) is analyzed by using the topological analyses of atoms in molecules theory, AIM. The electron density and related parameters are visualized by the AIM2000 package^66^.

Kinetic studies were accomplished for all pathways using the conventional transition state (TST)^67^ and RRKM^68^ theories by using the obtained energies of the highest theoretical level, BD(T))/6-31+G(d,p). The Gpop (for bimolecular reactions) and Ssumes (for unimolecular reactions) programs are applied as standard software for the calculations of the rate constants of the pathways^69,70^.

### Determining reactive centers in thiophene and hydroperoxyl radical by the Fukui function

In DFT, the Fukui functional is a favorite concept for determining a more reactive site on a molecule^71,72^. This function for a compound containing N electron is defined by

1a $$f(r) = \left( {\frac{\partial \rho (r)}{{\partial N}}} \right)_{\nu (r)}$$

where $\rho (r)$ is the electron density.$N$ is the number of atoms in the studying molecule. $\upsilon (r)$ is a constant external potential. This function is not evaluated directly because of a limitation associated with discontinuity^73,74^. Thus, the modified equations based on the involved mechanism in the investigating reaction are used for calculating the Fukui function^75^.

- For the nucleophilic attack, the following equation is used
  1b $$f_{X}^{ + } = [P_{X} (N + 1) - P_{X} (N)]$$
  where $P_{X} (N + 1)$ and $P_{X} (N)$ are the population of atom X in molecule Y that denote the electron states with − and 0 signs, respectively, obtained from the geometry of neutral species.
- For the electrophilic attack, it can be written
  1c $$f_{X}^{ - } = [P{}_{X}(N) - P_{X} (N - 1)]$$
  where $P(N - 1)$ is for the state with + signs.
- For the radical attack, we have
  1d $$f_{X}^{0} = \frac{1}{2}[P_{X} (N + 1) - P_{X} (N - 1)]$$

The population of an atom is obtained by subtracting the atomic number ($A$) of the target atom from its atomic charge ($Z$)

1e $$P_{X} = A - Z$$

We apply the Hirschfeld charges in the calculation of the population of atom X. It is worth mentioning that the charge transfer mechanism is studied by using these formulas. Therefore, the Fukui function is a useful parameter to identify the reactive sites on a molecule. Tables S14–S21 contain the Fukui analysis results for all stationary points of the title reaction.

## Results and discussion

### Fraction of possible complexes formed between HO_2_ and H_2_O, and thiophene and H_2_O molecules

In this section, we argue about the complexes that may take part in the title reaction. First, all possible complexes are found by closing water molecules from different sides to the hydroperoxyl radical and thiophene molecules separately. Then, using the kinetic theory of gases, the fraction percent of those complexes is calculated based on their stability. In this regard, the Boltzmann distribution is used as follows^76^:

2a $$N_{i} = c\exp \left( {\frac{{\Delta E_{i} }}{{k_{B} T}}} \right)$$

where $N_{i}$ is the number of molecules with energy $\Delta E_{i}$ and $c$ is constant. $k_{B}$ is the Boltzmann constant. The fraction of molecules which has the energy $\Delta E_{i}$ is

2b $$f_{i} = \frac{{N_{i} }}{N} = \frac{{\exp \left( {\frac{{\Delta E_{i} }}{{k_{B} T}}} \right)}}{{\sum\limits_{i}^{{}} {\exp \left( {\frac{{\Delta E_{i} }}{{k_{B} T}}} \right)} }}$$

where $N$ is the total number of molecules. This equation shows obviously that the most stable complexes will have a higher percentage. This is an important result since it helps us to find more abundance complexes and consider them for reaction design.

**Figure 1:**
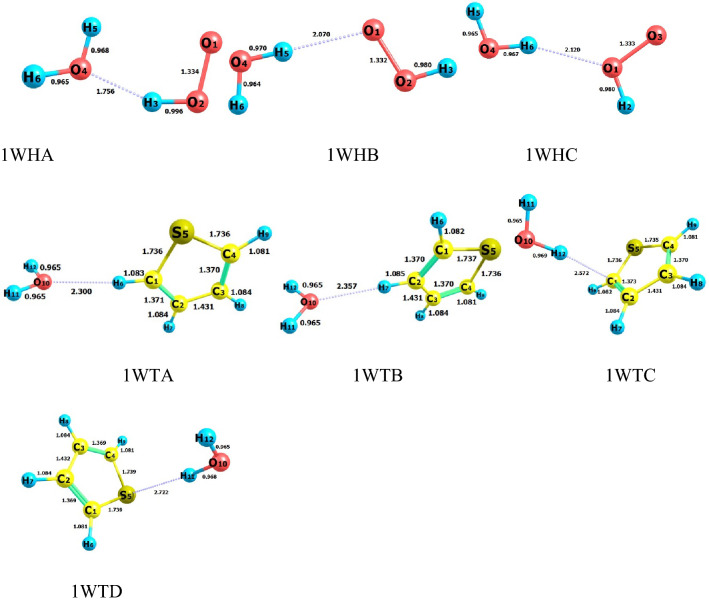
Possible complexes established between hydroperoxyl radical and water molecules, and also thiophene and water molecules (unit of all distances is angstrom).

Figure 1 shows all possible complexes between hydroperoxyl and water molecules. By the possible interactions that may form between HO_2_ and H_2_O molecules, three stable H-bonded complexes are established and named 1WHA, 1WHB, and 1WHC. The most stable complex is 1WHA with a relative energy of − 9.99 kcal mol^−1^ and after that 1WHB (− 3.60 kcal mol^−1^). The origin of more stability for 1WA is the formed hydrogen bond ($\rho (lcp)$ = 0.0392 e bohr^−3^ and $\nabla^{2} \rho (lcp)$ 0.1153 e bohr^−5^) in which water is a hydrogen bond acceptor. In the 1WHB and 1WHC complexes, the water molecule is a hydrogen bond donor with the electronic charge density of $\rho_{1WHB} (lcp)$ = 0.0192 and $\rho_{1WHC} (lcp)$ = 0.0196 e bohr^−3^, and its Laplacian $\nabla^{2} \rho_{1WHB} (lcp)$ = 0.0527 and $\nabla^{2} \rho_{1WHC} (lcp)$ = 0.0492 e bohr^−5^. The percent of the 1WHA fraction is almost 100% at 300 K. This result proposed that all HO_2_ molecules at room temperature are complexed with water in the 1WHA form. Also, Table 1 shows that this complex has a dominant fraction over the 300–3000 K temperature range.

**Table 1:** The relative energy (kcal mol^−1^) and the percentage of fractions for the probable complexes formed between HO_2_ and H_2_O, and thiophene and H_2_O computed at the BD(T) method.

Complex	Energy	%Fraction (300.00 K)	%Fraction (1000.00 K)	%Fraction (2000.00 K)	%Fraction (3000.00 K)
HO_2_ + HO_2_	0.00	–	–	–	–
1WHA	− 9.99	100.00	93.38	72.69	60.40
1WHB	− 3.60	0.00	3.74	14.55	20.67
1WHC	− 3.08	0.00	2.88	12.76	18.93
Thio + HO_2_	0.00	–	–	–	–
1WTA	− 3.27	32.11	29.95	28.30	25.94
1WTB	− 2.72	12.89	17.32	20.10	23.67
1WTC	− 3.42	41.51	34.93	31.16	26.61
1WTD	− 2.75	13.50	17.80	20.45	23.78

As abovementioned, thiophene can be hydrated by water molecules. Thus, the probable complexes between thiophene and water are explored. The sketch of the complexes is depicted in Fig. 1. By closing a water molecule to thiophene, four different complexes are yielded named 1WTA, 1WTB, 1WTC, and 1WTD. These complexes have also near relative energies. The obtained relative energies forecast that the 1WHA and 1WHB (HO_2_…H_2_O) complexes are more stable than the thio… H_2_O complexes under the same conditions. This is related to the nature of interactions among the interacting molecules. In the 1WTA and 1WTB complexes, there is a van der Waals interaction between nonbonding electrons of water with α hydrogen ($\rho (lcp)$ = 0.0122 e bohr^−3^ and $\nabla^{2} \rho (lcp)$ = 0.0395 e bohr^−5^) and β hydrogen ($\rho (lcp)$ = 0.0109 e bohr^−3^ and $\nabla^{2} \rho (lcp)$ = 0.0361 e bohr^−5^) of thiophene. For the 1WTC complex, the interaction between the hydrogen of water with π electrons of thiophene is also van der Waals type ($\rho (lcp)$ = 0.0098 e bohr^−3^ and $\nabla^{2} \rho (lcp)$ = 0.0271 e bohr^−5^). Finally, there is a van der Waals interaction between the hydrogen of water and nonbonding electrons of the sulfur atom in the 1WTD complex ($\rho (lcp)$ = 0.0089 e bohr^−3^ and ∇^2^ρ(r _rcp_) = 0.0267 e bohr^−5^). The computed fractions at 300.00 K (and relative energy) for 1WTA, 1WTB, 1WTC, and 1WTD complexes are 32.11% (− 3.27 kcal mol^−1^), 12.89% (− 2.72 kcal mol^−1^), 41.51% (− 3.42 kcal mol^−1^), and 13.50% (− 2.75 kcal mol^−1^), respectively. It should be noted that the 1WTC complex during the reaction when HO_2_ is close to interacting with it will convert to the 1WHX complex (X = A, B, and C) due to the orientation of the complexed water molecule. Thus, we skip its assistance for reaction.

#### The gas-phase reaction of C_4_H_4_S + HO_2_ without and with water

**Figure 2:**
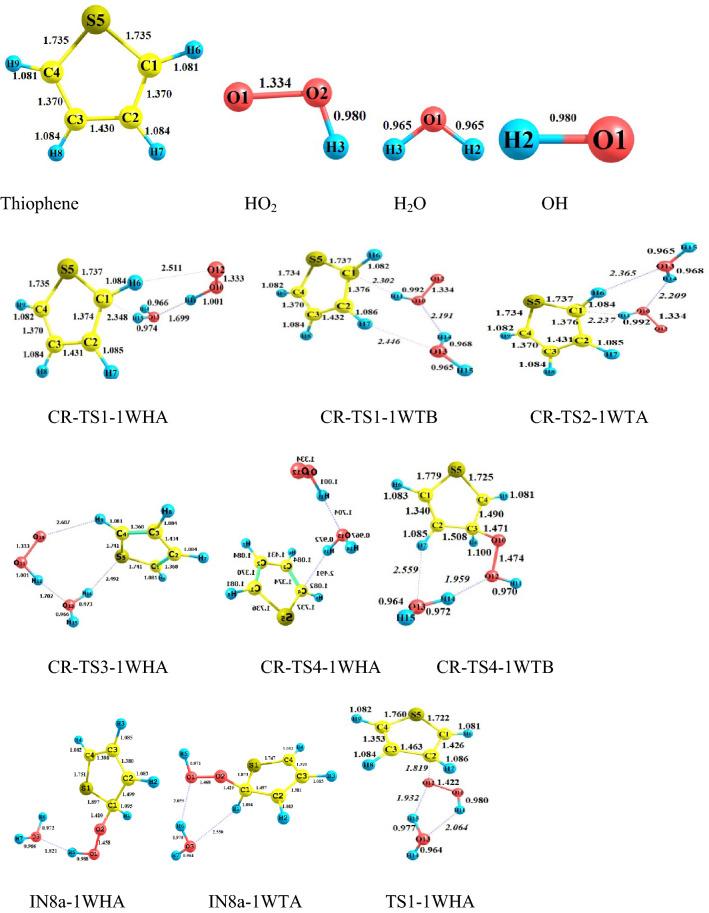

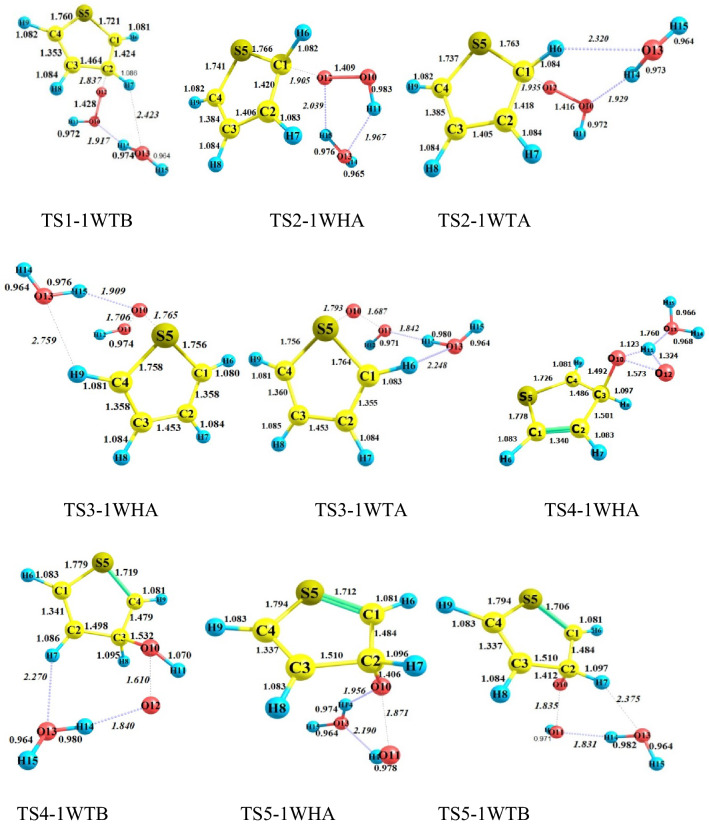

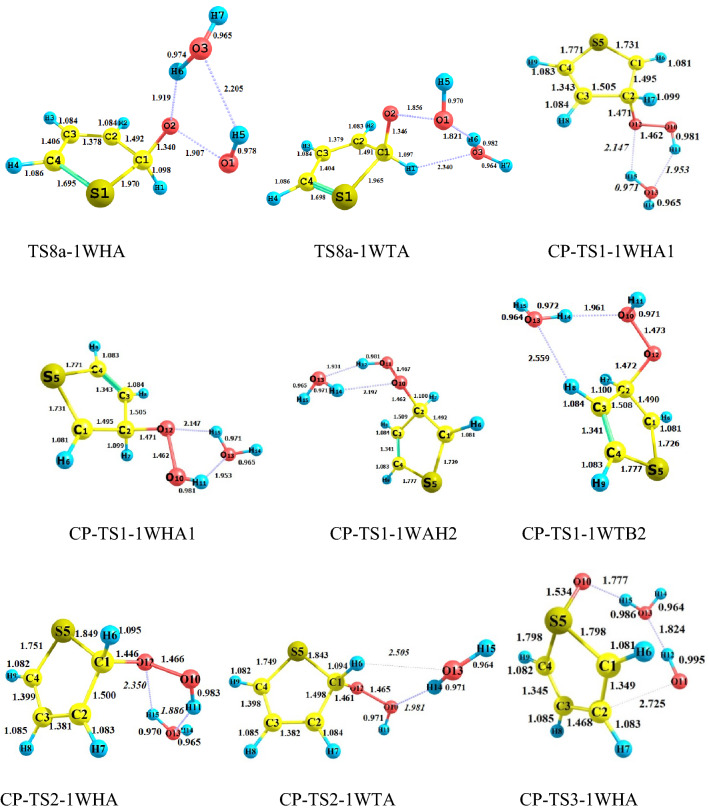

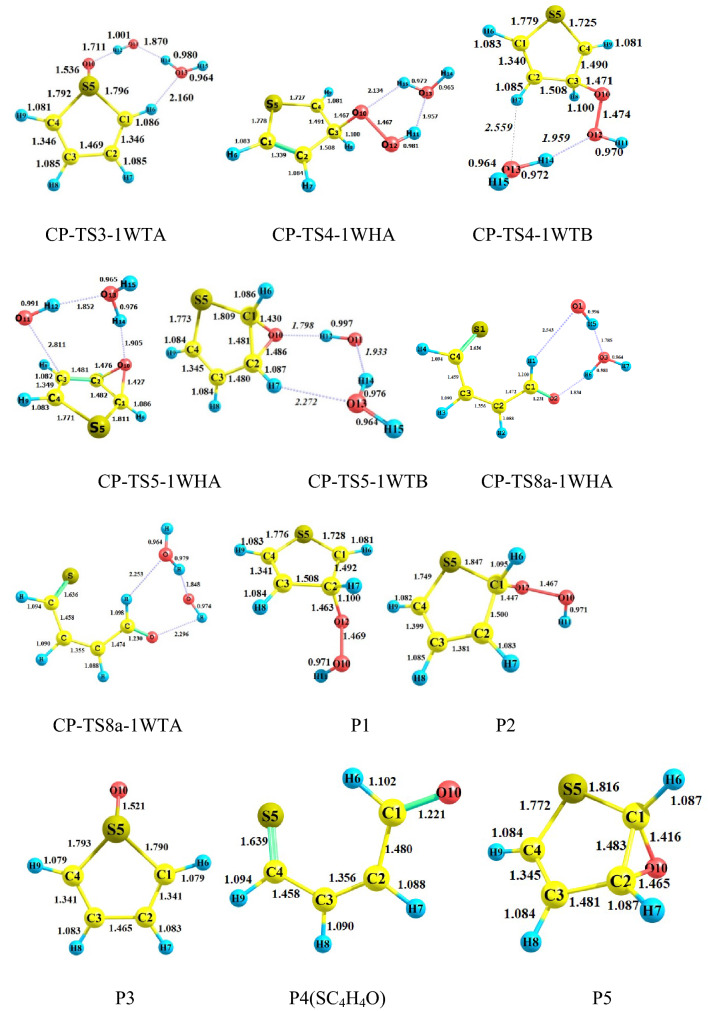
Bond lengths of the reactants and products, and all fragments of the pre-reactive, intermediates, transition states, and post-reactive complexes optimized at the B3LYP/6–31 + G(d,p) level (unit of all distances is angstrom).

**Figure 3:**
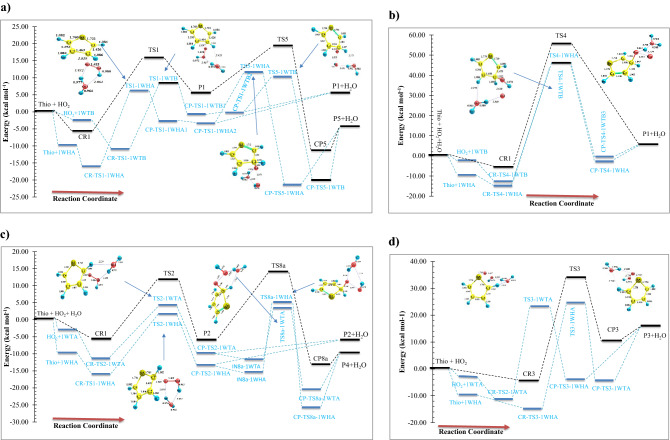
The energy profiles (at the BD(T) method) of the addition pathways in the thiophene + HO_2_ reaction (the bare paths are shown by black dash lines, and the water-assisted paths are drawn by blue dash lines). (**a**) The terminal oxygen addition to β-C (first step) and the epoxy formation (second step). (**b**) The middle oxygen addition to β-C. (**c**) The terminal oxygen addition to α-C (first step) and ring-opening (second step). (**d)** The terminal oxygen addition to the sulfur atom.

**Figure 4:**
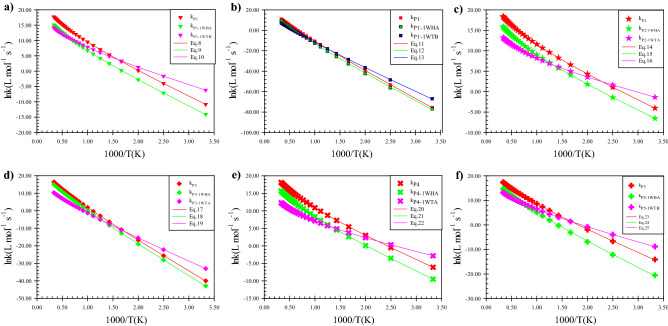
Graphs of all rate constants and fitted non-Arrhenius expressions for the addition mechanism in the thiophene + HO_2_, thiophene + 1WHA, 1WTA + HO_2_, and 1WTB + HO_2_ reactions obtained at the BD(T)/6–31 + G(d,p) level of theory. (**a**) The terminal oxygen addition to β-C. (**b**) The middle oxygen addition to β-C. (**c**) The terminal oxygen addition to α-C. (**d**) The terminal oxygen addition to sulfur atom. (**e**) Ring-opening pathway. (**f**) Epoxy formation pathway.

All possible complexes between reactant species and water are displayed in Fig. 1. The main parameters of the optimized geometries for all reaction components computed by the B3LYP method are shown in Fig. 2. The potential energy surfaces (PESs) for the title reaction in the presence and absence of water are drawn in Fig. 3. the rate constants of all paths are depicted in Fig. 4 and are listed in Tables S9 and S10. The energetics of each stationary point is compared by considering the original reactants as the base of comparison and are listed in Tables 2 and 3. Also, for all interacted molecules, the extracted T1 diagnostic values from the CCSD(T) calculations are listed in Tables 2 and 3. The useful thermodynamic parameters, such as inner energies, enthalpies, Gibbs free energies, and entropies computed in the standard conditions are collected in Table 4 and Tables S3 and S4 (Supporting information). From a computational point of view, the doublet state (as an open-shell system) may have the spin contamination problem. So, it must be checked during calculations. Therefore, the expectation values are checked after calculations of all complexes, transition states, and other radical species. The results show that the spin contamination values [⟨S_2_⟩_obs_ ≈0.75] never exceed 0.066 (see Table S1 of Supporting Information). Thus, the obtained energetics are acceptable. The optimized stationary points of the C_4_H_4_S + HO_2_…H_2_O reaction have ring-like structures due to intermolecular interactions, then the AIM analysis is utilized to extract beneficial information about the line and ring critical points. The results of AIM are reported in Table S2.

**Table 2:** Relative energies (in kcal mol^−1^) of the reactants, products, intermediates, and transition states involved in the bare reaction at different levels of theory.

Stationary points	This work					Previous study			
	B3LYP^a^	CBS-QB3	CCSD (T)^b^	BD(T)^c^	T1^d^	B3LYP ^e^	CCSD (T)^f^	BD(T) ^g^	T1^h^
C_4_H_4_S + HO_2_	0.00	0.00	0.00	0.00	–	0.00	0.00	0.00	–
CR1	− 3.41	− 4.81	− 5.61	− 5.92	0.033	0.68	− 6.39	− 6.64	0.021
CR3	− 2.74	− 4.33	− 4.74	− 4.76	0.022	–	–	–	–
TS1	17.06	14.70	16.35	15.60	0.037	21.72	17.59	14.69	0.035
TS2	11.97	11.11	12.37	11.52	0.037	16.51	14.49	10.61	0.035
TS3	33.26	29.00	35.32	33.64	0.053	38.41	35.24	32.80	0.038
TS4	57.76	54.63	55.02	55.13	0.037	63.36	56.39	54.22	0.034
TS5	21.70	21.11	20.44	19.12	0.047	25.81	25.96	18.36	0.044
TS8a	10.42	17.15	16.45	13.80	0.052	14.16	22.34	12.42	0.053
CP3	13.95	− 0.53	10.09	10.11	0.019	18.62	5.49	9.33	0.017
CP5	− 2.47	− 6.42	− 11.47	− 11.49	0.019	1.69	− 8.06	− 12.57	0.016
CP8a	− 10.49	− 8.73	− 10.72	− 13.39	0.052	− 7.68	− 7.15	− 14.16	0.020
P1	10.74	6.38	5.52	5.29	0.025	15.89	5.76	4.47	0.023
P2	− 1.90	− 2.80	− 5.81	− 6.20	0.029	5.95	− 2.58	− 4.98	0.025
P3(C_4_H_4_SO + OH)	18.72	6.19	15.80	15.64	–	19.26	10.24	15.02	–
P4(SC_4_H_4_O + OH)	− 7.60	− 4.43	− 9.92	− 9.93	–	− 9.34	− 4.56	− 10.55	–
P5(SCHOCHC_2_H_2_ + OH)	1.84	− 2.34	− 4.34	− 4.51	–	1.64	− 1.77	− 5.13	–

^a^B3LYP method in conjunction with 6–31 + G(d,p) basis set.

^b^CCSD(T) single point on the optimized structure of the B3LYP/6–31 + g(d,p) level.

^c^BD(T)/6–31 + G(d,p)//B3LYP/6–31 + G(d,p) level.

^d^T1 diagnostic values at the CCSD(T)/6–31 + G(d,p) level.

^e^B3LYP method in conjunction with 6–311 + g(d,p) basis set.

^f^CCSD(T)/CC-PV(T + d)Z//B3LYP/6–311 + G(d,p) level.

^g^BD(T)/6–31 + G(d,p)//B3LYP/6–311 + G(d,p) level.

^h^T1 diagnostic values at the CCSD(T)/CC-PV(T + d)Z level.

**Table 3:** Relative energies (in kcal mol^−1^) of the reactants, products, intermediates, and transition states involved in the water-assisted reactions at three different levels of theory.

Stationary points	Water-assisted reaction				
	B3LYP^a^	CBS-QB3	CCSD (T)^b^	BD(T)^c^	T1^d^
C_4_H_4_S + HO_2_ + H_2_O	0.00	0.00	0.00	0.00	–
Thiophene + 1WHA	− 7.43	− 6.13	− 9.97	− 9.99	0.028
HO_2_ + 1WTA	− 1.43	–	− 3.27	− 3.27	
HO_2_ + 1WTB	− 1.01	–	− 2.73	− 2.72	
CR-TS1-1WHA	− 11.05	− 11.38	− 15.91	− 16.24	0.033
CR-TS1-1WTB	− 5.95	− 10.35	− 10.90	− 11.20	0.034
CR-TS2-1WTA	− 6.29	–	− 11.39	− 11.69	0.030
CR-TS3-1WHA	− 10.36	− 11.71	− 14.78	− 15.26	0.034
CR-TS4-1WHA	− 10.16	− 12.59	− 15.34	− 15.36	0.021
CR-TS4-1WTB	− 7.94	− 8.86	− 12.79	− 13.08	0.030
TS1-1WHA	11.07	8.80	6.60	5.95	0.033
TS1-1WTB	12.85	10.86	8.87	8.16	0.034
TS2-1WHA	5.57	6.97	2.23	1.40	0.034
TS2-1WTA	7.59	4.21	4.81	3.99	0.034
TS3-1WHA	28.18	23.72	24.90	24.28	0.034
TS3-1WTA	26.28	25.19	24.46	22.92	0.047
TS4-1WHA	51.42	48.28	46.45	46.51	0.027
TS4-1WTB	50.52	48.46	45.79	45.64	0.033
TS5-1WHA	18.19	16.80	12.74	11.36	0.043
TS5-1WTB	15.27	16.03	11.61	10.00	0.046
TS8a-1WHA	6.56	11.63	5.36	4.83	0.045
TS8a-1WTA	3.84	10.43	3.76	3.12	0.050
IN8a-1WHA	− 8.08	− 11.34	− 15.29	− 15.67	0.024
IN8a-1WTA	− 4.71	− 7.54	− 11.58	− 11.97	0.025
CP-TS1-1WHA1	6.03	1.59	− 2.80	− 3.02	0.023
CP-TS1-1WTB1	7.53	3.59	− 0.79	− 1.03	0.025
CP-TS1-1WHA2	5.60	0.84	− 2.80	− 3.64	0.023
CP-TS1-1WTB2	7.72	3.71	− 0.33	− 0.56	0.023
CP-TS2-1WHA	− 5.07	− 8.94	− 13.26	− 13.63	0.016
CP-TS2-1WTA	− 2.26	− 5.90	− 9.71	− 10.08	0.048
CP-TS3-1WHA	4.23	− 7.53	− 4.75	− 4.76	0.023
CP-TS3-1WTA	3.83	− 6.33	− 4.29	− 4.31	0.022
CP-TS4-1WHA	5.64	1.20	− 3.03	− 3.26	0.023
CP-TS4-1WTB	7.26	3.26	− 0.62	− 0.85	0.023
CP-TS5-1WHB	− 9.49	− 13.77	− 21.56	− 21.70	0.021
CP-TS5-1WTB	− 8.70	− 12.92	− 20.24	− 20.32	0.015
CP-TS8a-1WHA	− 18.91	− 14.75	− 23.74	− 26.06	0.046
CP-TS8a-1WTA	− 15.83	− 14.54	− 18.11	− 20.72	0.047
P1 + H_2_O	10.74	6.38	5.52	5.29	–
P2 + H_2_O	− 1.90	− 2.80	− 5.81	− 4.13	–
P3 + H_2_O	18.72	6.19	15.80	15.64	–
P4 + H_2_O	− 7.60	− 4.43	− 9.92	− 9.93	–
P5 + H_2_O	1.84	− 2.34	− 4.34	− 4.51	–

^a^B3LYP method in conjunction with the 6–31 + G(d,p) basis set.

^b^CCSD(T) single point on the optimized structure of the B3LYP/6–31 + G(d,p) level.

^c^BD(T)/6–31 + G(d,p)//B3LYP/6–31 + G(d,p) level.

^d^T1 diagnostic values at the CCSD(T)/6–31 + G(d,p) level.

**Table 4:** The calculated thermodynamic parameters (298.15 K) for all components of the water-assisted reactions using the BD(T) energies and the B3LYP corrections (unit of all numbers is kcal mol^−1^).

Species	ΔE^0^	ΔH^0^	ΔG^0^	TΔS^0^
C_4_H_4_S + HO_2_ + H_2_O	0.00	0.00	0.00	0.00
Thiophene + 1WHA	− 7.53	− 8.12	0.07	− 7.60
HO_2_ + 1WTA	3.19	2.60	10.29	− 7.69
HO_2_ + 1WTB	3.73	3.14	10.54	− 7.41
CR-TS1-1WHA	− 11.91	− 13.09	3.36	− 15.27
CR-TS1-1WTB	− 7.20	− 8.39	6.75	− 13.96
CR-TS2-1WTA	− 7.70	− 8.88	6.45	− 14.14
CR-TS3-1WHA	− 10.94	− 12.13	4.03	− 14.98
CR-TS4-1WHA	− 10.99	− 12.17	3.08	− 14.07
CR-TS4-1WTB	− 8.96	− 10.15	6.24	− 15.21
TS1-1WHA	9.32	8.14	28.47	− 19.14
TS1-1WTB	11.35	10.16	29.61	− 18.26
TS2-1WHA	− 1.49	3.59	23.88	− 25.37
TS2-1WTA	7.18	6.00	25.68	− 18.49
TS3-1WHA	26.60	25.41	44.28	− 17.68
TS3-1WTA	25.30	24.12	43.90	− 18.60
TS4-1WHA	47.13	45.94	65.92	− 18.79
TS4-1WTB	46.57	45.38	65.41	− 18.84
TS5-1WHA	13.96	12.77	32.18	− 18.22
TS5-1WTB	12.66	11.47	31.17	− 18.51
TS8a-1WHA	0.92	6.33	25.70	− 24.78
TS8a-1WTA	5.87	4.68	24.22	− 18.36
IN8a-1WHA	− 11.31	− 12.50	8.32	− 19.64
IN8a-1WTA	− 7.97	− 9.15	9.06	− 17.03
CP-TS1-1WHA1	1.16	− 0.02	19.73	− 18.57
CP-TS1-1WTB1	2.96	1.77	21.02	− 18.06
CP-TS1-1WHA2	0.50	− 0.69	49.73	− 49.23
CP-TS1-1WTB2	3.35	2.17	20.62	− 17.27
CP-TS2-1WHA	− 9.39	− 10.57	9.27	− 18.65
CP-TS2-1WTA	− 6.06	− 7.24	11.96	− 18.02
CP-TS3-1WHA	− 1.70	− 2.88	16.15	− 17.85
CP-TS3-1WTA	− 1.59	− 2.78	15.71	− 17.30
CP-TS4-1WHA	0.91	− 0.27	19.53	− 18.62
CP-TS4-1WTB	3.08	1.90	20.83	− 17.75
CP-TS5-1WHB	− 18.00	− 19.19	0.53	− 18.53
CP-TS5-1WTB	− 17.01	− 18.19	0.69	− 17.70
CP-TS8a-1WHA	− 22.66	− 23.85	− 7.52	− 15.15
CP-TS8a-1WTA	− 17.33	− 18.51	− 2.34	− 14.98
P1 + H_2_O	7.08	6.49	17.47	− 10.39
P2 + H_2_O	− 2.17	− 2.77	8.53	− 10.71
P3 + H_2_O	14.35	14.35	16.10	− 1.75
P4 + H_2_O	− 10.84	− 10.84	− 11.20	0.36
P5 + H_2_O	− 5.22	− 5.22	− 2.72	− 2.50

In the preceding study^54^, we offered all probable pathways for thiophene plus hydroperoxyl radical reaction by similar theoretical methods. It was proven that the reaction starts with the addition of HO_2_ to the α (more preferable) or β carbon atoms of the thiophene ring. The electrophilic attack of oxygen on the S atom was investigated as well. The reaction is simulated in the presence of a single water molecule to have an insight into the influence of a wet environment on activation energies and so the final rate constants.

To understand the role of water vapor in the title oxidation reaction, all electronic structure calculations are carried out with and without water to construct the respective potential energy surfaces (PESs). Comparing the PESs clears how water changes the reactivity of thiophene and hydroperoxyl radical when reacts with each other. For this, we must have the same reaction pathways for bare and water-assisted reactions. According to our previous research^54^, the HO_2_-addition pathways include two pre-reactive complexes. After the formation of the van-der-Waals complexes and passing through desired transition states, the path is completed. But, the presence of water is accompanied by more complexity due to several pre-reactive complexes having different relative energies and structures. In the sequel, we focused on the wet reaction and compare its results with the bare reaction.

#### Addition to β-C in the presence of water

When 1WHA and thiophene, and 1WTA and HO_2_ are gathered, two pre-reactive complexes CR-TS1-1WHA and CR-TS1-1WTB are formed, respectively, that are starting points of the β-C addition reaction. Since water can form ring-like structures, there is large stability in these complexes. The relative energies of CR-TS1-1WHA and CR-TS1-1WTB are − 16.24 and − 11.20 kcal mol^−1^, respectively, which are − 10.32 and − 5.28 kcal mol^−1^ more stable than CR1(bare complex). This result is important because water position determines how much energy is released. Then, according to water position, two transition states TS1-1WHA and TS1-1WTB are found for the addition of HO_2_ to β-C. The TS1-1WHA saddle point has a five-membered ring structure which is stabilized by two hydrogen bonds. In these bonds, water and HO_2_ play both hydrogen bond donor and hydrogen bond acceptor roles simultaneously. The relative energy of TS1-1WHA is 5.95 kcal mol^−1^, which is 9.65 kcal mol^−1^ more stable than the naked reaction due to the ring structure containing hydrogen bonds. Also, for the TS1-1WTB, a six-membered ring structure is obtained including one hydrogen bond and two van der Waals interactions. So, the stability of this transition state is slightly less than the TS1-1WHA. After the mentioned transition states the product complexes CP-TS1-1WHA1 and CP-TS1-1WTB1, are produced. The obtained product complexes have ring structures as well, which are confirmed by an AIM analysis. CP-TS1-1WHA1 has a five-membered ring structure with $\rho (rcp)$ = 0.0138 e bohr^−3^ and $\nabla^{2} \rho (rcp)$ = 0.0707 e bohr^−5^. And, CP-TS1-1WTB1 includes a six-membered ring structure with $\rho (rcp)$ = 0.0062 e bohr^−3^ and $\nabla^{2} \rho (rcp)$ = 0.0306 e bohr^−5^. Breaking the intermolecular interactions in both CP-TS1-1WHA1 and CP-TS1-1WTB1 is in accord with the P1 and water formation as final products. The thermodynamic parameters of P1 are ΔH^0^ = 6.49 kcal mol^−1^ and ΔG^0^ = 17.47 kcal mol^−1^. According to Tables S14 and S15, the Fukui values for radical addition of the O12 atom of hydroperoxyl radical to the C2 center of thiophene are − 0.535 and − 0.090 e in reactant species, respectively. These values are − 0.047 and − 0.126 e in TS1-1WHA and − 0.048 and − 0.139 e in TS-1WTB, respectively. A decrease in the value of $f_{X}^{0}$ (X = O and C) from reactants to the TSs is related to the formation of a covalent bond. This means that after the formation of a covalent bond between the reacting centers, the reactivity of these centers is decarded.

In the sequel, the reaction can be continued by the TS5-1WHA and TS5-1WTB saddle points. Firstly, without any barrier, the CP-TS1-1WHA1 and CP-TS1-1WTB1 can rearrange to the intermediate complexes CP-TS1-1WHA2 and CP-TS1-1WTB2, respectively. After that, an epoxy structure can obtain if the oxygen–oxygen bond of HO_2_ breaks via TS5-1WHA or TS5-1WTB. The ring structure of TS5-1WHA has five members containing two hydrogen bonds and one van-der-Waals interaction. The obtained values for the electronic charge density along with its Laplacian at the ring critical point are $\rho (rcp)$ = 0.2663 e bohr^−3^ and $\nabla^{2} \rho (rcp)$ = 0.0569 e bohr^−5^. Also, the existence of a six-membered ring with two hydrogen interactions and one van-der-Waals interaction in TS5-1WTB is confirmed by AIM analysis ($\rho (rcp)$ = 0.0878 e bohr^−3^ and $\nabla^{2} \rho (rcp)$ = 0.0371e bohr^−5^). The IRC path for the epoxy formation shows that the CP-TS5-1WHB and CP-TS5-1WTB are two final product complexes with some differences in their internal interactions. These differences are accompanied by relatively different relative energies for CP-TS5-1WHB (− 21.70 kcal mol^−1^) and CP-TS5-1WTB (− 20.32 kcal mol^−1^). It is better to note that CP-TS5-1WHB has a seven-membered ring structure ($\rho (rcp)$ = 0.0033 e bohr^−3^ and $\nabla^{2} \rho (rcp)$ = 0.0129 e bohr^−5^). AIM result demonstrated that in addition to two hydrogen bond interactions, this cyclic structure has an interaction between the O11 atom and the pi bond of C4 and C3 atoms. The CP-TS5-1WTB complex has also a seven-membered ring structure involving two hydrogen bonds. The computed AIM parameters for the CP-TS5-1WTB ring structure are $\rho (rcp)$ = 0.0030 e bohr^−3^ and $\nabla^{2} \rho (rcp)$ = 0.0152 e bohr^−5^. Also, for the discussed product complexes, the existence of an epoxy ring (a three-membered ring structure) between C1, O10, and C2 atoms is evaluated by AIM. The amounts of $\rho (rcp)$ and $\nabla^{2} \rho (rcp)$ for the epoxy ring in CP-TS5-1WHB and CP5-1WTB are 0.2023 and 0.2003 e bohr^−3^ and 0. 3171 and 0.3172 e bohr^−5^, respectively. The final products of these complexes are an oxirane-like bicyclic product plus hydroxyl radical (and water). From the thermodynamic point of view, the formation process of P5 (SCHOCHC_2_H_2_ + OH) is exothermic by -5.22 kcal mol^−1^ in standard enthalpy and spontaneous by -2.72 kcal mol^−1^ in standard Gibbs free energy.

In the other pathway, the HO_2_ addition to β-C is occurred by the OH part of HO_2_. This reaction starts by attacking the HO_2_ moiety to the β-C of the IWTB complex or the addition of the 1WHA moiety to the β-C of thiophene in which an oxygen atom is released for a very short time. Then, the free oxygen abstracts immediately the hydrogen atom of the attacking OH and forms a covalent bond with the O atom of OH. The transition states of this process are TS4-1WHA and TS4-1WTB. The relative energy for the water-assisted (TS4-1WHA and TS4-1WTB) and bear (TS4) saddle points is 46.51, 45.64, and 55.13 kcal mol^−1^, respectively. Geometrically, the TS4-1WTB transition state contains a seven-membered ring structure due to the establishment of one hydrogen bond and two van der Waals interactions ($\rho (rcp)$ = 0.0040 e bohr^−3^ and $\nabla^{2} \rho (rcp)$ = 0.0203 bohr^−5^). And there is only a strong interaction between shifting hydrogen and oxygen atom of water which is hydrogen type ($\rho (lcp)$ = 0.0376 e bohr^−3^ and $\nabla^{2} \rho (lcp)$ = 0.1028 e bohr^−5^). Also, the CP-TS4-1WTB has a seven-membered ring structure with one hydrogen bond (H14…O12) and one van der Waals interaction (O13…H7). The ring structure of the post-reactive complex CP-TS4-1WHA is similar to the CP-TS1-1WHA complex.

#### Addition to α-C in the presence of water

Before describing the reaction paths, it should be noted that the Fukui value for the α-C center ($f_{\alpha - C}^{0}$) is − 0.143 e which is higher than $f_{\beta - C}^{0}$(see Tables S18 and S19). This result indicates that the α-C center is more reactive than the β-C center. The addition to α-C is considered by two paths. The orientation of the water molecule makes the distinction between these paths. The first starts with CR-TS1-1WHA and occurs through TS2-1WHA. TS2-1WHA has a ring-like structure with five members containing two hydrogen bonds and one van der Waals interaction. This ring was analyzed by AIM theory. For the ring critical point, AIM parameters are $\rho (rcp)$ = 0.0154 e bohr^−3^, and $\nabla^{2} \rho (rcp)$ = 0.0831e bohr^−5^. For this reason, it is around 10.13 kcal mol^−1^ more stable than the TS2 (the corresponding transition state in the naked reaction). After TS2-1WHA, the post-reactive complex CP-TS2-1WHA is obtained in the IRC path with the relative energy of − 13.63 kcal mol^−1^ which is 9.50 kcal mol^−1^ more stable than the P2 (final) product. In the second path, CR-TS2-1WTA is the key per-reactive complex for the terminal oxygen (of HO_2_) addition to the α carbon of thiophene. Geometrically, it has a ring-like structure with six members, including two covalent bonds, one hydrogen bond, and two van der Waals interactions. This route is completed after surmounting the saddle point TS2-1WTA and the formation of the post-reactive complex CP-TS2-1WTA in the exit channel. The saddle point TS2-1WTA is 3.99 kcal mol^−1^ above the initial reactants. Also, the product complex CP-TS2-1WTA is 10.08 kcal mol^−1^ more stable than the initial reactants. The six-membered ring structure is formed in CP-TS2-1WTA. In this ring, the O13 of water has a van der Waals interaction with the hydrogen of thiophene, and also the hydrogen of water acts as a hydrogen bond acceptor with the middle oxygen of HO_2_. The P2 and water are exothermic and non-spontaneous products by − 2.77 and 8.53 kcal mol^−1^ in enthalpy and Gibbs free energy, respectively. Finally, it should be noted that between the α-C and β-C additions, the addition process to α-C is favorable both thermodynamically and kinetically. It is better to say that a similar tendency was also established in the addition of the oxygen atoms of NO_3_ and O_2_ radicals to thiophene^26,43^.

It has been shown that in the bare reaction, the thiophene ring-opening and elimination of the OH group may simultaneously happen after the addition of HO_2_ and generation of the P2 adduct, (a two-step reaction). In the presence of water, this process occurs through two pathways. In the first path, CP-TS2-1WHA rearranges to IN8a-1WHA and after passing through TS8a-1WHA converts to CP-TS8a-1WHA, and by dissociating the fragments of CP-TS8a-1WHA, the mentioned adduct is produced. The second path has a similar trend but the position of the water is different. The stationary points of this path after CP-TS2-1WTA are IN8a-1WTA, TS8a-1WTA, and CP-TS8a-1WTA, respectively. The obtained relative energies for IN8a-1WHA, TS8a-1WHA, and CP-TS8a-1WHA and IN8a-1WTA, TS8a-1WTA, and CP-TS8a-1WTA are − 15.67, 4.83, and − 26.06 kcal mol^−1^ and − 11.58, 3.12, and − 20.72 kcal mol^−1^, respectively. From a geometrical point of view, in the structure of TS8a, two hydrogen bonds and one van der Waals interaction are in a five-membered ring, but in TS8a-1WTA, one hydrogen bond and two van der Waals interactions are in a six-membered ring. The final product of the second step obtained from CP-TS8a-1WHA and CP-TS8a-1WTA is P4 (and water). P4 is the most stable and favorable product of this reaction (*∆H°* = − 10.84 kcal mol^−1^ and *∆G°* = − 11.20 kcal mol^−1^).

#### Addition to S in the presence of water

The influence of water on the electrophilic attack is investigated by HO_2_ addition on the S center of the 1WTA complex or the 1WHA complex addition on the S atom of thiophene that progresses from the TS3-1WTA and TS3-1WHA saddle points, respectively. The calculated Fukui parameter for the S center of thiophane and the terminal O center of HO_2_ in the electrophilic addition is − 0.267 and − 0.583 e in reactants, respectively. In TS3-1WHA and TS3-1WTA, the mentioned values are − 0.170 and − 0.044 e and − 0.177 and − 0.048 e, respectively, indicating the formation of a covalent bond. The pre-reactive complexes for this attack are CR-TS2-1WTA and CR-TS3-1WHA with relative energies of − 11.69 and − 15.26 kcal mol^−1^, respectively. The geometry of CR-TS2-1WTA has been discussed above. The CR-TS3-1WHA complex has an eight-membered ring-like structure in which the primary 1WHA complex is connected to thiophene by two van-der-Waals interactions. One of those interactions is located between the S atom of thiophene and the H14 atom of water, and the other is established between the O10 atom of HO_2_ and the H2 atom of thiophene (see Fig. 2).

From an energetic point of view, there is high stabilization for transition states after the addition of water. The calculated relative energies for TS3-1WTA and TS3-1WHA are predicted to lie 10.72 and 9.36 kcal mol^−1^ below the energy of TS3, respectively. Besides, CP-TS3-1WTA and CP-TS3-1WHA are 14.41 and 14.86 kcal mol^−1^ below CP3, indicating more stability for post-reactive complexes than respective TSs. The main reason for the differences in the stabilization energies of the mentioned transition states goes back to their ring structures. The seven-membered ring ($\rho (rcp)$ = 0.0040 e bohr^−3^ and $\nabla^{2} \rho (rcp)$ = 0.0184 e bohr ^−5^) in TS3-1WTA includes three van-der-Waals interactions and one hydrogen bond. The van der Waals interactions are between O13 (of water) and H6 (of thiophene), O11(of HO_2_) and O10 (of HO_2_), and O10 and S5 atoms. The hydrogen bond is between O11 and H14 atoms. But, in the TS3-1WHA structure, a six-membered ring is confirmed ($\rho (rcp)$ = 0.0044 e bohr^−3^ and $\nabla^{2} \rho (rcp)$ = 0.0195 e bohr^−5^) by AIM data with two van der Waals interactions and one hydrogen bond. The hydrogen atom (H14) of water also has a hydrogen bond with the O11 atom, and O11 has a van-der-Waals interaction with O10. In the TS3-1WHA structure, a six-membered ring is confirmed by AIM analysis with two van der Waals interactions and one hydrogen bond. The van der Waals interaction in TS3-1WHA is similar to TS3-1WTA, but the hydrogen bond is slightly different. The hydrogen atom of the water molecule acts as a donor and interacts with the O10 atom as an acceptor in the newly formed hydrogen bond. The characteristics of the new bond are $\rho (lcp)$ = 0.0287 e bohr^−3^ and $\nabla^{2} \rho (lcp)$ = 0. 0827 e bohr^−5^.

Similar trends are true about the energies of the product complexes CP-TS3-1WTA and CP-TS3-1WHA in comparison with CP3. The optimized structures of the CP-TS3-1WTA and CP-TS3-1WHA also indicate different ring structures in these complexes. Also, the calculated densities for these rings by AIM theory are ρ(r_rcp_) = 0.0018 e bohr^−3^ ($\nabla^{2} \rho (rcp)$ = 0.0087 e bohr^−5^) for CP-TS3-1WTA and $\rho (rcp)$ = 0.0033 e bohr^−3^ ($\nabla^{2} \rho (rcp)$ = 0.0131 e bohr^−5^) for CP-TS3-1WHA, respectively. The CP-TS3-1WTA has an eight-membered ring structure due to three established interactions. In this ring, the H12 atom of hydroxyl radical interacts with the connected oxygen (O10) to the sulfur atom. Also, the water molecule has one van der Waals interaction with the hydrogen of thiophene and one hydrogen bond with hydroxyl radical ($\rho (rcp)$ = 0.0309 e bohr^−3^ and $\nabla^{2} \rho (rcp)$ = 0.0858 e bohr^−5^). The interactions in the CP-TS3-1WHA complex are different from CP-TS3-1WTA. The eight-membered ring ($\rho (rcp)$ = 0.0033 e bohr^−3^ and $\nabla^{2} \rho (rcp)$ = 0.0131 e bohr^−5^) is seen due to the presence of three interactions including two hydrogen bonds and one van der Waals interaction. The hydrogen atom of water has an interaction with the attached oxygen to the sulfur atom. Also, the hydrogen of the OH radical interacts with the oxygen of the water molecule. In addition, the radical site of OH interacts with the C2 atom of thiophene (the van der Waals interaction). Finally, these channels are ended by rupturing CP-TS3-1WTA and CP-TS3-1WHA to thiophene-S-oxide, hydroxyl radical, and water moieties. The computed standard thermodynamic parameters for this reaction show that the formation process of the final adduct (P3) is endothermic by 14.35 kcal mol^−1^ and nonspontaneous by 16.10 kcal mol^−1^ in Gibbs free energy in the gas phase.

### Natural bond orbital (NBO) analysis

To interpret the charge transfer processes^54^ and characterize the inter-and intra-molecular interactions in molecular systems, a suitable quantum chemistry method is the NBO analysis. The energies of such interactions are expressed by the second-order perturbation theory between a donor NBO (i) and an acceptor NBO (j). The second-order stabilization energy E_2_ for delocalization of electrons between i and j NBOs can be calculated as:

3 $$E_{2} = \Delta E = q_{i} \left[ {\frac{{F_{i,j}^{2} }}{{\varepsilon_{i} - \varepsilon_{j} }}} \right]$$

where q_i_ is the orbital occupancy, *ε*_*i*_ and *ε*_*j*_ are diagonal elements of the Fock matrix, and *F*_*i,j*_ is the off-diagonal elements^77^. The results of the Fock matrix obtained by the NBO calculations for some stationary points are listed in Table 5.

**Table 5:** Analysis of the Fock matrix using the NBO calculations for some species in the thiophene plus hydroperoxyl radical reaction with and without a single water molecule.

Compound	Donor NBOs	Occupancy (e)	Acceptor NBOs	Occupancy (e)	E_2_ (kcal mol^−1^)
Thiophene	n_S(2)_	1.6299	π*(C_1_–C_2_)	0.2960	21.26
			π*(C_3_–C_4_)	0.2960	21.28
CR1	n_S(2)_	1.6173	π*(C_1_–C_2_)	0.2866	21.50
			π*(C_3_–C_4_)	0.3164	21.99
CR-TS1-1WHA	n_S(2)_	1.6238	π*(C_1_–C_2_)	0.3071	21.92
			π*(C_3_–C_4_)	0.2893	21.40
CR-TS1-1WTB	n_S(2)_	1.6199	π*(C_1_–C_2_)	0.3133	21.89
			π*(C_3_–C_4_)	0.2857	21.44
CR-TS2-1WTA	n_S(2)_	1.6196	π*(C_1_–C_2_)	0.3140	22.25
			π*(C_3_–C_4_)	0.2866	21.48
CR-TS4-1WTB	n_S(2)_	1.6172	π*(C_1_–C_2_)	0.3146	22.40
			π*(C_3_–C_4_)	0.2880	21.59
CR3	n_S(2)_	1.6422	π*(C_1_–C_2_)	0.2719	19.82
			π*(C_3_–C_4_)	0.2759	20.03
CR-TS3-1WHA	n_S(2)_	1.6239	π*(C_1_–C_2_)	0.2887	21.38
			π*(C_3_–C_4_)	0.3068	21.67
TS1	n_S(2)_	0.8668	π*(C_1_–C_2_)	0.1245	5.46
			π*(C_3_–C_4_)	0.2127	10.88
TS1-1WHA	n_O10(3)_	0.9905	π*(C1–C2)	0.2127	48.00
	n_S(2)_	0.8621	π*(C_1_–C_2_)	0.2134	5.68
	n_O12(3)_	0.8704	π*(C_1_–C_2_)	0.2134	47.94
TS1-1WTB	n_S(2)_	0.8656	π*(C_1_–C_2_)	0.2086	5.63
			π*(C_3_–C_4_)	0.1235	10.70
	n_O12(3)_	0.8761	π*(C_1_–C_2_)	0.2086	44.88
TS2	n_S(2)_	0.8472	π*(C_1_–C_2_)	0.2015	13.69
			π*(C_3_–C_4_)	0.1820	8.29
TS2-1WHA	n_S(2)_	0.8419	π*(C_1_–C_2_)	0.1967	13.72
	n_O12(3)_	0.9988	π*(C_1_–C_2_)	0.1967	26.26
TS2-1WTA	n_S(2)_	0.8490	π*(C_1_–C_2_)	0.2025	13.66
			π*(C_3_–C_4_)	0.1849	8.06
TS3	n_S(1)_	1.8935	π*(C_1_–C_2_)	0.2232	2.37
		0.9559	π*(C_3_–C_4_)	0.0607	0.94
TS3-1WTA	n_S(1)_	1.9045	π*(C_1_–C_2_)	0.1883	2.09
			π*(C_3_–C_4_)	0.2235	2.20
TS3-1WHA	n_S(1)_	1.9196	π*(C_3_–C_4_)	0.1151	2.19
			π*(C_1_–C_2_)	0.1151	2.19
TS4	n_S(2)_	0.9094	π*(C_1_–C_2_)	0.0897	9.68
			π*(C_3_–C_4_)	0.1758	0.40
TS4-1WTB	n_S(2)_	0.9069	π*(C_1_–C_2_)	0.0869	1.30

By referring to part I^54^, we stated that two strong interactions exist in thiophene due to the n_S(2)_ → π*(C_1_–C_2_) (E_2_ = 21.26 kcal mol^−1^) and n_S(2)_ → π*(C_3_–C_4_) (E_2_ = 21.28 kcal mol^−1^) transitions. In encountering thiophene with HO_2_ in wet (and dry) media, the energies of n_S(2)_ → π*(C_1_–C_2_) and n_S(2)_ → π*(C_3_–C_4_) transitions in CR-TS1-1WHA (and CR1) are 21.92 (and 21.50) and 21.40 (and 21.99), and in TS1-1WHA (TS1) are 5.68 (5.46) and 10.90 (10.88) kcal mol^−1^ respectively. The stabilization energies (E_2_) of the same transitions in TS1-1WTB are 5.63 and 10.70 kcal mol^−1^ respectively. These results show that for β addition, water alters the n_S(2)_ → π*(C_1_–C_2_) and n_S(2)_ → π*(C_3_–C_4_) transitions. There are also transitions between the lone pairs of the 11O atom and the unoccupied π*(C_1_–C_2_) and π*(C_3_–C_4_) orbitals in TS1-1WHA (TS1) and TS2-1WHA (TS2), respectively. These transitions vary the interactions of the lone pair electrons of the S atom and the unoccupied π*(C_1_–C_2_) and π*(C_3_–C_4_) orbitals. Thus, the activation energies of that TSs are changed. The stabilization energies E2 value for the n_O10(3)_ → π*(C_1_–C_2_) transition in TS1-1WHA and TS1-1WTB (and TS1) are 47.94 and 44.88 (48.00) kcal mol^−1^, respectively.

In TS2-1WHA (and TS2), the stabilization energies (E_2_) of the n_S(2)_ → π*(C_1_–C_2_) and n_S(2)_ → π*(C_3_–C_4_) transitions are 13.72 (13.69) and 8.48 (8.29) kcal mol^−1^ respectively, which show E_2_ values for this TS are increased in the presence of a single water molecule. Also, for the TS2-1WHA (TS2), the n_O12(3)_ → π* (C_1_–C_2_) transition has the E_2_ value of 26.26 (29.08) kcal mol^−1^ which is less than TS1-1WHA (TS1). So, the TS2-1WHA saddle point with the smallest E_2_ value for the n_O12(3)_ → π*(C_1_–C_2_) transition is the most stable TS of this reaction. A comparison of the relative energies of these two TSs shows that the TS2-1WHA is 10.12 kcal mol^−1^ more stable than TS2. Then, the charge-transfer delocalization for TS2-1WHA more than TS2.

The n_S(1)_ → π*(C_1_–C_2_) stabilization energy for TS3 is 2.37 kcal mol^−1^ but for TS3-1WTA and TS3-1WHA are reduced to 2.09 and 2.19 kcal mol^−1^, respectively. However, the n_S(1)_ → π*(C_3_–C_4_) stabilization energy for TS3 is 0.94 kcal mol^−1^ and for TS3-1WTA and TS3-1WHA is increased to 2.2 and 2.19 kcal mol^−1^, respectively. The n_S(2)_ → π*(C_1_–C_2_) and n_S(2)_ → π*(C_3_–C_4_) stabilization energy (E_2_) for TS4 are 9.68 and 0.4 kcal mol^−1^, respectively. But for TS4-1WTB, only the n_S(2)_ → π*(C_1_–C_2_) with 1.30 kcal mol^−1^ is seen in NBO analysis data.

Overall, all the new interactions by a water molecule can influence directly and indirectly the transitions between the π*(C_1_–C_2_) and π*(C_3_–C_4_) orbitals and lone pair electrons that can change the stability of the thiophene ring. Finally, we can conclude that in the presence of a single water molecule the E_2_ value is reduced for the discussed stationary points, relating to the involved intermolecular interactions (particularly hydrogen bonds by water molecules).

### Kinetic rate constant calculation

To find the effect of an added single water molecule on the kinetics of the title reaction, all rate constants of the studied pathways are calculated in terms of the canonical transition state theory^78^. That paths start with the formation of a pre-reactive complex and then pass through just a transition state and are finally released to the final products can be considered by the following reaction mechanism

4 $$R_{1} + R_{2} ...H_{2} O\underset{{k_{ - 1} }}{\overset{{k_{1} }}{\longleftrightarrow}}{\text{prereactive complex(CR)}}\mathop{\longrightarrow}\limits^{{k_{2} }}products$$

where R_1_ and R_2_ are reactants. k_1_ and k_−1_ are the rate constants of the association of reactants and dissociation of the pre-reactive collision complex, respectively. Also, k_2_ is the rate constant for the formation of final products from CR. The steady-state approximation should be used to calculate the overall reaction rate. Thus, the following rate expression by considering the equilibrium between CR and reactants is obtained.

5 $$k_{overall} = \frac{{k_{1} }}{{k_{ - 1} }}k_{2} = K_{eq} k_{2}$$

where the K_eq_ is the equilibrium constant of the first step in the reaction of eq. 4. The following equations are applied to calculate the rate constant of two-step reactions

6a $$K_{eq} = \frac{{k_{1} }}{{k_{ - 1} }} = \frac{{Q_{CR} }}{{Q_{{R_{1} }} Q_{{R_{2} }} }}\exp \left[ { - \frac{{E_{CR} - (E_{{R_{1} }} + E_{{R_{2} ...H_{2} O}} )}}{{k_{B} T}}} \right]$$

6b $$k_{2} = \sigma \kappa \frac{{k_{B} T}}{h}\frac{{Q_{TS} }}{{Q_{CR} }}\exp \left( { - \frac{{E_{TS} - E_{CR} }}{{k_{B} T}}} \right)$$

where k_B_, T, h $\kappa$, and σ are the Boltzmann constant, temperature, the Planck constant, the tunneling correction, and the symmetry factor, respectively. $Q_{{R_{1} }} Q_{{R_{2} ...H_{2} O}}$, $Q_{CR}$, and $Q_{TS}$ represent the partition functions of the reactants (that is shown by the product of separated reactants), pre-reactive complex, and transition states, respectively. The $E_{{R_{2} ...H_{2} O}} + E_{{R_{1} }}$, $E_{CR}$, and $E_{TS}$ indicate the total energy of the reactants, the complexes, and transition, respectively.

The tunneling effect is a quantum mechanical phenomenon that is defined as the ratio of barrier crossing rates obtained by quantum mechanics and classical mechanics. In this study, the unsymmetrical Eckart potential energy barrier has been used to simulate approximately the potential energy curve with high accuracy during tunneling^79^. The Eckart formula which is a potential function uses an unsymmetrical barrier to estimate the tunneling factor for computing precise rate constants theoretically. The Eckart formalism uses the following integral over the threshold energy E to calculate the tunneling factor.

7a $$\kappa = \exp \left( {\frac{{E_{b} }}{{k_{B} T}}} \right)\int\limits_{{E_{0} }}^{\infty } {P^{T} (E) \times \exp \left( { - \frac{E}{{k_{B} T}}} \right)} \frac{dE}{{k_{B} T}}$$

where *E*_*b*_ is the barrier energy of the reaction path, and *P*^*T*^*(E)* is the tunneling transmission probability.

7b $$P^{T} (E) = \frac{\cosh (a + b) - \cosh (a - b)}{{\cosh (a + b) + \cosh (c)}}$$

The tunneling transmission depends on three parameters that are as follows

7c $$a = \frac{{2\pi \sqrt {2\mu E} }}{h\alpha }$$

7d $$b = \frac{{2\pi \sqrt {2\mu (E - \Delta E)} }}{h\alpha }$$

7e $$c = \frac{{2\pi \sqrt {\left( {2\mu \beta - \frac{{\hbar^{2} \alpha^{2} }}{4}} \right)} }}{h\alpha }$$

where *ΔE* is calculated from the difference of energy between the sum of products and the sum of reactants which is called the reaction energy. The definition of other parameters is found elsewhere^80^. Also, we use the statistical Rice–Ramsperger–Kassel–Marcus (RRKM) theory to compute the rate constants of two-step reactions, which are considered to be continued by unimolecular reaction channels^81^.

To gain more useful insight into the influence of a single water molecule on the title reaction, all rate constants for the naked and water-assisted reactions are calculated by the abovementioned theories. For the above-discussed paths, the calculated rate constants are denoted by k_X_ as follows:

$$C_{4} H_{4} S + HO_{2} \mathop{\longrightarrow}\limits^{TS1}P1\quad \left( {{\text{k}}_{{{\text{P1}}}} } \right)$$

$$C_{4} H_{4} S + HO_{2} ...H_{2} O\mathop{\longrightarrow}\limits^{TS1 - 1WHA}CP - TS1 - 1WHA \to P1 + H_{2} O\quad \quad \left( {{\text{k}}_{{\text{P-1WHA}}} } \right)$$

$$C_{4} H_{4} S...H_{2} O + HO_{2} \mathop{\longrightarrow}\limits^{TS1 - 1WTB}CP1 - TS1 - 1WTB \to P1 + H_{2} O\quad \quad \left( {{\text{k}}_{{\text{P-1WTB}}} } \right)$$

$$C_{4} H_{4} S + HO_{2} \mathop{\longrightarrow}\limits^{TS4}P1\quad \left( {{\text{k}}_{{{\text{P1}}\prime }} } \right)$$

$$C_{4} H_{4} S + HO_{2} ...H_{2} O\mathop{\longrightarrow}\limits^{TS4 - 1WHA}CP - TS4 - 1WHA \to P1 + H_{2} O\quad \quad \left( {{\text{k}}_{{{\text{P1}}\prime {\text{-1WHA}}}} } \right)$$

$$C_{4} H_{4} S...H_{2} O + HO_{2} \mathop{\longrightarrow}\limits^{TS4 - 1WTB}CP - TS4 - 1WTB \to P1 + H_{2} O\quad \left( {{\text{k}}_{{{\text{P1}}\prime {\text{-1WTB}}}} } \right)$$

$$C_{4} H_{4} S + HO_{2} \mathop{\longrightarrow}\limits^{TS2}P1\quad \left( {{\text{k}}_{{{\text{P2}}}} } \right)$$

$$C_{4} H_{4} S + HO_{2} ...H_{2} O\mathop{\longrightarrow}\limits^{TS2 - 1WHA}CP2 - TS2 - 1WHA \to P2 + H_{2} O\quad \quad \left( {{\text{k}}_{{\text{P2-1WHA}}} } \right)$$

$$C_{4} H_{4} S...H_{2} O + HO_{2} \mathop{\longrightarrow}\limits^{TS2 - 1WTA}CP2 - TS2 - 1WTA \to P2 + H_{2} O\quad \quad \left( {{\text{k}}_{{\text{P2-1WTA}}} } \right)$$

$$C_{4} H_{4} S + HO_{2} \mathop{\longrightarrow}\limits^{TS3}CP3 \to P3\,\,\,\,\,\,\,\,\,\,\,\,\,\left( {{\text{k}}_{{{\text{P3}}}} } \right)$$

$$C_{4} H_{4} S + HO_{2} ...H_{2} O\mathop{\longrightarrow}\limits^{TS3 - 1WHA}CP - TS3 - 1WHA \to P3 + H_{2} O\,\,\,\,\,\,\,\,\,\,\,\, \left( {{\text{k}}_{{\text{P3-1WHA}}} } \right)$$

$$C_{4} H_{4} S...H_{2} O + HO_{2} \mathop{\longrightarrow}\limits^{TS3 - 1WTA}CP3 - TS3 - 1WTA \to P3 + H_{2} O\,\,\,\,\,\,\,\,\,\,\,\,\,\,\,\,\,\left( {{\text{k}}_{{\text{P3-1WTA}}} } \right)$$

$$C_{4} H_{4} S + HO_{2} \mathop{\longrightarrow}\limits^{TS2}P2\mathop{\longrightarrow}\limits^{TS8a}CP8a \to P4\,\,\,\,\,\,\,\,\,\, \left( {{\text{k}}_{{{\text{P4}}}} } \right)$$

$$\begin{gathered} C_{4} H_{4} S + HO_{2} ...H_{2} O\mathop{\longrightarrow}\limits^{TS2 - 1WHA}CP2 - TS2 - 1WHA\mathop{\longrightarrow}\limits^{TS8a - 1WHA} \hfill \\ CP - TS8a - 1WHA \to P4 + H_{2} O\,\,\,\,\,\,\,\,\,\,\,\,\,\,\,\,\,\,\,\,\,\,\,\,\,\,\,\,\,\,\,\,\,\,\,\,\left( {{\text{k}}_{{\text{P4-1WHA}}} } \right) \hfill \\ \end{gathered}$$

$$\begin{gathered} C_{4} H_{4} S...H_{2} O + HO_{2} \mathop{\longrightarrow}\limits^{TS2 - 1WTA}CP2 - TS2 - 1WTA\mathop{\longrightarrow}\limits^{TS8a - 1WTA} \hfill \\ CP8a - TS8a - 1WTA \to P4 + H_{2} O\quad \quad \left( {{\text{k}}_{{\text{P4-1WTA}}} } \right) \hfill \\ \end{gathered}$$

$$C_{4} H_{4} S + HO_{2} \mathop{\longrightarrow}\limits^{TS5}P1\mathop{\longrightarrow}\limits^{TS5}CP5 \to P5\quad \quad \left( {{\text{k}}_{{{\text{P5}}}} } \right)$$

$$\begin{gathered} C_{4} H_{4} S + HO_{2} ...H_{2} O\mathop{\longrightarrow}\limits^{TS1 - 1WHA}CP - TS1 - 1WHA\mathop{\longrightarrow}\limits^{TS5W - 1WHA} \hfill \\ CP - TS5W - 1WHA \to P5 + H_{2} O\quad \quad \left( {{\text{k}}_{{\text{P5-1WHA}}} } \right) \hfill \\ \end{gathered}$$

$$\begin{gathered} C_{4} H_{4} S...H_{2} O + HO_{2} \mathop{\longrightarrow}\limits^{TS1 - 1WTB}CP1 - TS1 - 1WTB\mathop{\longrightarrow}\limits^{TS5 - 1WTB} \hfill \\ CP5 - TS5 - 1WTB \to P5 + H_{2} O\quad \quad \left( {{\text{k}}_{{\text{P5-1WTB}}} } \right) \hfill \\ \end{gathered}$$

The obtained rate data are reported in Table S9 and S10. Also, for easier comparison, the ratios of rate constants in the presence and absence of water are collected in Table 6.

**Table 6:** The ratios of the computed rate constants at the BD(T) level for the water-assisted paths to the respective ones in the bare reaction.

T [K]	k_P-1WHA_/k_P1_	k_P1-1WTB_/k_P1_	k_P1′-1WHA_/k_P1′_	k_P1′-1WTB_/ k_P1′_	k_P2-1WHA_/k_P2_	k_P2-1WTA_/k_P2_	k_P3-1WHA_/k_P3_	k_P3-1WTA_/k_P3_	k_P4-1WHA_/k_P4_	k_P4-1WTA_/k_P4_	k_P5-1WHA_/k_P5_	k_P5-1WTB_/k_P5_
300	3.58E−02	1.01E+02	2.64E−01	5.18E+03	8.32E−02	1.45E+01	5.57E−02	1.07E+03	3.35E−02	2.66E+01	1.61E−03	1.97E+02
400	4.42E−02	1.11E+01	5.40E−02	1.21E+02	8.29E−02	1.74E+00	8.36E−02	3.10E+01	4.74E−02	2.25E+00	3.99E−03	1.52E+01
500	4.97E−02	2.91E+00	5.70E−02	1.59E+01	8.27E−02	4.76E−01	1.07E−01	3.55E+00	5.58E−02	5.14E−01	7.11E−03	3.03E+00
600	5.37E−02	1.18E+00	6.70E−02	4.23E+00	8.25E−02	1.98E−01	1.27E−01	8.23E−01	6.08E−02	1.89E−01	1.10E−02	9.98E−01
700	5.67E−02	6.12E−01	7.79E−02	1.65E+00	8.24E−02	1.06E−01	1.44E−01	2.87E−01	6.41E−02	9.05E−02	1.56E−02	4.45E−01
800	5.90E−02	3.75E−01	8.86E−02	8.17E−01	8.23E−02	6.57E−02	1.58E−01	1.30E−01	6.86E−02	5.16E−02	2.06E−02	2.43E−01
900	6.08E−02	2.55E−01	9.88E−02	4.73E−01	8.22E−02	4.53E−02	1.70E−01	6.97E−02	7.32E−02	3.33E−02	2.55E−02	1.52E−01
1000	6.23E−02	1.88E−01	1.08E−01	3.06E−01	8.21E−02	3.37E−02	1.80E−01	4.23E−02	7.67E−02	2.34E−02	3.01E−02	1.05E−01
1100	6.35E−02	1.46E−01	1.17E−01	2.14E−01	8.21E−02	2.64E−02	1.90E−01	2.81E−02	7.92E−02	1.75E−02	3.43E−02	7.80E−02
1200	6.45E−02	1.18E−01	1.26E−01	1.59E−01	8.20E−02	2.15E−02	1.97E−01	2.00E−02	8.11E−02	1.38E−02	3.81E−02	6.12E−02
1300	6.54E−02	9.87E−02	1.33E−01	1.24E−01	8.20E−02	1.81E−02	2.04E−01	1.50E−02	8.25E−02	1.13E−02	4.15E−02	5.00E−02
1400	6.62E−02	8.46E−02	1.41E−01	1.00E−01	8.20E−02	1.56E−02	2.11E−01	1.17E−02	8.36E−02	9.53E−03	4.45E−02	4.22E−02
1500	6.69E−02	7.41E−02	1.47E−01	8.32E−02	8.19E−02	1.37E−02	2.16E−01	9.43E−03	8.44E−02	8.23E−03	4.72E−02	3.65E−02
1600	6.75E−02	6.59E−02	1.54E−01	7.08E−02	8.19E−02	1.23E−02	2.21E−01	7.82E−03	8.51E−02	7.25E−03	4.96E−02	3.22E−02
1700	6.80E−02	5.95E−02	1.59E−01	6.14E−02	8.19E−02	1.11E−02	2.26E−01	6.62E−03	8.56E−02	6.48E−03	5.17E−02	2.89E−02
1800	6.85E−02	5.43E−02	1.65E−01	5.41E−02	8.19E−02	1.02E−02	2.30E−01	5.71E−03	8.60E−02	5.87E−03	5.35E−02	2.63E−02
1900	6.90E−02	5.00E−02	1.70E−01	4.83E−02	8.19E−02	9.40E−03	2.33E−01	5.01E−03	8.63E−02	5.37E−03	5.52E−02	2.41E−02
2000	6.94E−02	4.65E−02	1.75E−01	4.36E−02	8.19E−02	8.75E−03	2.37E−01	4.44E−03	8.66E−02	4.97E−03	5.66E−02	2.24E−02
2100	6.97E−02	4.35E−02	1.79E−01	3.98E−02	8.19E−02	8.21E−03	2.40E−01	3.99E−03	8.68E−02	4.62E−03	5.79E−02	2.09E−02
2200	7.01E−02	4.09E−02	1.84E−01	3.66E−02	8.19E−02	7.74E−03	2.43E−01	3.62E−03	8.70E−02	4.33E−03	5.90E−02	1.96E−02
2300	7.04E−02	3.87E−02	1.88E−01	3.39E−02	8.19E−02	7.34E−03	2.45E−01	3.31E−03	8.71E−02	4.08E−03	6.00E−02	1.86E−02
2400	7.06E−02	3.68E−02	1.91E−01	3.16E−02	8.19E−02	6.99E−03	2.48E−01	3.05E−03	8.72E−02	3.87E−03	6.08E−02	1.76E−02
2500	7.09E−02	3.51E−02	1.95E−01	2.97E−02	8.19E−02	6.68E−03	2.50E−01	2.83E−03	8.72E−02	3.68E−03	6.15E−02	1.68E−02
2600	7.11E−02	3.36E−02	1.98E−01	2.80E−02	8.19E−02	6.41E−03	2.52E−01	2.64E−03	8.71E−02	3.52E−03	6.20E−02	1.61E−02
2700	7.14E−02	3.23E−02	2.02E−01	2.65E−02	8.19E−02	6.17E−03	2.54E−01	2.48E−03	8.71E−02	3.37E−03	6.25E−02	1.54E−02
2800	7.16E−02	3.11E−02	2.05E−01	2.52E−02	8.19E−02	5.95E−03	2.56E−01	2.33E−03	8.70E−02	3.24E−03	6.28E−02	1.49E−02
2900	7.18E−02	3.01E−02	2.07E−01	2.40E−02	8.19E−02	5.76E−03	2.58E−01	2.21E−03	8.69E−02	3.12E−03	6.31E−02	1.43E−02
3000	7.20E−02	2.91E−02	2.10E−01	2.30E−02	8.19E−02	5.58E−03	2.59E−01	2.10E−03	8.68E−02	3.02E−03	6.34E−02	1.39E−02

As seen in Fig. 4, for the calculated rate constants at the mentioned temperature range, the non-Arrhenius behavior is observed. Therefore, the three-parameter equation $k = A(\frac{T}{300})^{m} \exp ( - \frac{{E_{b} }}{RT})$ is used to fit the calculated temperature dependence of rate constants. All the following rate expressions are bimolecular in the unit of L mol^−1^ s^−1^.

8 $$k_{P1} = 3.81 \times 10^{5} \left( \frac{T}{300} \right)^{(3.15 \pm 0.01)} \exp \left[ { - \frac{{(7.11 \pm 0.01)\,\,{\text{kcal}}\,\,{\text{mol}}^{ - 1} }}{RT}} \right]$$

9 $$k_{{P1{\text{-}}1WHA}} = 3.04 \times 10^{4} \left( \frac{T}{300} \right)^{(3.14 \pm 0.02)} \exp \left[ { - \frac{{(7.34 \pm 0.02)\,\,{\text{kcal}}\,\,{\text{mol}}^{ - 1} }}{RT}} \right]$$

10 $$k_{{P1{\text{-}}1WTB}} = 5.49 \times 103\left( \frac{T}{300} \right)^{(3.03 \pm 0.02)} \exp \left[ { - \frac{{(4.47 \pm 0.02)\,\,\,{\text{kcal}}\,\,{\text{mol}}^{ - 1} }}{RT}} \right]$$

11 $$k_{{P1^{\prime}}} = 3.35 \times 10^{4} \left( \frac{T}{300} \right)^{(4.08 \pm 0.10)} \exp \left[ { - \frac{{(25.86 \pm 0.09)\,\,{\text{kcal}}\,\,{\text{mol}}^{ - 1} }}{RT}} \right]$$

12 $$k_{{P1^{\prime}{\text{-}}1WHA}} = 2.40 \times 10^{2} \left( \frac{T}{300} \right)^{(5.50 \pm 0.32)} \exp \left[ { - \frac{{(24.96 \pm 0.28)\,\,{\text{kcal}}\,\,{\text{mol}}^{ - 1} }}{RT}} \right]$$

13 $$k_{{P1^{\prime}{\text{-}}1WTB}} = 9.29 \times 10^{1} \left( \frac{T}{300} \right)^{(4.39 \pm 0.14)} \exp \left[ { - \frac{{(21.56 \pm 0.13)\,\,{\text{kcal}}\,\,{\text{mol}}^{ - 1} }}{RT}} \right]$$

14 $$k_{P2} = 4.02 \times 10^{5} \left( \frac{T}{300} \right)^{(3.13 \pm 0.01)} \exp \left[ { - \frac{{(5.09 \pm 0.01)\,\,{\text{kcal}}\,\,{\text{mol}}^{ - 1} }}{RT}} \right]$$

15 $$k_{{P2{\text{-}}1WHA}} = 3.41 \times 10^{4} \left( \frac{T}{300} \right)^{(3.12 \pm 0.02)} \exp \left[ { - \frac{{(5.09 \pm 0.02)\,\,{\text{kcal}}\,\,{\text{mol}}^{ - 1} }}{RT}} \right]$$

16 $$k_{{P2{\text{-}}1WTA}} = 1.17 \times 10^{3} \left( \frac{T}{300} \right)^{(3.04 \pm 0.02)} \exp \left[ { - \frac{{(2.53 \pm 0.02)\,\,{\text{kcal}}\,\,{\text{mol}}^{ - 1} }}{RT}} \right]$$

17 $$k_{P3} = 1.03 \times 10^{6} \left( \frac{T}{300} \right)^{(3.50 \pm 0.04)} \exp \left[ { - \frac{{(16.18 \pm 0.03)\,\,{\text{kcal}}\,\,{\text{mol}}^{ - 1} }}{RT}} \right]$$

18 $$k_{P3 {\text{-}} 1WHA} = 2.87 \times 10^{5} \left( \frac{T}{300} \right)^{(3.53 \pm 0.04)} \exp \left[ { - \frac{{(16.66 \pm 0.03)\,\,{\text{kcal}}\,\,{\text{mol}}^{ - 1} }}{RT}} \right]$$

19 $$k_{{P3{\text{-}}1WTA}} = 7.12 \times 10^{2} \left( \frac{T}{300} \right)^{(3.34 \pm 0.02)} \exp \left[ { - \frac{{(11.88 \pm 0.02)\,\,{\text{kcal}}\,\,{\text{mol}}^{ - 1} }}{RT}} \right]$$

20 $$k_{P4} = 3.41 \times 10^{5} \left( \frac{T}{300} \right)^{(3.16 \pm 0.02)} \exp \left[ { - \frac{{(5.66 \pm 0.02)\,\,{\text{kcal}}\,\,{\text{mol}}^{ - 1} }}{RT}} \right]$$

21 $$k_{{P4{\text{-}}1WHA}} = 4.76 \times 10^{4} \left( \frac{T}{300} \right)^{(3.02 \pm 0.05)} \exp \left[ { - \frac{{(6.09 \pm 0.04){\text{kcal}}\,\,{\text{mol}}^{ - 1} }}{RT}} \right]$$

22 $$k_{{P4{\text{-}}1WTA}} = 4.38 \times 10^{2} \left( \frac{T}{300} \right)^{(3.09 \pm 0.02)} \exp \left[ { - \frac{{(2.76 \pm 0.02)\,\,{\text{kcal}}\,\,{\text{mol}}^{ - 1} }}{RT}} \right]$$

23 $$k_{5} = 4.98 \times 10^{5} \left( \frac{T}{300} \right)^{(3.08 \pm 0.08)} \exp \left[ { - \frac{{(8.23 \pm 0.07)\,\,{\text{kcal}}\,\,{\text{mol}}^{ - 1} }}{RT}} \right]$$

24 $$k_{{P5{\text{-}}1WHA}} = 4.68 \times 10^{4} \left( \frac{T}{300} \right)^{(3.14 \pm 0.13)} \exp \left[ { - \frac{{(9.47 \pm 0.11)\,\,{\text{kcal}}\,\,{\text{mol}}^{ - 1} }}{RT}} \right]$$

25 $$k_{{P5{\text{-}}1WTB}} = 1.42 \times 10^{3} \left( \frac{T}{300} \right)^{(3.29 \pm 0.04)} \exp \left[ { - \frac{{(4.85 \pm 0.04){\text{kcal}}\,\,{\text{mol}}^{ - 1} }}{RT}} \right]$$

Also, the plots of these fitted equations are shown in Fig. 4.

These results demonstrated that the water-assisted reaction has a similar trend the same as the naked reaction. Our computed rate constants show that the addition to the α carbon, α-C, is more favorable than β-C in the studied temperature range. For access to more detail about the naked reaction please see our previous paper^54^. It should be noted that according to the TST theory, in addition to the barrier energy of a path, the ratio of the total partition function of a saddle point to the product of the partition function of reactants is important. It shall be emphasized to this point that this ratio for the water-assisted reactions is smaller than the corresponding bare reactions.

As aforementioned, a single water molecule has different effects due to different positions. So, the rate constants k_P1-1WHA_ and k_P1-1WTB_ have different behavior by temperature variation. k_P1-1WTB_ is more favorable than k _P1-1WHA_ in a temperature range of 300–1600 K but above 1600 K, the rate constant k_P1-1WHA_ is more. Also, the ratio of rate constants (k_water_/k_nacked_) for k_P1-1WHA_/k_P1_ (and k_P1-1WTB_/k_P1_) is 3.58E−02 (1.01E+02), 4.97E−02 (2.92E+00), and 5.90E−02 (3.57E−01) at 300, 500, and 800 K, and is 6.23E−02 (1.88E−01), 6.69E−02 (7.41E−02), and 7.20E−02 (2.91E−02) and, at 1000, 1500, and 3000 K, respectively. These results show a meaningful difference in the values of computed rate constants for the P1 generation pathways in wet and dry media at high temperatures. Other paths to produce P1 include TS4, TS4-1WHA, and TS4-1WTB. The calculated rate constants for these paths (k_P1′,_ k_P1′-1WHA,_ and k_P1′-1WTB_) are small due to having large energy barriers. It is better to say that the P1 formation through the mentioned paths in the presence and absence of water is not kinetically important up to 1500 K since the ratio of rates of k_P1′-1WHA_ (k_P1′-1WTB_) to k_P1′_ is small. This shows that this path even in the presence of water does not occur at low and moderate temperatures. The value of the rate constant for the P2 product, k_P2-1WTA,_ is more than all pathways of the water-assisted reactions that coincide with the corresponding naked reaction because addition to the α carbon is the main reaction pathway among all addition reactions. Also, the computed rate constants for the HO_2_ addition to the S atom (k_P3-1WHA_ and k_P3-1WTA_) in the water-assisted reaction indicate that the same as the respective naked reaction (k_3_), this reaction does not occur at low temperatures. However, the computed rate constant for k_P3-1WTA_ is higher than k_P3-1WHA_ up to 800 K and after that the rate of P3 formation via the path that is procced by the 1WHA complex is high. The ratio k_P3-1WHA_/k_P3_ (k_P3-1WTA_/k_3_) is 5.57E−02 (1.07E+03), 1.58E−01 (1.30E−01), 2.16E−01 (943E−03), 2.59E−01 (2.10E−03) at 300, 800, 1500, and 3000 K, respectively (see Table 6).

For two-step reactions, the rate constant of product formation is computed in both dry and wet media (see Tables S9 and S10). For P4 generation, the ratio of rate constant in the presence and absence of a water molecule k_P4-1WHA_/k_4_ (k_P4-1WTA_/k_4_) is 3.35E−02 (2.66E+01), 8.23E−03 (4.72E−02), and 8.68E−02 (3.02E−03) at 300, 1500, and 3000 K, respectively. These results demonstrate that the same as other paths the product formation through 1WHA formation has a lower rate constant at low temperatures. Regarding the ratio k_P5-1WHA_/k_5_, the results indicate that these ratios are smaller than others at low temperatures. The obtained value for k_P5-1WHA_/k_5_ is 3.61E−03, 3.99E−03, 7.11E−02 and at 300, 400, and 500 K, respectively. But, for k_P5-1WTB_/k_5_ the same values are 1.97E+02, 1.52E−01 and 3.03E+00, respectively. Finally, it can be concluded that there is a remarkable difference in the computed rate constants of a product formation in wet media due to the orientation of the water molecule. Also, there is a meaningful difference between the computed rate constants of the argued paths in wet and dry media because of a change in barrier energy over the 300–3000 K temperature range.

## Conclusion

This work was accomplished to prove the crucial role of a water molecule in atmospheric reactions in which one component is an aromatic compound. To establish the impact of water, the addition mechanism in the HO_2_…H_2_O + thiophene system was selected as an example. The reaction begins with the association of the 1WHA and thiophene and 1WTA and thiophene, and then the formation of different pre-reactive complexes in the initial stage of addition reactions. Then, the reactions go on with adding HO_2_ radical to the α and β carbons and also the sulfur atom. The obtained reaction pathways showed that the reaction mechanism with a single water molecule is more complicated than the naked reaction. Our results made clear that the presence of water molecules leads to the creation of ring-like structures. These ring structures were confirmed by AIM analysis and were observed due to hydrogen bonds and van der Waals interactions in all prereactive, post-reactive, and transition state structures. The main consequence of the strong interactions in ring structures was to lead to high stabilization of the stationary points. The amounts of stabilization energies for TS1-1WHA (TS1-1WTB), TS2-1WHA (TS2-1WTA), TS3-1WTA (TS3-1WHA), TS4-1WTB, TS5-1WHA (TS5-1WTB), and TS8a-1WHA (TS8a-1WTA) are 9.65 (7.43), 10.13 (7.53), 9.36 (10.72), 8.62 (9.49), 7.75 (9.12), and 8.96 (10.68) kcal mol^−1^, respectively. Although the ratio of the partition function $\frac{{Q_{TS} }}{{Q_{{R_{1} }} Q_{{R_{2} }} }}$ is similar for all paths, the amount of stabilization energy due to the presence of water in the 1WHA complex is higher than that of the water-assisted TSs. Thus, these paths have a smaller rate constant than the respective naked reactions. Despite this, the inverse treatment is seen for the paths that start with the 1WTA and 1WTB complexes. Therefore, the results of this study show that the presence of water accelerates or does not accelerate the rate constant of the addition pathways which is related to the reacting primary complex formed between water and one of the reactants.

## Supplementary Information

Supplementary Information.

## Data Availability

All data generated or analysed during this study are included in this published article [and its supplementary information files].
