# Supplementary material for: Impact of a single water molecule on the atmospheric oxidation of thiophene by hydroperoxyl radical
Source: Sci Rep. 2022 Nov 8;12:18959. doi: 10.1038/s41598-022-22831-8 (PMC9643398; doi:10.1038/s41598-022-22831-8)
Supplement: Supplementary file 1 — Supplementary Information. [file 41598_2022_22831_MOESM1_ESM.docx]

**Impact of a single water molecule on the atmospheric oxidation of thiophene by hydroperoxyl radical**

Hamed Douroudgari^*^, Maryam Seyed Sharifi, Morteza Vahedpour^*^

Department of Chemistry, University of Zanjan, PO Box 38791-45371, Zanjan, Iran

E-mail:  [douroudgari@znu.ac.ir](mailto:%20douroudgari@znu.ac.ir)

E-mail:  [vahed@znu.ac.ir](mailto:%20vahed@znu.ac.ir%20)

**Figure S1.** Other optimized structures of the thiophene plus hydroperoxyl radical reaction in the presence of a single water molecule.

**Table S1.** Calculated spin contamination values ⟨S^2^⟩ before and after spin annihilation at the B3LYP/6-31+G(d,p) level.

**Table S2.** Topological parameters of the stationary points in the presence of water for C_4_H_4_S + HO_2_ reaction computed at the B3LYP/6-31+ g(d,p) level.

**Table S3.** The thermodynamic parameters (kcal mol^-1^) at 298 K for all stationary points of the water-assisted reaction calculated at the B3LYP method.

**Table S4.** The thermodynamic parameters (kcal mol^-1^) at 298 K for all stationary points of the water-assisted reaction calculated at the CCSD(T) method.

**Table S5.** Total energies (in Hartree) of the reactants, products, intermediates, and transition states involved in the bare reaction at different levels of theory.

**Table S6.** The calculated zero point and thermodynamic (298.15 K) corrections of the reactants, products, intermediates, and transition states involved in the bare reaction using the B3LYP/6-31+g(d,p) level of theory.

**Table S7.** The total energies for all components of the title reaction in the main water-assisted pathways using the B3LYP, CBS-QB3, CCSD(T), and BD(T) methods (unit of all numbers is Hartree).

**Table S8.** The calculated zero point and thermodynamic (298.15 K) corrections for all components of the title reaction in the main water-assisted pathways using the B3LYP method.

**Table S9.** The relative energies corrected by the zero energy and thermodynamic (298.15 K) parameters for all components of the title reaction in the other water-assisted pathways computed by the B3LYP method (unit of all numbers is kcal mol^-1^).

**Table S10** The absolute energies, zero point energies, thermodynamic corrections, and entropies (298.15 K) for all components of the title reaction in the other water-assisted pathways computed by the B3LYP method (unit of E_Absolute_, ZPE, E_Correction_, H_Correction_, and G_Correction_, is Hartree and unit of S is cal mol^-1^).

**Table S11.** Imaginary Frequency of obtained transition states in the naked and water-assisted reactions.

**Table S13.** The calculated rate constants (in L mol^−1^s^−1^) for water-assisted pathways of the C_4_H_4_S + HO_2_ reaction calculated at the BD(T)/6-31+g(d,p) level.

**Table S14:** The reactivity parameter values by means of the condensed Fukui function for the stationary points of the P1 and P5 formation path started from the 1WHA and thiophene species.

**Table S15:** The reactivity parameter values by means of the condensed Fukui function for the stationary points of the P1 and P5 formation path started from the 1WTB and HO_2_ species.

**Table S16:** The reactivity parameter values by means of the condensed Fukui function for the stationary points of the P1 formation path proceed by the TS4-1WHA saddle point.

**Table S17:** The reactivity parameter values by means of the condensed Fukui function for the stationary points of the P1 formation path proceed by the TS4-1WTB saddle point.

**Table S18:** The reactivity parameter values by means of the condensed Fukui function for the stationary points of the P2 and P4 formation path started from the 1WHA and thiophene species.

**Table S19:** The reactivity parameter values by means of the condensed Fukui function for the stationary points of the P2 and P4 formation path started from the HO_2_ and 1WTA species.

**Table S20:** The reactivity parameter values by means of the condensed Fukui function for the stationary points of the P3 formation path started from the thiophene and 1WHA species.

**Table S21:** The reactivity parameter values by means of the condensed Fukui function for the stationary points of the P3 formation path started from the HO_2_ + 1WTA species.

**Table S22**. Optimized cartesian Coordinates of all stationary points of thiophene and hydroperoxyl radical reaction in the presence and absence of water molecule.

**Figure S1.** Other optimized structures of the thiophene plus hydroperoxyl radical reaction in the presence of a single water molecule.


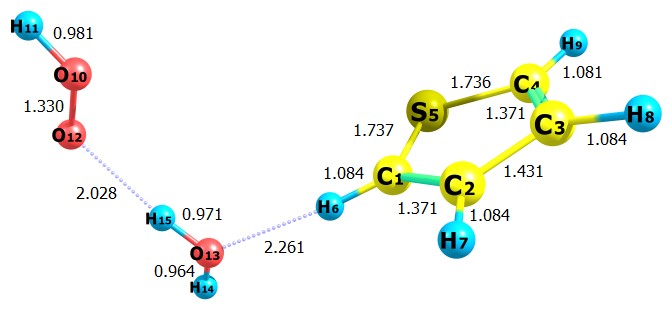

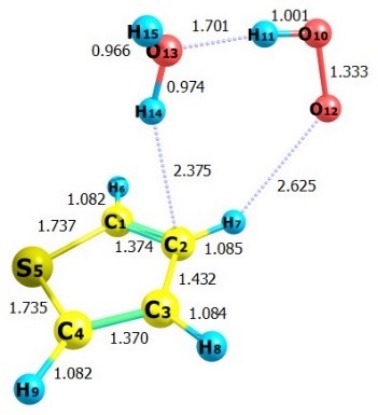


CR-TS1-1WHB CR-TS1-1WTA


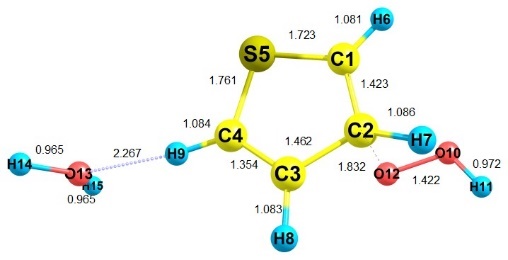

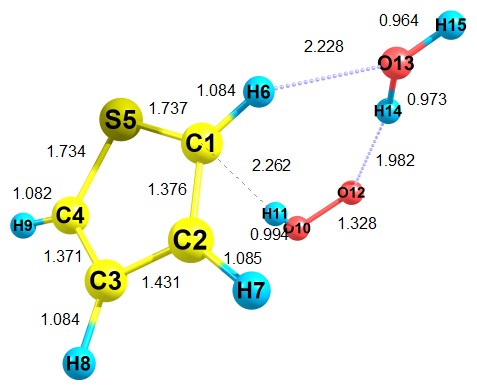


CR-TS1-1WTA2 CR-TS2-1WHB


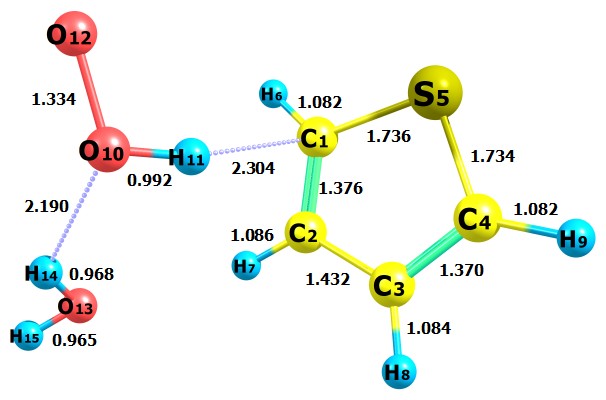

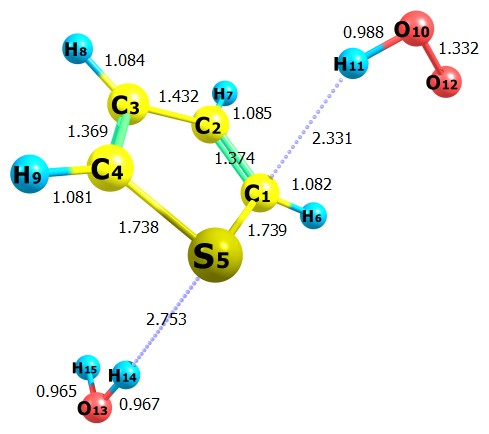


CR-TS2-1WHC CR-TS2-1WTD


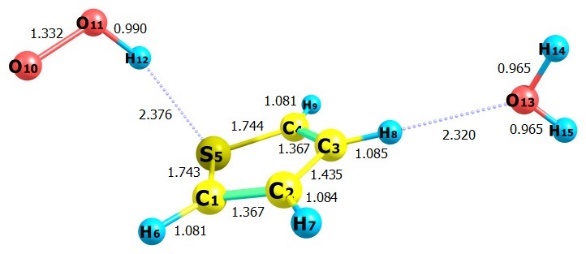

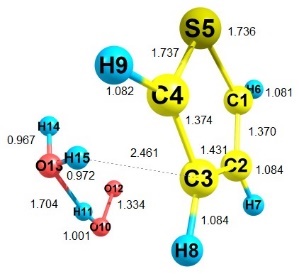


CR-TS3-1WTB CR-TS4-1WHA


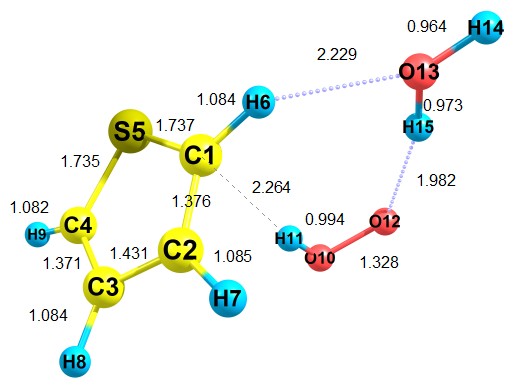

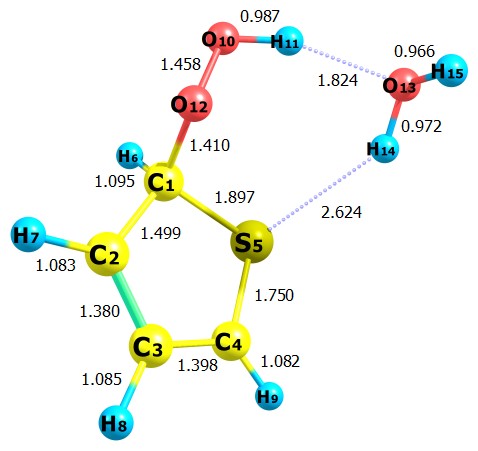


CR-TS4-1WTA IN8a-1WHB2


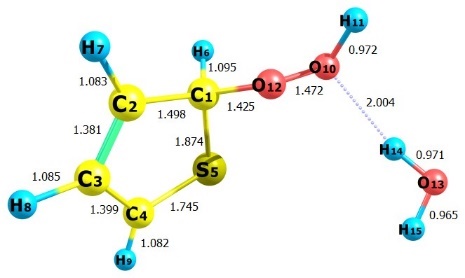

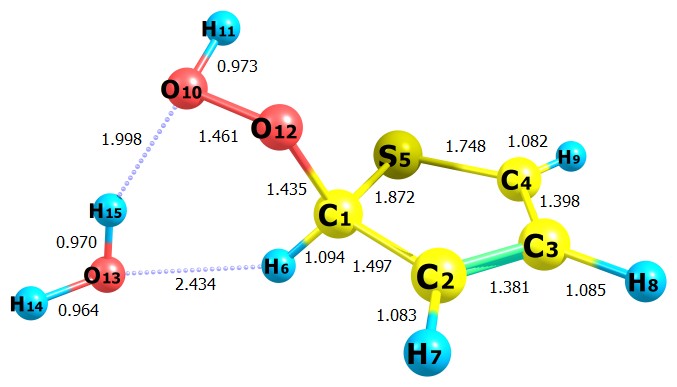


IN8a-1WHC IN8a-1WHC2


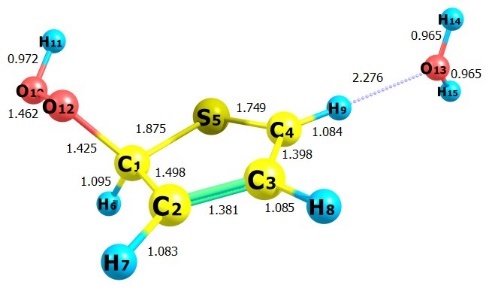

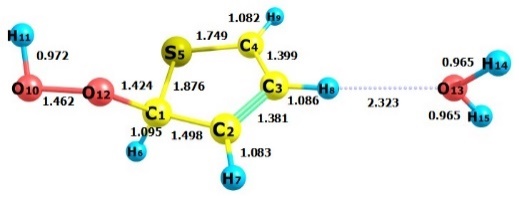


IN8a-1WTA2 IN8a-1WTB2


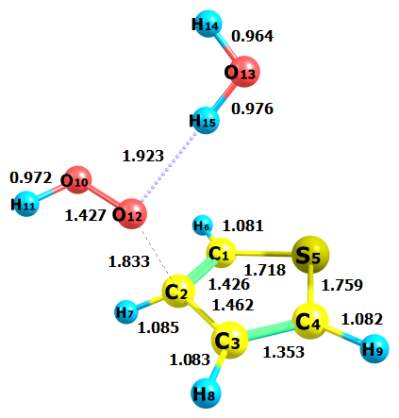

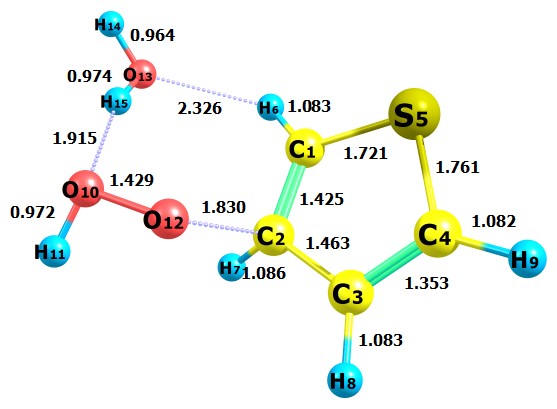


TS1-1WHB TS1-1WTA


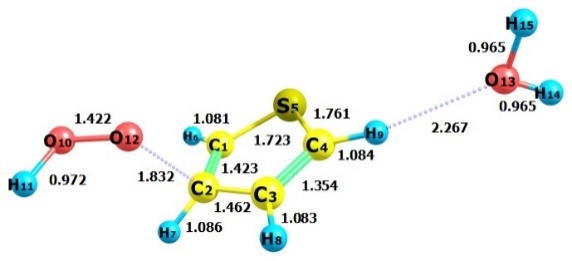

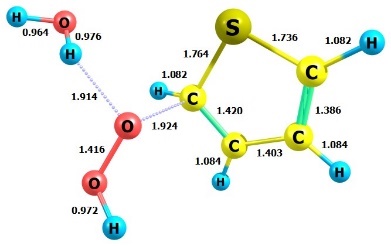


TS1-1WTA2 TS2-1WHB


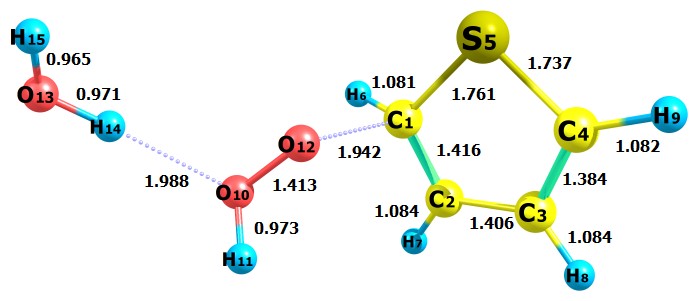

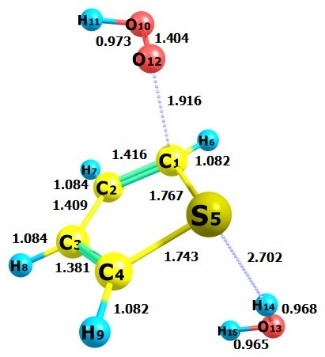


TS2-1WHC TS2-1WTD


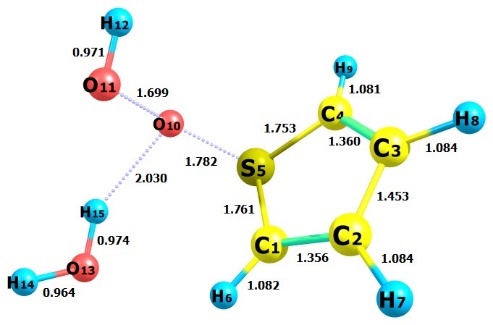

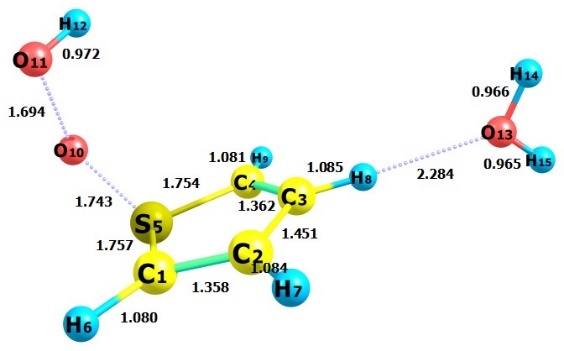


TS1-1WHB TS3-1WTB


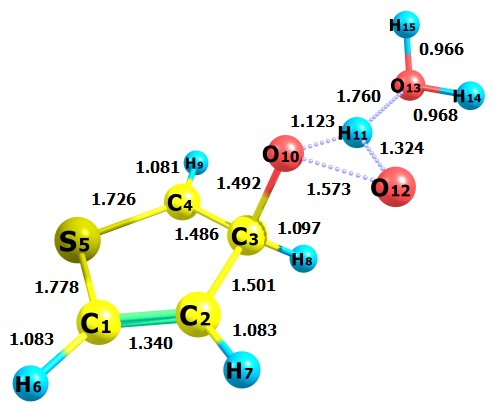

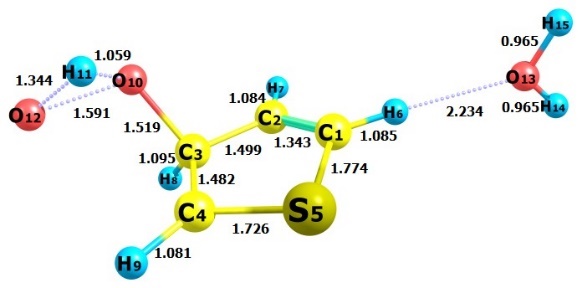


TS4-1WHA TS4-1WTA


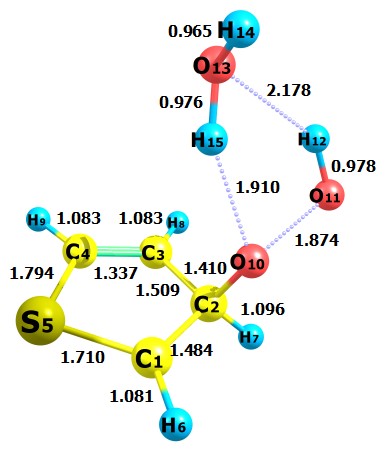

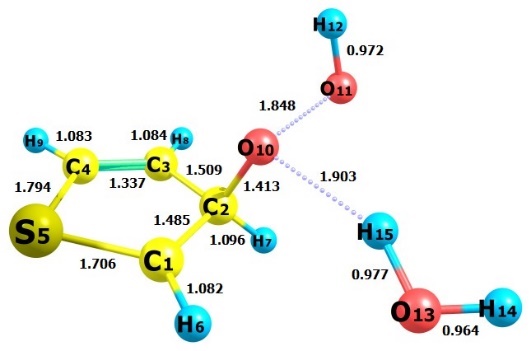


TS5-1WHA2 TS5-1WHB


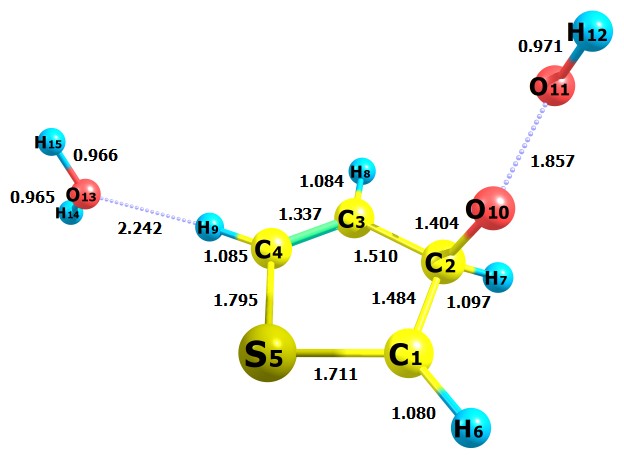

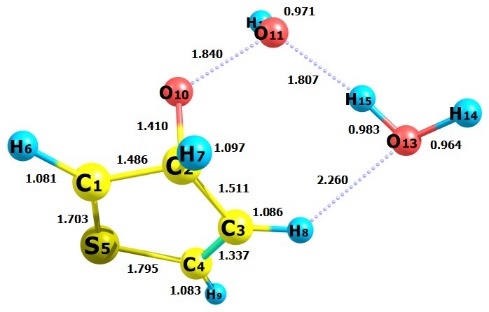


TS5-1WTA TS5-1WTB2


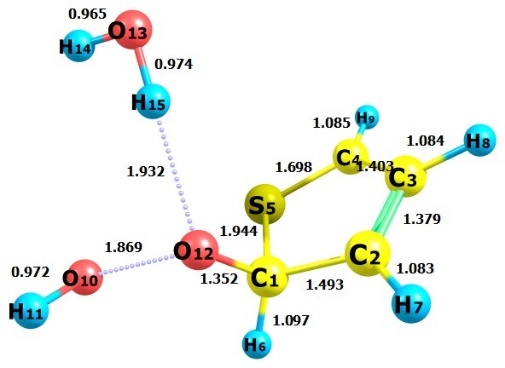

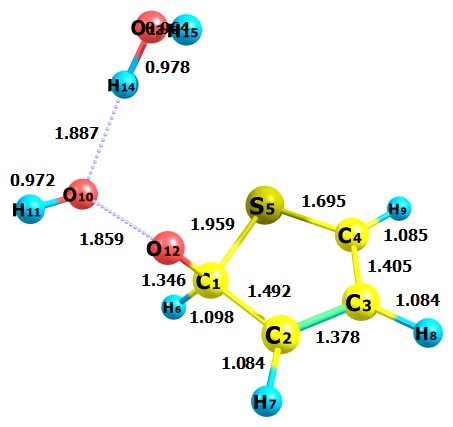


TS8a-1WHB TS8a-1WHC


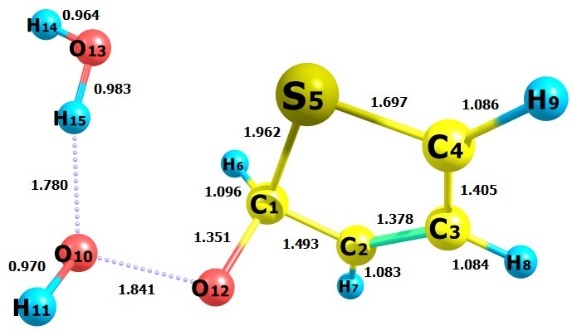

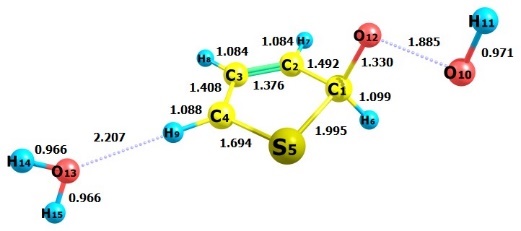


TS8a-1WHC2 TS8a-1WTA2


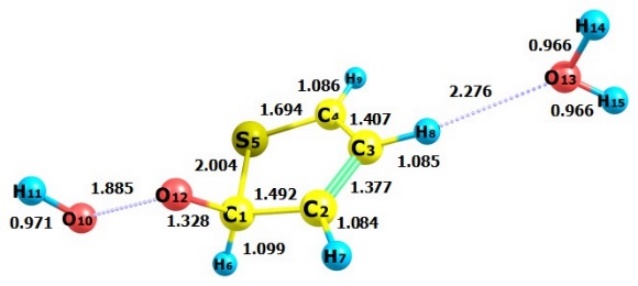

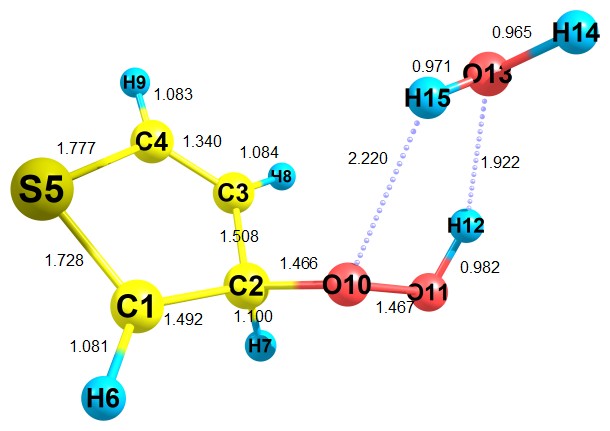


TS8a-1WTB CP-TS1-1WHA2


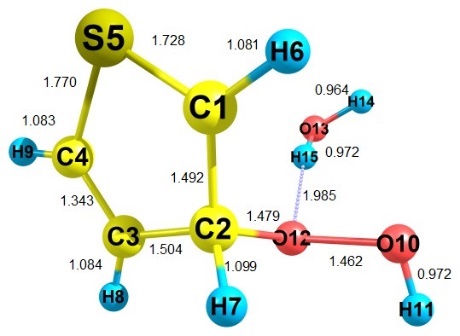

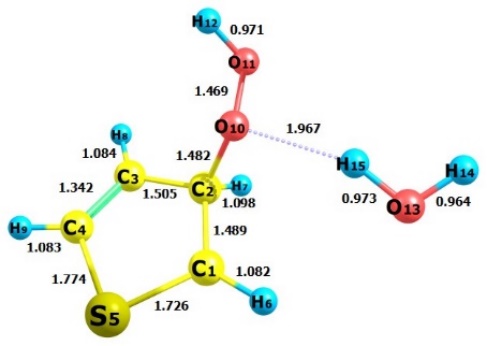


CP-TS1-1WHB CP-TS1-1WHB2


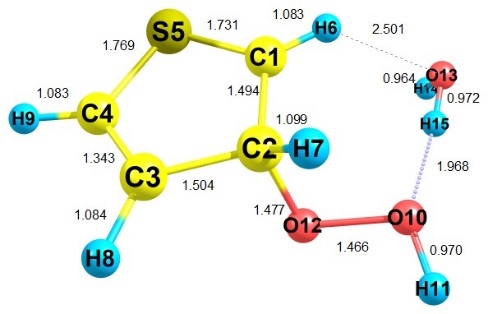

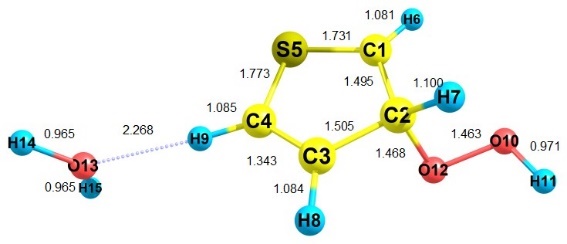


CP-TS1-1WTA2a CP-TS1-1WTA2b


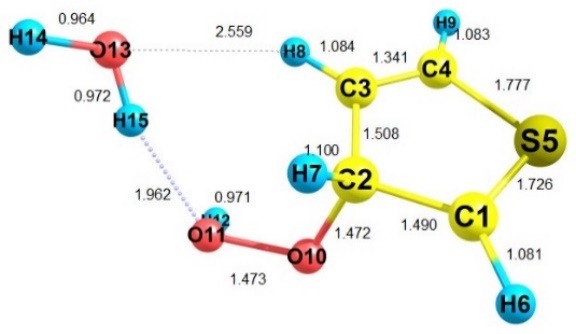

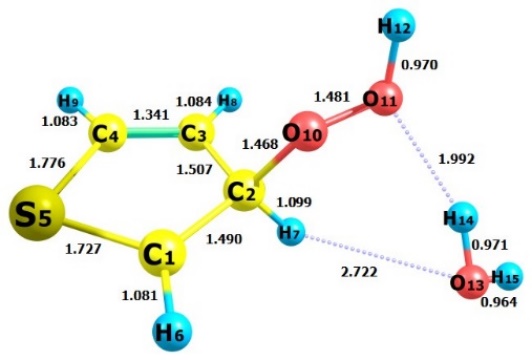


CP-TS1-1WTB2a CP-TS1-1WTB2b


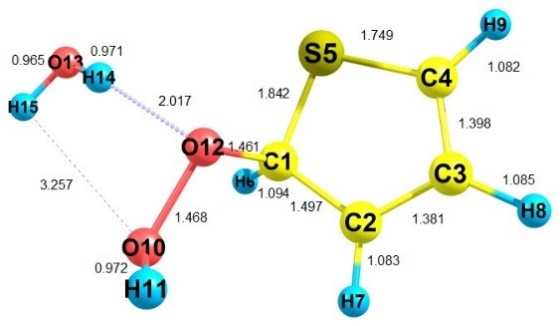

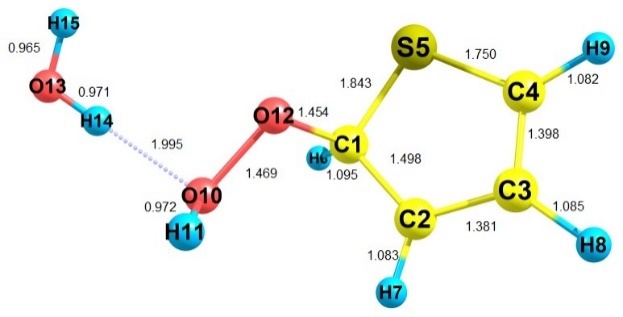


CP-TS2-1WHB CP-TS2-1WHC


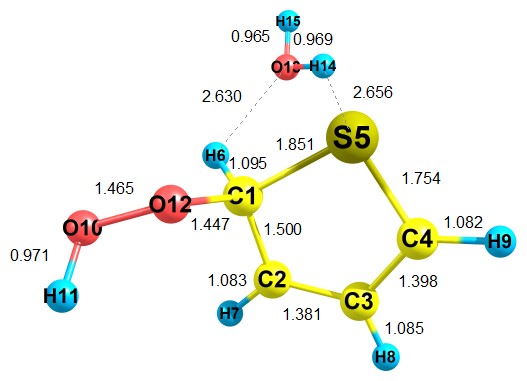

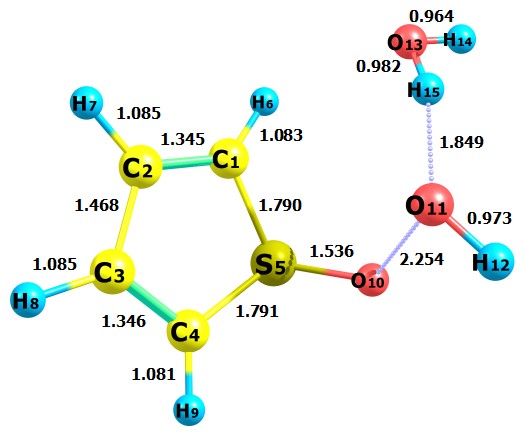


CP-TS2-1WTD CP-TS3-1WHB


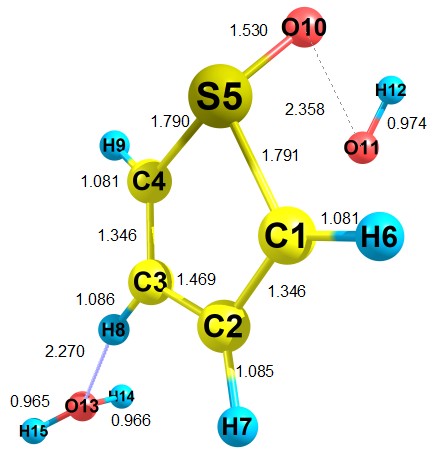

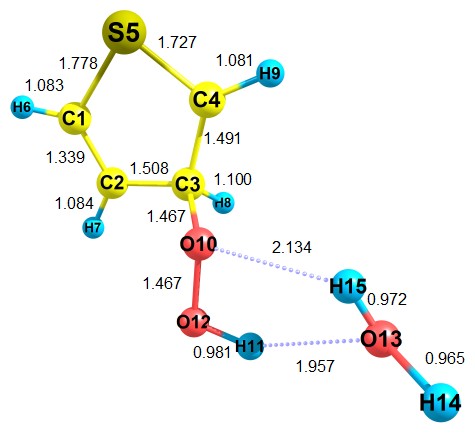


CP-TS3-1WTB CP-TS4-1WHA


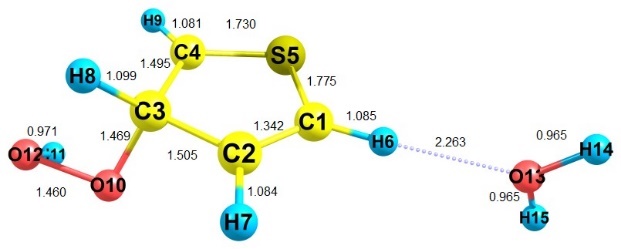

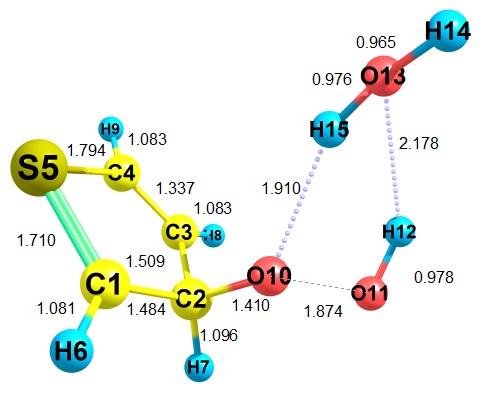


CP-TS4-1WTA CP-TS5-1WHA2


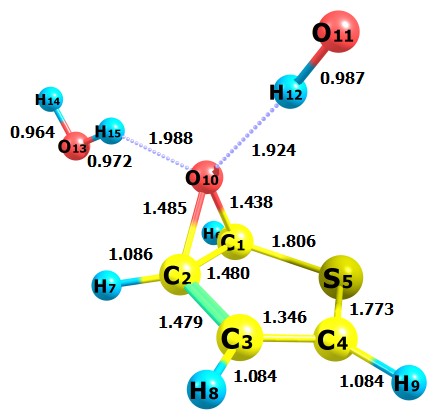

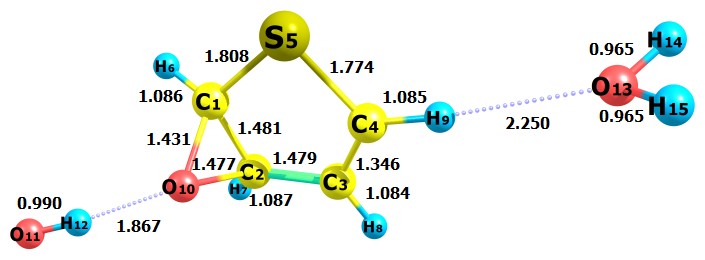


CP-TS5-1WHB CP-TS5-1WTA


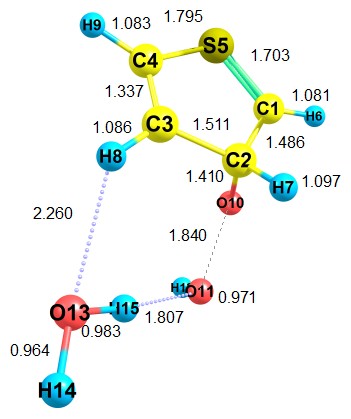

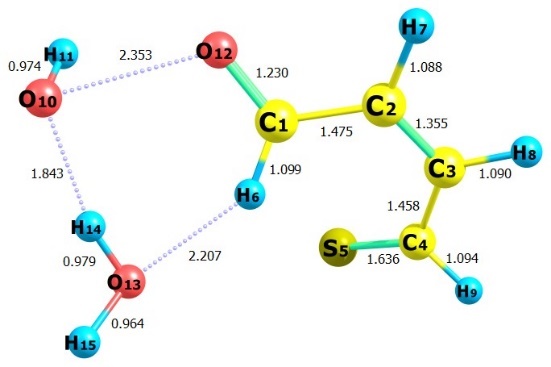


CP-TS5-1WTB2 CP-TS8a-1WHB


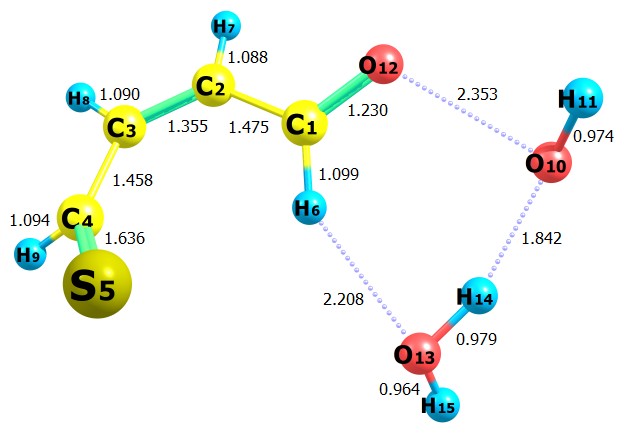

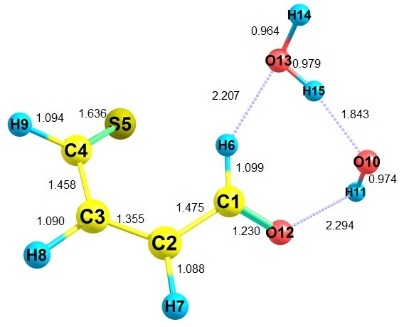


CP-TS8a-1WHC CP-TS8a-1WHC2


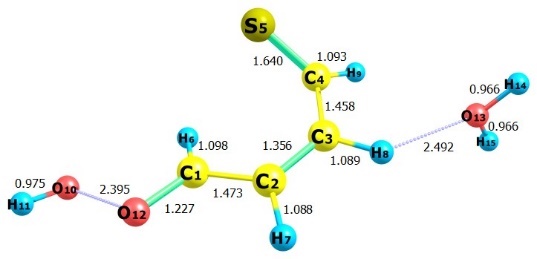

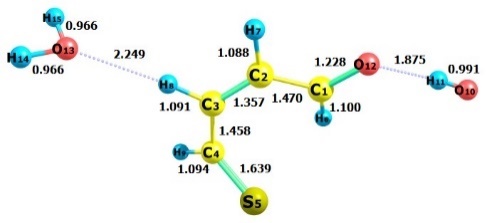


CP-TS8a-1WTA CP-TS8a-1WTB

**Table S1.** Calculated spin contamination values ⟨S^2^⟩ before and after spin annihilation at the B3LYP/6-31+G(d,p) level.

| Species | ⟨S^2^⟩^a^ | ⟨S^2^⟩_b_ |
| --- | --- | --- |
| H_2_O | 0.7538 | 0.7500 |
| 1WHA | 0.7538 | 0.7500 |
| CR-TS1-1WHA | 0.7542 | 0.7500 |
| CR-TS1-1WTB | 0.7550 | 0.7500 |
| CR-TS2-1WTA | 0.7539 | 0.7500 |
| CR-TS3-1WTA | 0.7554 | 0.7500 |
| CR-TS4-1WTB | 0.7540 | 0.7500 |
| TS1-1WHA | 0.7828 | 0.7503 |
| TS1-1WTB | 0.7766 | 0.7502 |
| TS2-1WHA | 0.7853 | 0.7508 |
| TS2-1WTA | 0.7785 | 0.7505 |
| TS3-1WTA | 0.7680 | 0.7501 |
| TS3-1WHA | 0.7693 | 0.7501 |
| TS4-1WTB | 0.7584 | 0.7502 |
| TS5-1WHA | 0.8163 | 0.7505 |
| TS5-1WTB | 0.7938 | 0.7503 |
| CP-TS1-1WAH2 | 0.7564 | 0.7500 |
| CP-TS1-1WTB2 | 0.7566 | 0.7501 |
| CP-TS3-1WTA | 0.7549 | 0.7501 |
| CP-TS3-1WHA | 0.7532 | 0.7500 |
| CP-TS5-1WHA | 0.7527 | 0.7500 |
| CP-TS5-1WTB | 0.7533 | 0.7500 |
| P1 | 0.7588 | 0.7500 |
| P2 | 0.7719 | 0.7501 |

a) Before spin annihilation b) After spin annihilation.

**Table S2.** Topological parameters of the stationary points in the presence of water for C_4_H_4_S + HO_2_ reaction computed at the B3LYP/6-31+ g(d,p) level.

| Species | Bond | ρ | Eigenvalues of the Hessian matrix | | | ∇^2^ ρ |
| --- | --- | --- | --- | --- | --- | --- |
|  |  |  | λ_1_ | λ_2_ | λ_3_ |  |
| 1WHA | H3-O4 | 0.0392 | 0.0630 | -0.0597 | 0.2378 | 0.1151 |
|  | O2-H3 | 0.3390 | -1.7995 | -1.7546 | 1.5699 | -1.9842 |
|  | O4-H6 | 0.3616 | -1.8555 | -1.8109 | 1.5697 | -2.0966 |
| CR-TS1-1WHA | C1-H11 | 0.0139 | -0.0144 | -0.0094 | 0.0597 | 0.0358 |
|  | O10-H14 | 0.0088 | -0.0088 | -0.0087 | 0.0457 | 0.0282 |
|  | O13-H7 | 0.0445 | 0.0445 | -0.0723 | 0.2771 | 0.1279 |
|  | RCP | 0.0030 | -0.0020 | 0.0056 | 0.0089 | 0.0125 |
| CR-TS1-1WTB | C1-H11 | 0.0178 | -0.0197 | -0.0091 | 0.0702 | 0.0415 |
|  | O10-H14 | 0.0144 | -0.0171 | -0.0160 | 0.0806 | 0.0476 |
|  | O13-H7 | 0.0093 | -0.0091 | -0.0086 | 0.0503 | 0.0325 |
|  | RCP | 0.0032 | -0.0021 | 0.0071 | 0.0095 | 0.0144 |
| CR-TS2-1WTA | H6-O13 | 0.0109 | -0.0111 | -0.0104 | 0.0587 | 0.0372 |
|  | H14-O10 | 0.0138 | -0.0162 | -0.0152 | 0.0781 | 0.0468 |
|  | H11-C1 | 0.0185 | -0.0205 | -0.0130 | 0.0749 | 0.0414 |
|  | RCP | 0.0036 | -0.0024 | 0.0075 | 0.0112 | 0.0163 |
| CR-TS3-1WTA | C3-H15 | 0.0135 | -0.0139 | -0.0067 | 0.0559 | 0.0353 |
|  | O13-H12 | 0.0442 | -0.0762 | -0.0717 | 0.2755 | 0.1276 |
|  | O10-H8 | 0.0071 | -0.0068 | -0.0067 | 0.0370 | 0.0236 |
|  | RCP | 0.0027 | -0.0018 | 0.0052 | 0.0077 | 0.0111 |
| CR-TS4-1WTB | H6-O13 | 0.0142 | -0.0154 | -0.0144 | 0.0768 | 0.0471 |
|  | H14-O12 | 0.0236 | -0.0321 | -0.0302 | 0.1271 | 0.0648 |
|  | H11-C1 | 0.0180 | -0.0201 | -0.0114 | 0.0705 | 0.0391 |
|  | RCP | 0.0026 | -0.0016 | 0.0063 | 0.0080 | 0.0126 |
|  | C2-O12 | 0.1058 | -0.1625 | -0.1462 | 0.4339 | 0.1252 |
| TS1-1WHA | O12-O10 | 0.3013 | -0.6894 | -0.6748 | 1.2634 | -0.1008 |
|  | H11-O13 | 0.0207 | -0.0253 | -0.0218 | 0.1123 | 0.0652 |
|  | H15-O12 | 0.0288 | -0.0385 | -0.0372 | 0.1599 | 0.0842 |
|  | RCP | 0.0155 | -0.0163 | 0.0266 | 0.0739 | 0.0842 |
|  | C2-O12 | 0.1012 | -0.1522 | -0.1376 | 0.4186 | 0.1288 |
| TS1-1WTB | O12-O10 | 0.2951 | -0.6669 | -0.6620 | 1.2631 | -0.0658 |
|  | H7-O13 | 0.0102 | -0.0101 | -0.0095 | 0.0535 | 0.0339 |
|  | H14-O10 | 0.0262 | -0.0359 | -0.0341 | 0.1485 | 0.0785 |
|  | RCP | 0.0059 | -0.0048 | 0.0121 | 0.0232 | 0.0306 |
| TS2-1WHA | C1-O12 | 0.0886 | -0.1306 | -0.1136 | 0.3873 | 0.1431 |
|  | O10-O12 | 0.3117 | -0.7179 | -0.7007 | 1.2955 | -0.1231 |
|  | H11-O13 | 0.0254 | -0.0330 | -0.0308 | 0.1389 | 0.0751 |
|  | H15-O12 | 0.0233 | -0.0290 | -0.0273 | 0.1263 | 0.0700 |
|  | RCP | 0.0154 | -0.0160 | 0.0304 | 0.0687 | 0.0831 |

| TS2-1WTA | C1-O12 | 0.0827 | | -0.1189 | -0.1029 | 0.3640 | 0.1423 | |
| --- | --- | --- | --- | --- | --- | --- | --- | --- |
|  | O10-O12 | 0.3044 | | -0.6923 | -0.6870 | 1.2884 | -0.0909 | |
|  | H14-O10 | 0.0253 | | -0.0345 | -0.0327 | 0.1440 | 0.0767 | |
|  | H6-O13 | 0.0124 | | -0.0130 | -0.0125 | 0.0651 | 0.0396 | |
|  | RCP | 0.0059 | | -0.0045 | 0.0139 | 0.0216 | 0.0310 | |
| TS3-1WTA | S5-O10 | 0.1585 | | -0.2315 | -0.2206 | 0.4648 | 0.0126 | |
|  | O10-O11 | 0.1437 | | -0.2767 | -0.2668 | 0.9439 | 0.4003 | |
|  | O11-H14 | 0.0305 | | -0.0433 | -0.0426 | 0.1791 | 0.0932 | |
|  | O13-H6 | 0.0145 | | -0.0151 | -0.0148 | 0.0776 | 0.0478 | |
|  | RCP | 0.0040 | | -0.0017 | 0.0073 | 0.0128 | 0.0184 | |
| TS3-1WHA | S5-O10 | 0.1684 | | -0.2421 | -0.2387 | 0.4398 | -0.0410 | |
|  | O10-O11 | 0.1366 | | -0.2593 | -0.2486 | 0.9135 | 0.4056 | |
|  | O10-H15 | 0.0287 | | -0.0390 | -0.0372 | 0.1589 | 0.0827 | |
|  | O13-H9 | 0.0054 | | -0.0041 | -0.0032 | 0.0285 | 0.0211 | |
|  | RCP | 0.0044 | | -0.0022 | 0.0044 | 0.0173 | 0.0195 | |
| TS4-1WTB | C3-O10 | 0.1979 | | -0.3483 | -0.3328 | 0.4172 | -0.2639 | |
|  | O10-O12 | 0.1772 | | -0.3392 | -0.2151 | 0.9376 | 0.3833 | |
|  | O10-H11 | 0.2568 | | -1.1371 | -1.0364 | 1.0867 | -1.0868 | |
|  | O12-H14 | 0.0313 | | -0.0458 | -0.0424 | 0.1812 | 0.0930 | |
|  | O13-H7 | 0.0138 | | -0.0149 | -0.0140 | 0.0725 | 0.0436 | |
|  | RCP | 0.0040 | | -0.0024 | 0.0088 | 0.0139 | 0.0203 | |
|  | C2-O10 | 0.2699 | | -0.5193 | -0.4938 | 0.4297 | -0.5834 | |
|  | O11-O10 | 0.087808 | | -0.14857 | -0.1334 | 0.623062 | 0.3411 | |
| TS5-1WHA | H12-O13 | 0.015915 | | -0.01822 | -0.01536 | 0.083469 | 0.0499 | |
|  | H4-O10 | 0.024008 | | -0.03069 | -0.02931 | 0.134738 | 0.0747 | |
|  | RCP | 0.011306 | | -0.01109 | 0.018834 | 0.049124 | 0.0569 | |
|  | C2-O10 | 0.266346 | | -0.5081 | -0.48284 | 0.41639 | -0.5745 | |
|  | O11-O10 | 0.098191 | | -0.17368 | -0.15483 | 0.684835 | 0.3563 | |
| TS5-1WTB | H14-O11 | 0.032839 | | -0.0473 | -0.04632 | 0.19 | 0.0964 | |
|  | H7-O13 | 0.011505 | | -0.01152 | -0.01073 | 0.060394 | 0.0381 | |
|  | RCP | 0.006555 | | -0.00397 | 0.012627 | 0.028408 | 0.0371 | |
|  | C2-O10 | 0.2388 | | -0.4599 | -0.4286 | 0.3847 | -0.5037 | |
| CP-TS1-1WAH2 | O11-O10 | 0.2688 | | -0.6170 | -0.5811 | 1.1701 | -0.0280 | |
|  | H12-O13 | 0.0264 | | -0.0349 | -0.0339 | 0.1470 | 0.0782 | |
|  | H14-O10 | 0.0166 | | -0.0190 | -0.0156 | 0.0914 | 0.0568 | |
|  | RCP | 0.0133 | | -0.0130 | 0.0216 | 0.0580 | 0.0666 | |
|  | C2-O10 | 0.2335 | | -0.4380 | -0.4093 | 0.3831 | -0.4642 | |
| CP-TS1-1WTB2 | O11-O10 | 0.2595 | | -0.5999 | -0.5517 | 1.1574 | 0.0059 | |
|  | H14-O11 | 0.0219 | | -0.0288 | -0.0267 | 0.1217 | 0.0663 | |
|  | H7-O13 | 0.0058 | | -0.0050 | -0.0042 | 0.0302 | 0.0210 | |
|  | RCP | 0.0049 | | -0.0031 | 0.0066 | 0.0212 | 0.0247 | |
|  | C2-O12 | 0.2339 | | -0.4434 | -0.4189 | 0.3865 | -0.4758 | |
|  | O12-O10 | 0.2727 | | -0.6252 | -0.5945 | 1.1757 | -0.0439 | |
| CP-TS1-1WAH1 | H11-O13 | 0.0257 | | -0.0336 | -0.0321 | 0.1412 | 0.0754 | |
|  | H15-O12 | 0.0182 | | -0.0214 | -0.0184 | 0.1005 | 0.0608 | |
|  | RCP | 0.0138 | | -0.0135 | 0.0243 | 0.0599 | 0.0707 | |
|  | C2-O12 | 0.2273 | | -0.4302 | -0.4029 | 0.3949 | -0.4382 | |
| CP-TS1-1WTB1 | O12-O10 | 0.2679 | | -0.6056 | -0.5884 | 1.1835 | -0.0105 | |
|  | H14-O10 | 0.0248 | | -0.0333 | -0.0314 | 0.1405 | 0.0758 | |
|  | H7-O13 | 0.0086 | | -0.0081 | -0.0069 | 0.0446 | 0.0295 | |
|  | RCP | 0.0062 | | -0.0054 | 0.0101 | 0.0260 | 0.0306 | |
| CP-TS3-1WTA | S5-O10 | | 0.2467 | -0.3532 | -0.3329 | 1.0753 | 0.3892 |  |
|  | H12-O11 | | 0.0406 | -0.0645 | -0.0625 | 0.2524 | 0.1253 |  |
|  | O11-H14 | | 0.0309 | -0.0436 | -0.0433 | 0.1727 | 0.0858 |  |
|  | O13-H6 | | 0.0163 | -0.0188 | -0.0172 | 0.0875 | 0.0515 |  |
|  | RCP | | 0.0018 | -0.0008 | 0.0038 | 0.0056 | 0.0087 |  |
| CP-TS3-1WHA | S5-O10 | | 0.2482 | -0.3507 | -0.3372 | 1.0849 | 0.3970 |  |
|  | O10-H15 | | 0.0369 | -0.0566 | -0.0533 | 0.2171 | 0.1072 |  |
|  | O13-H12 | | 0.0334 | -0.0497 | -0.0462 | 0.1899 | 0.0941 |  |
|  | O11-C2 | | 0.0155 | -0.0129 | -0.0034 | 0.0706 | 0.0544 |  |
|  | RCP | | 0.0033 | -0.0020 | 0.0023 | 0.0128 | 0.0131 |  |
| CP-TS5-1WHA | H14-O10 | | 0.0263 | -0.0358 | -0.0344 | 0.1476 | 0.0774 |  |
|  | O13-H12 | | 0.0314 | -0.0454 | -0.0428 | 0.1768 | 0.0886 |  |
|  | O11…C3=C4 | | 0.0135 | -0.0108 | -0.0014 | 0.0584 | 0.0461 |  |
|  | RCP_C1-O10-C2_ | | 0.2023 | -0.3709 | 0.1951 | 0.4928 | 0.3171 |  |
|  | RCP | | 0.0033 | -0.0014 | 0.0027 | 0.0116 | 0.0129 |  |
| CP-TS5-1WTB | H12-O10 | | 0.0348 | -0.0523 | -0.0507 | 0.2031 | 0.1000 |  |
|  | O11-H14 | | 0.0270 | -0.0363 | -0.0361 | 0.1473 | 0.0749 |  |
|  | O13-H7 | | 0.0132 | -0.0145 | -0.0131 | 0.0706 | 0.0430 |  |
|  | RCP_C1-O10-C2_ | | 0.2003 | -0.3671 | 0.1832 | 0.5011 | 0.3172 |  |
|  | RCP | | 0.0030 | 0.0030 | 0.0062 | 0.0111 | 0.0152 |  |
| CP-TS2-1WHA | C1-O12 | | 0.2504 | -0.5041 | -0.4553 | 0.3842 | -0.5752 |  |
|  | O10-O12 | | 0.2695 | -0.6160 | -0.5851 | 1.1711 | -0.0300 |  |
|  | H11-O13 | | 0.0295 | -0.0411 | -0.0394 | 0.1658 | 0.0852 |  |
|  | H15-O12 | | 0.0127 | -0.0134 | -0.0081 | 0.0703 | 0.0487 |  |
|  | RCP | | 0.0120 | -0.0114 | 0.0112 | 0.0563 | 0.0561 |  |
| CP-TS2-1WTA | C1-O12 | | 0.2414 | -0.4774 | -0.4332 | 0.3828 | -0.5278 |  |
|  | O10-O12 | | 0.2696 | -0.6113 | -0.5935 | 1.1834 | -0.0215 |  |
|  | H6-O13 | | 0.0088 | -0.0085 | -0.0073 | 0.0460 | 0.0302 |  |
|  | H14-O10 | | 0.0223 | -0.0292 | -0.0274 | 0.1244 | 0.0679 |  |
|  | RCP | | 0.0063 | -0.0056 | 0.0106 | 0.0256 | 0.0307 |  |
| CP-TS4-1WTB | C3-O10 | | 0.2324 | -0.4361 | -0.4118 | 0.3841 | -0.4639 |  |
|  | O10-O12 | | 0.2641 | -0.6081 | -0.5684 | 1.1689 | -0.0076 |  |
|  | O12-H14 | | 0.0230 | -0.0307 | -0.0285 | 0.1297 | 0.0705 |  |
|  | O13-H7 | | 0.0082 | -0.0078 | -0.0070 | 0.0424 | 0.0276 |  |
|  | RCP | | 0.0038 | -0.0025 | 0.0069 | 0.0136 | 0.0181 |  |

**Table S3.** The thermodynamic parameters (kcal mol^-1^) at 298 K for all stationary points of the water-assisted reaction calculated at the B3LYP method.

| Species | ΔE^0^ | ΔH^0^ | ΔG^0^ | -TΔS^0^ |
| --- | --- | --- | --- | --- |
| C_4_H_4_S + HO_2_ + H_2_O | 0.00 | 0.00 | 0.00 | 0.00 |
| Thiophene + 1WHA | -7.51 | -8.10 | 0.09 | -8.20 |
| HO_2_ + 1WTA | 4.20 | 3.61 | 11.30 | -7.69 |
| HO_2_ + 1WTB | 4.67 | 4.08 | 11.49 | -7.41 |
| CR-TS1-1WHA | -10.06 | -11.24 | 5.22 | -16.46 |
| CR-TS1-1WTB | -4.42 | -5.60 | 9.54 | -15.14 |
| CR-TS2-1WTA | -4.78 | -5.97 | 9.36 | -15.33 |
| CR-TS3-1WHA | -9.31 | -10.49 | 5.67 | -16.16 |
| CR-TS4-1WHA | -9.25 | -10.43 | 4.82 | -15.25 |
| CR-TS4-1WTB | -6.75 | -7.94 | 8.45 | -16.39 |
| TS1-1WHA | 11.15 | 9.97 | 30.30 | -20.33 |
| TS1-1WTB | 13.23 | 12.05 | 31.49 | -19.44 |
| TS2-1WHA | -0.56 | 4.51 | 24.80 | -20.30 |
| TS2-1WTA | 7.97 | 6.78 | 26.46 | -19.68 |
| TS3-1WTA | 26.75 | 25.57 | 45.35 | -19.78 |
| TS3-1WHA | 28.81 | 27.63 | 46.50 | -18.87 |
| TS4-1WHA | 51.76 | 50.58 | 70.55 | -19.97 |
| TS4-1WTB | 50.83 | 49.65 | 69.67 | -20.02 |
| TS5-1WHA | 18.64 | 17.46 | 36.86 | -19.40 |
| TS5-1WTB | 15.63 | 14.44 | 34.14 | -19.69 |
| TS8a-1WHA | 0.42 | 5.83 | 25.20 | -19.37 |
| TS8a-1WTA | 4.24 | 3.05 | 22.59 | -19.54 |
| IN8a-1WHA | -7.95 | -9.14 | 11.68 | -20.82 |
| IN8a-1WTA | -3.92 | -5.11 | 13.11 | -18.21 |
| CP-TS1-1WAH1 | 6.38 | 5.20 | 24.95 | -19.76 |
| CP-TS1-1WTB1 | 8.13 | 6.94 | 26.18 | -19.24 |
| CP-TS1-1WAH2 | 6.01 | 4.83 | 55.25 | -19.75 |
| CP-TS1-1WTB2 | 8.50 | 7.32 | 25.78 | -18.46 |
| CP-TS2-1WHA | -4.73 | -5.91 | 13.93 | -19.84 |
| CP-TS2-1WTA | -1.70 | -2.88 | 16.32 | -19.20 |
| CP-TS3-1WTA | 4.63 | 3.45 | 21.93 | -18.48 |
| CP-TS3-1WHA | 4.82 | 3.63 | 22.66 | -19.03 |
| CP-TS4-1WHA | 6.00 | 4.81 | 24.62 | -19.80 |
| CP-TS4-1WTB | 7.96 | 6.78 | 25.71 | -18.94 |
| CP-TS5-1WHA | -9.14 | -10.32 | 9.39 | -19.71 |
| CP-TS5-1WTB | -8.11 | -9.30 | 9.59 | -18.89 |
| CP-TS8a-1WHA | -17.53 | -18.72 | -2.39 | -16.33 |
| CP-TS8a-1WTA | -14.22 | -15.41 | 0.76 | -16.17 |
| P1+H_2_O | 10.97 | 10.38 | 21.36 | -10.98 |
| P2+H_2_O | -6.28 | -6.88 | 1.97 | -8.85 |
| P3+H_2_O | 19.19 | 19.19 | 20.94 | -1.75 |
| P4+H_2_O | -6.48 | -6.48 | -6.84 | 0.36 |
| P5+H_2_O | 1.94 | 1.94 | 4.44 | -2.50 |

**Table S4.** The thermodynamic parameters (kcal mol^-1^) at 298 K for all stationary points of the water-assisted reaction calculated at the CCSD(T) method.

| Species | ΔE^0^ | ΔH^0^ | ΔG^0^ | -TΔS^0^ |
| --- | --- | --- | --- | --- |
| C_4_H_4_S + HO_2_ + H_2_O | 0.00 | 0.00 | 0.00 | 0.00 |
| Thiophene + 1WHA | -7.51 | -8.11 | 0.09 | -8.20 |
| HO_2_ + 1WTA | 3.19 | 2.59 | 10.29 | -7.69 |
| HO_2_ + 1WTB | 3.73 | 3.13 | 10.54 | -7.41 |
| CR-TS1-1WHA | -11.57 | -12.76 | 3.70 | -16.46 |
| CR-TS1-1WTB | -6.90 | -8.08 | 7.06 | -15.14 |
| CR-TS2-1WTA | -7.40 | -8.58 | 6.75 | -15.33 |
| CR-TS3-1WHA | -10.46 | -11.64 | 4.52 | -16.16 |
| CR-TS4-1WHA | -10.96 | -12.15 | 3.11 | -15.25 |
| CR-TS4-1WTB | -8.66 | -9.85 | 6.54 | -16.39 |
| TS1-1WHA | 9.98 | 8.79 | 29.12 | -20.33 |
| TS1-1WTB | 12.06 | 10.88 | 30.32 | -19.44 |
| TS2-1WHA | -0.65 | 4.42 | 24.71 | -20.30 |
| TS2-1WTA | 8.00 | 6.82 | 26.50 | -19.68 |
| TS3-1WTA | 26.85 | 25.66 | 45.44 | -19.78 |
| TS3-1WHA | 27.21 | 26.03 | 44.90 | -18.87 |
| TS4-1WHA | 47.07 | 45.89 | 65.86 | -19.97 |
| TS4-1WTB | 46.72 | 45.53 | 65.55 | -20.02 |
| TS5-1WHA | 15.33 | 14.15 | 33.55 | -19.40 |
| TS5-1WTB | 14.27 | 13.09 | 32.78 | -19.69 |
| TS8a-1WHA | 1.44 | 6.85 | 26.22 | -19.37 |
| TS8a-1WTA | 6.51 | 5.33 | 24.87 | -19.54 |
| IN8a-1WHA | -10.94 | -12.12 | 8.70 | -20.82 |
| IN8a-1WTA | -7.58 | -8.76 | 9.45 | -18.21 |
| CP-TS1-1WAH1 | 1.38 | 0.20 | 19.96 | -19.76 |
| CP-TS1-1WTB1 | 3.20 | 2.02 | 21.26 | -19.24 |
| CP-TS1-1WAH2 | 1.34 | 0.15 | 50.57 | -50.42 |
| CP-TS1-1WTB2 | 3.58 | 2.40 | 20.86 | -18.46 |
| CP-TS4-1WHA | 1.15 | -0.03 | 19.77 | -19.81 |
| CP-TS4-1WTB | 3.32 | 2.13 | 21.07 | -18.94 |
| CP-TS2-1WHA | -9.02 | -10.20 | 9.64 | -19.84 |
| CP-TS2-1WTA | -5.69 | -6.87 | 12.33 | -19.20 |
| CP-TS3-1WTA | -1.58 | -2.76 | 15.72 | -18.48 |
| CP-TS3-1WHA | -1.69 | -2.88 | 16.15 | -19.03 |
| CP-TS5-1WHA | -17.87 | -19.05 | 0.66 | -19.71 |
| CP-TS5-1WTB | -16.93 | -18.11 | 0.77 | -18.89 |
| CP-TS8a-1WHA | -20.35 | -21.53 | -5.20 | -16.33 |
| CP-TS8a-1WTA | -14.73 | -15.91 | 0.26 | -16.17 |
| P1+H_2_O | 7.31 | 6.72 | 17.70 | -10.98 |
| P2+H_2_O | -8.35 | -8.95 | -0.10 | -8.85 |
| P3+H_2_O | 14.51 | 14.51 | 16.26 | -1.75 |
| P4+H_2_O | -10.83 | -10.83 | -11.19 | 0.36 |
| P5+H_2_O | -5.06 | -5.06 | -2.56 | -2.50 |

**Table S5.** Total energies (in Hartree) of the reactants, products, intermediates, and transition states involved in the bare reaction at different levels of theory.

| Stationary Points | | B3LYP^a^ | CBS-QB3 | | CCSD (T)^b^ | | BD(T)^c^ |  |
| --- | --- | --- | --- | --- | --- | --- | --- | --- |
| C_4_H_4_S | | -553.0180 | | -552.2679 | -552.0330 | | -552.0328 | |
| HO_2_ | | -150.9154 | | -150.7411 | -150.5494 | | -150.5492 | |
| CR1 | | -703.9405 | | -703.0167 | -702.5914 | | -702.5914 | |
| CR3 | | -703.9393 | | -703.0159 | -702.5900 | | -702.5896 | |
| TS1 | | -703.9077 | | -702.9856 | -702.5564 | | -702.5571 | |
| TS2 | | -703.9158 | | -702.9913 | -702.5627 | | -702.5636 | |
| TS3 | | -703.8798 | | -702.9628 | -702.5262 | | -702.5284 | |
| TS4 | | -703.8398 | | -702.9219 | -702.4948 | | -702.4941 | |
| TS5 | | -703.8987 | | -702.9753 | -702.5499 | | -702.5515 | |
| TS8a | | -703.9167 | | -702.9817 | -702.5562 | | -702.5600 | |
| CP3 | | -703.9105 | | -703.0098 | -702.5664 | | -702.5659 | |
| CP5 | | -703.9386 | | -703.0192 | -702.6007 | | -702.6003 | |
| CP8a | | -703.9490 | | -703.0229 | -702.5995 | | -702.6033 | |
| OH | | -75.7390 | | -75.6497 | -75.5576 | | -75.5577 | |
| P1 | | -703.9188 | | -702.9988 | -702.5737 | | -702.5736 | |
| P2 | | -703.9394 | | -703.0134 | -702.5917 | | -702.5886 | |
| C_4_H_4_SO | | -628.1618 | | -627.3494 | -626.9997 | | -626.9993 | |
| SC_4_H_4_O | | -628.2033 | | -627.3663 | -627.0407 | | -627.0401 | |
| SCHOCHC_2_H_2_ | | -628.1902 | | -627.3630 | -627.0318 | | -627.0314 | |

^a^B3LYP method in conjunction with 6-31+g(d,p) basis set. ^b^CCSD(T) single point on the optimized structure of the B3LYP/6-31+g(d,p) level. ^c^BD(T)/6-31+g(d,p)//B3LYP/6-31+g(d,p) level.

**Table S6.** The calculated zero point and thermodynamic (298.15 K) corrections of the reactants, products, intermediates, and transition states involved in the bare reaction using the B3LYP/6-31+g(d,p) level of theory.

| Stationary Points | | ZPE^a^ | E^0a^ | | H^0a^ | G^0a^ | S^0b^ |  |
| --- | --- | --- | --- | --- | --- | --- | --- | --- |
| C_4_H_4_S | | 0.0666 | | 0.070723 | 0.071667 | | 0.039394 | 67.924 |
| HO_2_ | | 0.0141 | | 0.016963 | 0.017907 | | -0.00809 | 54.704 |
| CR1 | | 0.0823 | | 0.090752 | 0.091697 | | 0.04501 | 98.26 |
| CR3 | | 0.0822 | | 0.090664 | 0.091608 | | 0.044952 | 98.195 |
| TS1 | | 0.0822 | | 0.089227 | 0.090171 | | 0.049509 | 85.58 |
| TS2 | | 0.0822 | | 0.089253 | 0.090197 | | 0.049508 | 85.638 |
| TS3 | | 0.0802 | | 0.087728 | 0.088673 | | 0.046841 | 88.042 |
| TS4 | | 0.0792 | | 0.086179 | 0.087123 | | 0.046818 | 84.829 |
| TS5 | | 0.0806 | | 0.088094 | 0.089038 | | 0.047083 | 88.302 |
| TS8a | | 0.0806 | | 0.088139 | 0.089083 | | 0.047341 | 87.854 |
| CP3 | | 0.0800 | | 0.087699 | 0.088643 | | 0.046921 | 87.813 |
| CP5 | | 0.0820 | | 0.088607 | 0.089551 | | 0.049357 | 84.596 |
| CP8a | | 0.0796 | | 0.089202 | 0.090146 | | 0.042149 | 101.018 |
| OH | | 0.0084 | | 0.010804 | 0.011748 | | -0.00849 | 42.599 |
| P1 | | 0.0832 | | 0.090545 | 0.091489 | | 0.050727 | 85.791 |
| P2 | | 0.0837 | | 0.090799 | 0.091743 | | 0.051485 | 84.731 |
| C_4_H_4_SO | | 0.0695 | | 0.074829 | 0.075773 | | 0.040533 | 74.168 |
| SC_4_H_4_O | | 0.0691 | | 0.075438 | 0.076382 | | 0.037787 | 81.231 |
| SCHOCHC_2_H_2_ | | 0.0710 | | 0.075745 | 0.076689 | | 0.042648 | 71.646 |

^a^ in Hatree and ^b^in cal mol^-1^.

**Table S7.** The total energies for all components of the title reaction in the main water-assisted pathways using the B3LYP, CBS-QB3, CCSD(T), and BD(T) methods (unit of all numbers is Hartree).

| Species | B3LYP^a^ | CBS-QB3 | CCSD (T)^b^ | BD(T)^c^ |
| --- | --- | --- | --- | --- |
| Thio | -553.0180 | -552.2679 | -552.0330 | -552.0328 |
| HO_2_ | -150.9154 | -150.7411 | -150.5494 | -150.5492 |
| H_2_O | -76.4340 | -76.3375 | -76.2444 | -76.2443 |
| 1WHA | -227.3654 | -227.0884 | -226.8097 | -226.8094 |
| 1WHB | -227.3548 | - | -226.7995 | -226.7992 |
| 1WHC | -227.3536 | - | -226.7987 | -226.7984 |
| 1WTA | -629.4556 | - | -628.2826 | -628.2823 |
| 1WTB | -629.4549 | - | -628.2817 | -628.2814 |
| 1WTC | -629.4557 | - | -628.2829 | -628.2825 |
| IWTD | -629.4549 | - | -628.2818 | -628.2815 |
| CR-TS1-1WHA | -780.3904 | -779.3646 | -778.8522 | -778.8521 |
| CR-TS1-1WTB | -780.3809 | -779.3630 | -778.8442 | -778.8441 |
| CR-TS2-1WTA | -780.3815 | - | -778.8450 | -778.8449 |
| CR-TS3-1WHA | -780.3892 | -779.3651 | -778.8504 | -778.8506 |
| CR-TS4-1WHA | -780.3889 | -779.3665 | -778.8513 | -778.8507 |
| CR-TS4-1WTB | -780.3848 | -779.3606 | -778.8472 | -778.8471 |
| TS1-1WHA | -780.3551 | -779.3325 | -778.8163 | -778.8168 |
| TS1-1WTB | -780.3515 | -779.3292 | -778.8127 | -778.8133 |
| TS2-1WHA | -780.3638 | -779.3354 | -778.8233 | -778.8240 |
| TS2-1WTA | -780.3599 | -779.3398 | -778.8192 | -778.8199 |
| TS3-1WTA | -780.3286 | -779.3063 | -778.7879 | -778.7897 |
| TS3-1WHA | -780.3253 | -779.3087 | -778.7872 | -778.7876 |
| TS4-1WHA | -780.2860 | -779.2695 | -778.7528 | -778.7521 |
| TS4-1WTB | -780.2880 | -779.2693 | -778.7539 | -778.7535 |
| TS5-1WHA | -780.3419 | -779.3197 | -778.8065 | -778.8082 |
| TS5-1WTB | -780.3468 | -779.3209 | -778.8083 | -778.8103 |
| TS8a-1WHA | -780.3606 | -779.3279 | -778.8183 | -778.8186 |
| TS8a-1WTA | -780.3651 | -779.3298 | -778.8208 | -778.8213 |
| IN8a-1WHA | -780.3871 | -779.3645 | -778.8512 | -778.8512 |
| IN8a-1WTA | -780.3801 | -779.3585 | -778.8453 | -778.8453 |
| CP-TS1-1WAH1 | -780.3640 | -779.3439 | -778.8313 | -778.8311 |
| CP-TS1-1WTB1 | -780.3609 | -779.3408 | -778.8281 | -778.8279 |
| CP-TS1-1WAH2 | -780.3645 | -779.3451 | -778.8313 | -778.8321 |
| CP-TS1-1WTB2 | -780.3602 | -779.3406 | -778.8274 | -778.8272 |
| CP-TS2-1WHA | -780.3818 | -779.3607 | -778.8480 | -778.8480 |
| CP-TS2-1WTA | -780.3766 | -779.3559 | -778.8423 | -778.8423 |
| CP-TS3-1WTA | -780.3644 | -779.3566 | -778.8337 | -778.8331 |
| CP-TS3-1WHA | -780.3647 | -779.3585 | -778.8344 | -778.8338 |
| CP-TS4-1WHA | -780.3646 | -779.3446 | -778.8317 | -778.8315 |
| CP-TS4-1WTB | -780.3611 | -779.3413 | -778.8278 | -778.8276 |
| CP-TS5-1WHA | -780.3879 | -779.3684 | -778.8612 | -778.8608 |
| CP-TS5-1WTB | -780.3857 | -779.3671 | -778.8591 | -778.8587 |
| CP-TS8a-1WHA | -780.4008 | -779.3700 | -778.8647 | -778.8678 |
| CP-TS8a-1WTA | -780.3955 | -779.3696 | -778.8557 | -778.8593 |
| OH | -75.7390 | -75.6497 | -75.5576 | -75.5577 |
| P1 | -703.9188 | -702.9988 | -702.5737 | -702.5736 |
| P2 | -703.9394 | -703.0134 | -702.5917 | -702.5886 |
| C_4_H_4_SO | -628.1618 | -627.3494 | -626.9997 | -626.9993 |
| SC_4_H_4_O | -628.2033 | -627.3663 | -627.0407 | -627.0401 |
| SCHOCHC_2_H_2_ | -628.1902 | -627.3630 | -627.0318 | -627.0314 |

^a^B3LYP method in conjunction with 6-31+g(d,p) basis set. ^b^CCSD(T) single point on the optimized structure of the B3LYP/6-31+g(d,p) level. ^c^BD(T)/6-31+g(d,p)//B3LYP/6-31+g(d,p) level.

**Table S8.** The calculated zero point and thermodynamic (298.15 K) corrections for all components of the title reaction in the main water-assisted pathways using the B3LYP method.

| Species | ZPE^a^ | E^0a^ | H^0a^ | G^0a^ | S^0b^ |
| --- | --- | --- | --- | --- | --- |
| Thiophene | 0.0666 | 0.0707 | 0.0717 | 0.0394 | 67.924 |
| HO_2_ | 0.0141 | 0.0170 | 0.0179 | -0.0081 | 54.704 |
| H_2_O | 0.0213 | 0.0241 | 0.0251 | 0.0030 | 46.477 |
| 1WHA | 0.0394 | 0.0450 | 0.0459 | 0.0109 | 73.692 |
| 1WHB | 0.0379 | 0.0444 | 0.0454 | 0.0072 | 80.2820 |
| 1WHC | 0.0372 | 0.0424 | 0.0433 | 0.0086 | 73.1670 |
| 1WTA | 0.0893 | 0.0980 | 0.0989 | 0.0529 | 96.826 |
| 1WTB | 0.0892 | 0.0980 | 0.0989 | 0.0524 | 97.790 |
| 1WTC | 0.0895 | 0.0979 | 0.0988 | 0.0539 | 94.509 |
| 1WTD | 0.0893 | 0.0978 | 0.0988 | 0.0531 | 96.247 |
| CR-TS1-1WHA | 0.1074 | 0.1187 | 0.1197 | 0.0655 | 113.911 |
| CR-TS1-1WTB | 0.1060 | 0.1182 | 0.1191 | 0.0629 | 118.324 |
| CR-TS2-1WTA | 0.1060 | 0.1182 | 0.1191 | 0.0632 | 117.692 |
| CR-TS3-1WHA | 0.1072 | 0.1187 | 0.1196 | 0.0650 | 114.905 |
| CR-TS4-1WHA | 0.1073 | 0.1188 | 0.1197 | 0.0637 | 117.952 |
| CR-TS4-1WTB | 0.1067 | 0.1184 | 0.1193 | 0.0651 | 114.128 |
| TS1-1WHA | 0.1073 | 0.1172 | 0.1181 | 0.0702 | 100.924 |
| TS1-1WTB | 0.1065 | 0.1169 | 0.1178 | 0.0685 | 103.895 |
| TS2-1WHA | 0.1072 | 0.1072 | 0.1181 | 0.0701 | 101.033 |
| TS2-1WTA | 0.1065 | 0.1169 | 0.1178 | 0.0689 | 103.100 |
| TS3-1WTA | 0.1051 | 0.1156 | 0.1166 | 0.0677 | 102.759 |
| TS3-1WHA | 0.1047 | 0.1155 | 0.1164 | 0.0662 | 105.822 |
| TS4-1WHA | 0.1025 | 0.1128 | 0.1137 | 0.0652 | 102.119 |
| TS4-1WTB | 0.1030 | 0.1133 | 0.1142 | 0.0658 | 101.957 |
| TS5-1WHA | 0.1054 | 0.1159 | 0.1169 | 0.0675 | 104.031 |
| TS5-1WTB | 0.1057 | 0.1161 | 0.1170 | 0.0680 | 103.051 |
| TS8a-1WHA | 0.1056 | 0.1056 | 0.1170 | 0.0675 | 104.133 |
| TS8a-1WTA | 0.1058 | 0.1162 | 0.1171 | 0.0679 | 103.568 |
| IN8a-1WHA | 0.1088 | 0.1187 | 0.1197 | 0.0725 | 99.276 |
| IN8a-1WTA | 0.1072 | 0.1182 | 0.1191 | 0.0678 | 108.022 |
| CP-TS1-1WAH1 | 0.1081 | 0.1185 | 0.1194 | 0.0706 | 102.844 |
| CP-TS1-1WTB1 | 0.1074 | 0.1182 | 0.1191 | 0.0694 | 104.572 |
| CP-TS1-1WAH2 | 0.1080 | 0.1184 | 0.1193 | 0.1193 | 102.866 |
| CP-TS1-1WTB2 | 0.1070 | 0.1180 | 0.1190 | 0.0681 | 107.194 |
| CP-TS4-1WHA | 0.1081 | 0.1185 | 0.1194 | 0.0706 | 102.680 |
| CP-TS4-1WTB | 0.1072 | 0.1181 | 0.1190 | 0.0689 | 105.596 |
| CP-TS2-1WHA | 0.1082 | 0.1186 | 0.1195 | 0.0708 | 102.565 |
| CP-TS2-1WTA | 0.1075 | 0.1182 | 0.1192 | 0.0694 | 104.703 |
| CP-TS3-1WTA | 0.1051 | 0.1161 | 0.1171 | 0.0662 | 107.108 |
| CP-TS3-1WHA | 0.1060 | 0.1167 | 0.1176 | 0.0676 | 105.279 |
| CP-TS5-1WHA | 0.1074 | 0.1177 | 0.1186 | 0.0697 | 102.986 |
| CP-TS5-1WTB | 0.1064 | 0.1171 | 0.1180 | 0.0678 | 105.764 |
| CP-TS8a-1WHA | 0.1053 | 0.1172 | 0.1182 | 0.0638 | 114.333 |
| CP-TS8a-1WTA | 0.1049 | 0.1172 | 0.1182 | 0.0636 | 114.882 |
| OH | 0.0084 | 0.0108 | 0.0117 | -0.0085 | 42.599 |
| P1 | 0.0832 | 0.0905 | 0.0915 | 0.0507 | 85.791 |
| P2 | 0.0837 | 0.0908 | 0.0917 | 0.0515 | 84.731 |
| C_4_H_4_SO | 0.0695 | 0.0748 | 0.0758 | 0.0405 | 74.168 |
| SC_4_H_4_O | 0.0691 | 0.0754 | 0.0764 | 0.0378 | 81.231 |
| SCHOCHC_2_H_2_ | 0.0710 | 0.0757 | 0.0767 | 0.0426 | 71.646 |

^a^ in Hatree and ^b^in cal mol^-1^.

**Table S9.** The relative energies corrected by the zero energy and thermodynamic (298.15 K) parameters for all components of the title reaction in the other water-assisted pathways computed by the B3LYP method (unit of all numbers is kcal mol^-1^).

| Species | ΔE + ZPE | ΔE^0^ | ΔH^0^ | ΔG^0^ | TΔS^0^ |
| --- | --- | --- | --- | --- | --- |
| R | 0.00 | 0.00 | 0.00 | 0.00 | 0.00 |
| CR-TS1-1WHB | -3.75 | -2.01 | -3.20 | 9.42 | -12.62 |
| CR-TS1-1WTA | -10.81 | -9.79 | -10.98 | 5.36 | -16.34 |
| CR-TS1-1WTA2 | -5.43 | -3.45 | -4.63 | 8.39 | -13.02 |
| CR-TS2-1WHB | -7.94 | -6.75 | -7.94 | 8.45 | -16.39 |
| CR-TS2-1WHC | -5.94 | -4.42 | -5.60 | 9.56 | -15.16 |
| CR-TS2-1WTA | -6.29 | -4.78 | -5.97 | 9.36 | -15.33 |
| CR-TS2-1WTD | -3.82 | -1.74 | -2.92 | 8.13 | -11.06 |
| CR-TS3-1WHA | -10.36 | -9.31 | -10.49 | 5.67 | -16.16 |
| CR-TS3-1WHB | -7.25 | -6.11 | -7.29 | 9.51 | -16.80 |
| CR-TS3-1WTA | -6.29 | -4.78 | -5.96 | 9.32 | -15.28 |
| CR-TS3-1WTB | -4.26 | -2.22 | -3.40 | 9.41 | -12.81 |
| CR-TS4-1WHA | -10.16 | -9.08 | -10.26 | 4.99 | -15.25 |
| CR-TS4-1WTA | 8.69 | 10.04 | 8.85 | 25.21 | -16.35 |
| CR-TS4-1WTB | -7.94 | -6.75 | -7.94 | 8.45 | -16.39 |
| IN-TS8a-1WHA | -8.08 | -7.95 | -9.14 | 11.68 | -20.82 |
| IN-TS8a-1WHB | -8.08 | -7.95 | -9.14 | 11.68 | -20.82 |
| IN-TS8a-1WHC | -3.96 | -3.23 | -4.41 | 13.63 | -18.04 |
| IN-TS8a-1WHC2 | -4.80 | -4.21 | -5.39 | 13.84 | -19.23 |
| IN-TS8a-1WTA2a | -4.71 | -3.92 | -5.11 | 13.11 | -18.21 |
| IN-TS8a-1WTA2b | -3.58 | -2.40 | -3.59 | 13.30 | -16.88 |
| IN-TS8a-1WTB2 | -3.35 | -2.13 | -3.32 | 13.46 | -16.78 |
| TS1-1WHB | 12.52 | 13.06 | 11.87 | 30.27 | -18.39 |
| TS1-1WTA | 12.07 | 12.39 | 11.21 | 31.17 | -19.96 |
| TS1-1WTA2 | 14.90 | 16.09 | 14.90 | 31.31 | -16.40 |
| TS2-1WHB | 7.30 | 7.70 | 6.52 | 26.00 | -19.48 |
| TS2-1WHC | 8.60 | 9.27 | 8.09 | 25.88 | -17.80 |
| TS2-1WTD | 11.33 | 12.40 | 11.21 | 27.55 | -16.34 |
| TS3-1WHA | 28.18 | 28.81 | 27.63 | 46.50 | -18.87 |
| TS3-1WHB | 27.05 | 27.82 | 26.64 | 45.55 | -18.91 |
| TS3-1WTB | 31.29 | 32.75 | 31.56 | 47.60 | -16.04 |
| TS4-1WHA | 51.42 | 51.76 | 50.58 | 70.55 | -19.97 |
| TS4-1WTA | 55.11 | 56.22 | 55.03 | 72.04 | -17.01 |
| TS5-1WHA | 17.64 | 17.96 | 16.77 | 36.72 | -19.94 |
| TS5-1WHB | 15.58 | 16.10 | 14.91 | 34.26 | -19.35 |
| TS5-1WTA | 19.37 | 20.78 | 19.60 | 35.54 | -15.94 |
| TS5-1WTB | 14.85 | 15.17 | 13.98 | 33.66 | -19.68 |
| TS5-1WTB2 | 15.27 | 15.63 | 14.44 | 34.14 | -19.70 |
| TS8a-1WHA | 6.56 | 7.02 | 5.83 | 25.20 | -19.37 |
| TS8a-1WHB | 5.73 | 6.38 | 5.19 | 24.13 | -18.94 |
| TS8a-1WHC | 5.43 | 5.95 | 4.77 | 23.56 | -18.79 |
| TS8a-1WHC2 | 4.48 | 4.88 | 3.70 | 23.28 | -19.58 |
| TS8a-1WTA2a | 3.84 | 4.24 | 3.05 | 22.59 | -19.54 |
| TS8a-1WTA2b | 7.70 | 9.04 | 7.85 | 24.40 | -16.54 |
| TS8a-1WTB | 8.23 | 9.60 | 8.42 | 24.80 | -16.38 |
| CP-TS1-1WHA2 | 5.21 | 5.58 | 4.39 | 24.25 | -19.85 |
| CP-TS1-WHB | 7.75 | 8.56 | 7.38 | 25.44 | -18.06 |
| CP-TS1-WHB2 | 6.71 | 7.36 | 6.17 | 25.11 | -18.93 |
| CP-TS1-1WTA2a | 7.52 | 8.21 | 7.02 | 25.96 | -18.94 |
| CP-TS1-1WTA2b | 9.21 | 10.53 | 9.34 | 25.91 | -16.56 |
| CP-TS1-1WTB2a | 7.53 | 8.13 | 6.94 | 26.18 | -19.24 |
| CP-TS1-1WTB2b | 7.73 | 8.51 | 7.32 | 25.81 | -18.48 |
| CP-TS2-1WHB | -2.17 | -1.49 | -2.68 | 15.95 | -18.63 |
| CP-TS2-1WHC | -2.34 | -1.69 | -2.87 | 15.38 | -18.25 |
| CP-TS2-1WTD | -0.59 | 0.40 | -0.79 | 17.07 | -17.86 |
| CP-TS3-1WHA | 4.23 | 4.82 | 3.63 | 22.67 | -19.03 |
| CP-TS3-1WHB | 6.97 | 7.76 | 6.58 | 25.66 | -19.09 |
| CP-TS3-1WTB | 11.78 | 13.86 | 12.67 | 26.63 | -13.96 |
| CP-TS4-1WHA | 5.64 | 6.00 | 4.81 | 24.62 | -19.80 |
| CP-TS4-1WTA | -7.94 | -6.75 | -7.94 | 8.45 | -16.39 |
| CP-TS5-1WHA2 | 17.64 | 17.96 | 16.77 | 36.72 | -19.94 |
| CP-TS5-1WHB | -6.02 | -5.21 | -6.39 | 11.30 | -17.69 |
| CP-TS5-1WTA | -4.66 | -3.24 | -4.43 | 10.75 | -15.18 |
| CP-TS5-1WTB | -8.70 | -8.11 | -9.30 | 9.59 | -18.89 |
| CP-TS5-1WTB2 | 14.85 | 15.17 | 13.98 | 33.66 | -19.68 |
| CP-TS8a-1WHA2 | -18.90 | -17.53 | -18.72 | -2.37 | -16.35 |
| CP-TS8a-1WHB | -15.71 | -14.13 | -15.31 | 1.08 | -16.40 |
| CP-TS8a-1WHC | -15.71 | -14.13 | -15.32 | 1.08 | -16.39 |
| CP-TS8a-1WHC2 | -15.72 | -14.13 | -15.32 | 1.07 | -16.39 |
| CP-TS8a-1WTA2 | -13.02 | -10.40 | -11.59 | 1.21 | -12.80 |
| CP-TS8a-1WTB | -14.96 | -12.62 | -13.80 | -1.36 | -12.44 |

**Table S10** The absolute energies, zero point energies, thermodynamic corrections, and entropies (298.15 K) for all components of the title reaction in the other water-assisted pathways computed by the B3LYP method (unit of E_Absolute_, ZPE, E_Correction_, H_Correction_, and G_Correction_, is Hartree and unit of S is cal mol^-1^).

| Species | E_Absolute_ | ZPE | E_Correction_ | H_Correction_ | G_Correction_ | S |
| --- | --- | --- | --- | --- | --- | --- |
| R | -780.3675 | 0.1020 | 0.1118 | 0.1146 | 0.0343 | 169.105 |
| CR-TS1-1WHB | -780.3772 | 0.1058 | 0.1184 | 0.1193 | 0.0591 | 126.773 |
| CR-TS1-1WTA | -780.3900 | 0.1073 | 0.1187 | 0.1197 | 0.0654 | 114.301 |
| CR-TS1-1WTA2 | -780.3792 | 0.1051 | 0.1180 | 0.1190 | 0.0594 | 125.427 |
| CR-TS2-1WHB | -780.3848 | 0.1067 | 0.1184 | 0.1193 | 0.0651 | 114.144 |
| CR-TS2-1WHC | -780.3809 | 0.1060 | 0.1182 | 0.1191 | 0.0629 | 118.265 |
| CR-TS2-1WTA | -780.3815 | 0.1060 | 0.1182 | 0.1191 | 0.0632 | 117.692 |
| CR-TS2-1WTD | -780.3762 | 0.1047 | 0.1178 | 0.1187 | 0.0560 | 132.024 |
| CR-TS3-1WHA | -780.3892 | 0.1072 | 0.1187 | 0.1196 | 0.0650 | 114.905 |
| CR-TS3-1WHB | -780.3837 | 0.1067 | 0.1183 | 0.1192 | 0.0656 | 112.764 |
| CR-TS3-1WTA | -780.3815 | 0.1060 | 0.1182 | 0.1191 | 0.0631 | 117.846 |
| CR-TS3-1WTB | -780.3771 | 0.1049 | 0.1179 | 0.1189 | 0.0589 | 126.130 |
| CR-TS4-1WHA | -780.3889 | 0.1073 | 0.1188 | 0.1197 | 0.0637 | 117.952 |
| CR-TS4-1WTA | -780.3575 | 0.1059 | 0.1178 | 0.1188 | 0.0645 | 114.266 |
| CR-TS4-1WTB | -780.3848 | 0.1067 | 0.1184 | 0.1193 | 0.0651 | 114.128 |
| IN-TS8a-1WHA | -780.3871 | 0.1088 | 0.1187 | 0.1197 | 0.0725 | 99.279 |
| IN-TS8a-1WHB | -780.3871 | 0.1088 | 0.1187 | 0.1197 | 0.0725 | 99.280 |
| IN-TS8a-1WHC | -780.3791 | 0.1073 | 0.1183 | 0.1192 | 0.0676 | 108.590 |
| IN-TS8a-1WHC2 | -780.3806 | 0.1075 | 0.1183 | 0.1192 | 0.0695 | 104.598 |
| IN-TS8a-1WTA2a | -780.3801 | 0.1072 | 0.1182 | 0.1191 | 0.0678 | 108.022 |
| IN-TS8a-1WTA2b | -780.3776 | 0.1064 | 0.1181 | 0.1190 | 0.0656 | 112.475 |
| IN-TS8a-1WTB2 | -780.3771 | 0.1063 | 0.1181 | 0.1190 | 0.0654 | 112.826 |
| TS1-1WHB | -780.3517 | 0.1062 | 0.1168 | 0.1177 | 0.0667 | 107.408 |
| TS1-1WTA | -780.3529 | 0.1067 | 0.1169 | 0.1179 | 0.0694 | 102.162 |
| TS1-1WTA2 | -780.3465 | 0.1048 | 0.1165 | 0.1174 | 0.0632 | 114.083 |
| TS2-1WHB | -780.3603 | 0.1064 | 0.1169 | 0.1178 | 0.0685 | 103.754 |
| TS2-1WHC | -780.3577 | 0.1060 | 0.1168 | 0.1178 | 0.0658 | 109.410 |
| TS2-1WTD | -780.3523 | 0.1049 | 0.1164 | 0.1173 | 0.0630 | 114.312 |
| TS3-1WHA | -780.3253 | 0.1047 | 0.1155 | 0.1164 | 0.0662 | 105.818 |
| TS3-1WHB | -780.3267 | 0.1044 | 0.1154 | 0.1163 | 0.0661 | 105.678 |
| TS3-1WTB | -780.3185 | 0.1029 | 0.1150 | 0.1160 | 0.0612 | 115.295 |
| TS4-1WHA | -780.2860 | 0.1025 | 0.1128 | 0.1137 | 0.0652 | 102.119 |
| TS4-1WTA | -780.2793 | 0.1017 | 0.1132 | 0.1142 | 0.0609 | 112.065 |
| TS5-1WHA | -780.3431 | 0.1057 | 0.1160 | 0.1170 | 0.0684 | 102.214 |
| TS5-1WHB | -780.3458 | 0.1052 | 0.1158 | 0.1168 | 0.0672 | 104.205 |
| TS5-1WTA | -780.3379 | 0.1034 | 0.1154 | 0.1163 | 0.0614 | 115.652 |
| TS5-1WTB | -780.3475 | 0.1057 | 0.1160 | 0.1170 | 0.0680 | 103.100 |
| TS5-1WTB2 | -780.3468 | 0.1057 | 0.1161 | 0.1170 | 0.0680 | 103.040 |
| TS8a-1WHA | -780.3606 | 0.1056 | 0.1161 | 0.1170 | 0.0675 | 104.133 |
| TS8a-1WHB | -780.3614 | 0.1051 | 0.1159 | 0.1169 | 0.0667 | 105.596 |
| TS8a-1WHC | -780.3623 | 0.1055 | 0.1161 | 0.1170 | 0.0666 | 106.085 |
| TS8a-1WHC2 | -780.3639 | 0.1056 | 0.1160 | 0.1170 | 0.0678 | 103.424 |
| TS8a-1WTA2a | -780.3651 | 0.1058 | 0.1162 | 0.1171 | 0.0679 | 103.568 |
| TS8a-1WTA2b | -780.3568 | 0.1036 | 0.1155 | 0.1165 | 0.0625 | 113.625 |
| TS8a-1WTB | -780.3559 | 0.1035 | 0.1155 | 0.1165 | 0.0622 | 114.176 |
| CP-TS1-1WHA2 | -780.3652 | 0.1081 | 0.1185 | 0.1194 | 0.0707 | 102.515 |
| CP-TS1-WHB | -780.3601 | 0.1070 | 0.1181 | 0.1190 | 0.0674 | 108.545 |
| CP-TS1-WHB2 | -780.3620 | 0.1073 | 0.1181 | 0.1190 | 0.0689 | 105.611 |
| CP-TS1-1WTA2a | -780.3607 | 0.1073 | 0.1181 | 0.1191 | 0.0689 | 105.590 |
| CP-TS1-1WTA2b | -780.3567 | 0.1059 | 0.1178 | 0.1188 | 0.0648 | 113.553 |
| CP-TS1-1WTB2a | -780.3609 | 0.1074 | 0.1182 | 0.1191 | 0.0694 | 104.582 |
| CP-TS1-1WTB2b | -780.3602 | 0.1070 | 0.1180 | 0.1190 | 0.0681 | 107.111 |
| CP-TS2-1WHB | -780.3762 | 0.1073 | 0.1181 | 0.1191 | 0.0684 | 106.636 |
| CP-TS2-1WHC | -780.3766 | 0.1074 | 0.1182 | 0.1192 | 0.0679 | 107.893 |
| CP-TS2-1WTD | -780.3729 | 0.1065 | 0.1179 | 0.1188 | 0.0669 | 109.216 |
| CP-TS3-1WHA | -780.3647 | 0.1060 | 0.1167 | 0.1176 | 0.0676 | 105.269 |
| CP-TS3-1WHB | -780.3600 | 0.1057 | 0.1167 | 0.1177 | 0.0677 | 105.081 |
| CP-TS3-1WTB | -780.3495 | 0.1028 | 0.1159 | 0.1169 | 0.0588 | 122.293 |
| CP-TS4-1WHA | -780.3646 | 0.1081 | 0.1185 | 0.1194 | 0.0706 | 102.680 |
| CP-TS4-1WTA | -780.3848 | 0.1067 | 0.1184 | 0.1193 | 0.0651 | 114.139 |
| CP-TS5-1WHA2 | -780.3431 | 0.1057 | 0.1160 | 0.1170 | 0.0684 | 102.214 |
| CP-TS5-1WHB | -780.3811 | 0.1061 | 0.1171 | 0.1181 | 0.0659 | 109.756 |
| CP-TS5-1WTA | -780.3777 | 0.1048 | 0.1168 | 0.1178 | 0.0616 | 118.207 |
| CP-TS5-1WTB | -780.3857 | 0.1064 | 0.1171 | 0.1180 | 0.0678 | 105.764 |
| CP-TS5-1WTB2 | -780.3475 | 0.1057 | 0.1160 | 0.1170 | 0.0680 | 103.101 |
| CP-TS8a-1WHA2 | -780.4008 | 0.1053 | 0.1172 | 0.1182 | 0.0639 | 114.268 |
| CP-TS8a-1WHB | -780.3953 | 0.1049 | 0.1172 | 0.1181 | 0.0639 | 114.110 |
| CP-TS8a-1WHC | -780.3953 | 0.1049 | 0.1172 | 0.1181 | 0.0639 | 114.124 |
| CP-TS8a-1WHC2 | -780.3953 | 0.1049 | 0.1172 | 0.1181 | 0.0639 | 114.141 |
| CP-TS8a-1WTA2 | -780.3888 | 0.1026 | 0.1165 | 0.1175 | 0.0575 | 126.186 |
| CP-TS8a-1WTB | -780.3924 | 0.1032 | 0.1167 | 0.1176 | 0.0571 | 127.381 |

**Table S11.** Imaginary Frequency of obtained transition states in the naked and water assisted reactions.

| Saddle point | Imaginary frequency |
| --- | --- |
| ST1 | -552 |
| TS1-1WHA | -504 |
| TS1-1WTA | -459 |
| TS1-1WTA2 | -508 |
| TS1-1WTB | -497 |
| TS2 | -502 |
| TS2-1WHA | -502 |
| TS2-1WHB | -448 |
| TS2-1WHC | -450 |
| TS2-1WTA | -440 |
| TS2-1WTD | -513 |
| TS3 | -804 |
| TS3-1WHA | -798 |
| TS3-1WTA | -750 |
| TS3-1WHB | -781 |
| TS3-1WTB | -707 |
| TS4 | -1246 |
| TS4-1WHA | -1549 |
| TS4-1WTA | -1323 |
| TS4-1WTB | -1375 |
| TS5 | -541 |
| TS5-1WHA | -543 |
| TS5-1WHB | -472 |
| TS5-1WTA | -520 |
| TS5-1WTB | -449 |
| TS5-1WTB2 | -433 |
| TS8a | -516 |
| TS8a-1WHA | -487 |
| TS8a-1WTA | -454 |
| TS8a-1WTA2 | -497 |
| TS8a-1WHB | -449 |
| TS8a-1WHC | -470 |
| TS8a-1WHC2 | -450 |
| TS8a-1WTB | -509 |

**Table S12.** The calculated rate constants (in L mol^−1^s^−1^) for naked pathways of the C_4_H_4_S +HO_2_ reaction computed at the BD(T)/6-31+g(d,p) level.

| T[K] | k_P1_ | k_P1′_ | k_P2_ | k_P3_ | k_P4_ | k_P5_ |
| --- | --- | --- | --- | --- | --- | --- |
| 300 | 2.01E-05 | 1.64E-33 | 1.77E-02 | 4.20E-18 | 2.23E-03 | 7.60E-07 |
| 400 | 1.80E-02 | 7.28E-24 | 2.96E+00 | 7.14E-12 | 6.10E-01 | 1.27E-03 |
| 500 | 1.26E+00 | 7.66E-18 | 7.52E+01 | 5.00E-08 | 2.01E+01 | 1.44E-01 |
| 600 | 2.40E+01 | 9.59E-14 | 7.26E+02 | 2.11E-05 | 2.31E+02 | 4.05E+00 |
| 700 | 2.12E+02 | 9.05E-11 | 3.96E+03 | 1.74E-03 | 1.45E+03 | 4.90E+01 |
| 800 | 1.16E+03 | 1.66E-08 | 1.50E+04 | 5.09E-02 | 6.16E+03 | 3.40E+02 |
| 900 | 4.52E+03 | 1.01E-06 | 4.40E+04 | 7.40E-01 | 2.00E+04 | 1.60E+03 |
| 1000 | 1.39E+04 | 2.82E-05 | 1.08E+05 | 6.54E+00 | 5.33E+04 | 5.69E+03 |
| 1100 | 3.60E+04 | 4.44E-04 | 2.32E+05 | 4.01E+01 | 1.22E+05 | 1.64E+04 |
| 1200 | 8.12E+04 | 4.54E-03 | 4.48E+05 | 1.87E+02 | 2.49E+05 | 4.04E+04 |
| 1300 | 1.65E+05 | 3.32E-02 | 7.99E+05 | 7.00E+02 | 4.64E+05 | 8.80E+04 |
| 1400 | 3.08E+05 | 1.86E-01 | 1.33E+06 | 2.21E+03 | 8.02E+05 | 1.74E+05 |
| 1500 | 5.38E+05 | 8.43E-01 | 2.11E+06 | 6.08E+03 | 1.31E+06 | 3.17E+05 |
| 1600 | 8.86E+05 | 3.21E+00 | 3.19E+06 | 1.49E+04 | 2.02E+06 | 5.42E+05 |
| 1700 | 1.39E+06 | 1.06E+01 | 4.65E+06 | 3.34E+04 | 3.01E+06 | 8.78E+05 |
| 1800 | 2.10E+06 | 3.08E+01 | 6.57E+06 | 6.90E+04 | 4.33E+06 | 1.36E+06 |
| 1900 | 3.06E+06 | 8.10E+01 | 9.02E+06 | 1.33E+05 | 6.04E+06 | 2.03E+06 |
| 2000 | 4.34E+06 | 1.95E+02 | 1.21E+07 | 2.43E+05 | 8.21E+06 | 2.93E+06 |
| 2100 | 5.98E+06 | 4.36E+02 | 1.59E+07 | 4.22E+05 | 1.09E+07 | 4.11E+06 |
| 2200 | 8.07E+06 | 9.10E+02 | 2.05E+07 | 7.00E+05 | 1.42E+07 | 5.63E+06 |
| 2300 | 1.07E+07 | 1.80E+03 | 2.60E+07 | 1.12E+06 | 1.82E+07 | 7.54E+06 |
| 2400 | 1.38E+07 | 3.37E+03 | 3.25E+07 | 1.73E+06 | 2.30E+07 | 9.90E+06 |
| 2500 | 1.77E+07 | 6.04E+03 | 4.02E+07 | 2.60E+06 | 2.86E+07 | 1.28E+07 |
| 2600 | 2.23E+07 | 1.04E+04 | 4.90E+07 | 3.79E+06 | 3.52E+07 | 1.63E+07 |
| 2700 | 2.77E+07 | 1.73E+04 | 5.92E+07 | 5.41E+06 | 4.28E+07 | 2.04E+07 |
| 2800 | 3.41E+07 | 2.78E+04 | 7.09E+07 | 7.56E+06 | 5.15E+07 | 2.52E+07 |
| 2900 | 4.15E+07 | 4.35E+04 | 8.41E+07 | 1.04E+07 | 6.13E+07 | 3.09E+07 |
| 3000 | 4.99E+07 | 6.62E+04 | 9.90E+07 | 1.39E+07 | 7.25E+07 | 3.74E+07 |

**Table S13.** The calculated rate constants (in L mol^−1^s^−1^) for water-assisted pathways of the C_4_H_4_S + HO_2_ reaction calculated at the BD(T)/6-31+g(d,p) level.

| T[K] | k_P1-1WHA_ | k_P1-1WTB_ | k_P1′-1WHA_ | k_P1′-1WTB_ | k_P2-1WHA_ | k_P2-1WTA_ | k_P3-1WHA_ | k_P3-1WTA_ | k_P4-1WHA_ | k_P4-1WTA_ | k_P5-1WHA_ | k_P5-1WTB_ |
| --- | --- | --- | --- | --- | --- | --- | --- | --- | --- | --- | --- | --- |
| 300 | 7.20E-07 | 2.03E-03 | 4.32E-34 | 8.47E-30 | 1.48E-03 | 2.58E-01 | 2.34E-19 | 4.50E-15 | 7.45E-05 | 5.92E-02 | 1.22E-09 | 1.50E-04 |
| 400 | 7.93E-04 | 2.00E-01 | 3.93E-25 | 8.77E-22 | 2.45E-01 | 5.15E+00 | 5.97E-13 | 2.22E-10 | 2.89E-02 | 1.37E+00 | 5.07E-06 | 1.93E-02 |
| 500 | 6.26E-02 | 3.66E+00 | 4.36E-19 | 1.22E-16 | 6.22E+00 | 3.58E+01 | 5.37E-09 | 1.77E-07 | 1.12E+00 | 1.03E+01 | 1.03E-03 | 4.37E-01 |
| 600 | 1.29E+00 | 2.81E+01 | 6.42E-15 | 4.06E-13 | 5.99E+01 | 1.44E+02 | 2.68E-06 | 1.74E-05 | 1.41E+01 | 4.38E+01 | 4.47E-02 | 4.04E+00 |
| 700 | 1.20E+01 | 1.30E+02 | 7.05E-12 | 1.49E-10 | 3.26E+02 | 4.18E+02 | 2.50E-04 | 4.99E-04 | 9.29E+01 | 1.31E+02 | 7.63E-01 | 2.18E+01 |
| 800 | 6.82E+01 | 4.33E+02 | 1.47E-09 | 1.36E-08 | 1.23E+03 | 9.83E+02 | 8.05E-03 | 6.60E-03 | 4.23E+02 | 3.18E+02 | 7.00E+00 | 8.26E+01 |
| 900 | 2.75E+02 | 1.15E+03 | 9.97E-08 | 4.78E-07 | 3.62E+03 | 2.00E+03 | 1.26E-01 | 5.16E-02 | 1.46E+03 | 6.65E+02 | 4.08E+01 | 2.43E+02 |
| 1000 | 8.67E+02 | 2.61E+03 | 3.05E-06 | 8.62E-06 | 8.88E+03 | 3.64E+03 | 1.18E+00 | 2.77E-01 | 4.09E+03 | 1.24E+03 | 1.71E+02 | 5.97E+02 |
| 1100 | 2.28E+03 | 5.24E+03 | 5.20E-05 | 9.51E-05 | 1.90E+04 | 6.11E+03 | 7.61E+00 | 1.13E+00 | 9.68E+03 | 2.14E+03 | 5.63E+02 | 1.28E+03 |
| 1200 | 5.24E+03 | 9.58E+03 | 5.70E-04 | 7.23E-04 | 3.68E+04 | 9.64E+03 | 3.69E+01 | 3.74E+00 | 2.02E+04 | 3.44E+03 | 1.54E+03 | 2.47E+03 |
| 1300 | 1.08E+04 | 1.63E+04 | 4.42E-03 | 4.12E-03 | 6.55E+04 | 1.45E+04 | 1.43E+02 | 1.05E+01 | 3.83E+04 | 5.24E+03 | 3.65E+03 | 4.40E+03 |
| 1400 | 2.04E+04 | 2.61E+04 | 2.61E-02 | 1.86E-02 | 1.09E+05 | 2.08E+04 | 4.66E+02 | 2.59E+01 | 6.70E+04 | 7.64E+03 | 7.73E+03 | 7.32E+03 |
| 1500 | 3.60E+04 | 3.98E+04 | 1.24E-01 | 7.02E-02 | 1.73E+05 | 2.90E+04 | 1.32E+03 | 5.74E+01 | 1.10E+05 | 1.08E+04 | 1.50E+04 | 1.16E+04 |
| 1600 | 5.98E+04 | 5.84E+04 | 4.93E-01 | 2.27E-01 | 2.62E+05 | 3.92E+04 | 3.30E+03 | 1.17E+02 | 1.72E+05 | 1.47E+04 | 2.69E+04 | 1.74E+04 |
| 1700 | 9.48E+04 | 8.28E+04 | 1.68E+00 | 6.48E-01 | 3.81E+05 | 5.17E+04 | 7.54E+03 | 2.21E+02 | 2.58E+05 | 1.95E+04 | 4.54E+04 | 2.54E+04 |
| 1800 | 1.44E+05 | 1.14E+05 | 5.08E+00 | 1.66E+00 | 5.38E+05 | 6.68E+04 | 1.59E+04 | 3.94E+02 | 3.72E+05 | 2.54E+04 | 7.29E+04 | 3.57E+04 |
| 1900 | 2.11E+05 | 1.53E+05 | 1.38E+01 | 3.91E+00 | 7.39E+05 | 8.47E+04 | 3.11E+04 | 6.67E+02 | 5.21E+05 | 3.25E+04 | 1.12E+05 | 4.90E+04 |
| 2000 | 3.01E+05 | 2.02E+05 | 3.41E+01 | 8.51E+00 | 9.90E+05 | 1.06E+05 | 5.75E+04 | 1.08E+03 | 7.11E+05 | 4.08E+04 | 1.66E+05 | 6.55E+04 |
| 2100 | 4.17E+05 | 2.60E+05 | 7.81E+01 | 1.73E+01 | 1.30E+06 | 1.30E+05 | 1.01E+05 | 1.68E+03 | 9.48E+05 | 5.05E+04 | 2.38E+05 | 8.59E+04 |
| 2200 | 5.65E+05 | 3.30E+05 | 1.67E+02 | 3.33E+01 | 1.68E+06 | 1.59E+05 | 1.70E+05 | 2.53E+03 | 1.24E+06 | 6.17E+04 | 3.32E+05 | 1.11E+05 |
| 2300 | 7.50E+05 | 4.13E+05 | 3.37E+02 | 6.09E+01 | 2.13E+06 | 1.91E+05 | 2.75E+05 | 3.70E+03 | 1.59E+06 | 7.45E+04 | 4.52E+05 | 1.40E+05 |
| 2400 | 9.78E+05 | 5.09E+05 | 6.44E+02 | 1.07E+02 | 2.66E+06 | 2.27E+05 | 4.29E+05 | 5.28E+03 | 2.00E+06 | 8.90E+04 | 6.02E+05 | 1.75E+05 |
| 2500 | 1.25E+06 | 6.21E+05 | 1.18E+03 | 1.79E+02 | 3.29E+06 | 2.69E+05 | 6.49E+05 | 7.35E+03 | 2.50E+06 | 1.05E+05 | 7.86E+05 | 2.15E+05 |
| 2600 | 1.59E+06 | 7.49E+05 | 2.06E+03 | 2.91E+02 | 4.02E+06 | 3.14E+05 | 9.57E+05 | 1.00E+04 | 3.07E+06 | 1.24E+05 | 1.01E+06 | 2.61E+05 |
| 2700 | 1.98E+06 | 8.95E+05 | 3.48E+03 | 4.58E+02 | 4.85E+06 | 3.66E+05 | 1.38E+06 | 1.34E+04 | 3.72E+06 | 1.44E+05 | 1.27E+06 | 3.15E+05 |
| 2800 | 2.44E+06 | 1.06E+06 | 5.69E+03 | 7.01E+02 | 5.81E+06 | 4.22E+05 | 1.94E+06 | 1.76E+04 | 4.48E+06 | 1.67E+05 | 1.59E+06 | 3.75E+05 |
| 2900 | 2.98E+06 | 1.25E+06 | 9.02E+03 | 1.05E+03 | 6.89E+06 | 4.84E+05 | 2.67E+06 | 2.29E+04 | 5.33E+06 | 1.92E+05 | 1.95E+06 | 4.43E+05 |
| 3000 | 3.59E+06 | 1.45E+06 | 1.39E+04 | 1.52E+03 | 8.11E+06 | 5.53E+05 | 3.62E+06 | 2.92E+04 | 6.29E+06 | 2.19E+05 | 2.37E+06 | 5.19E+05 |

**Table S14:** The reactivity parameter values by means of the condensed Fukui function for the stationary points of the P1 and P5 formation path started from the 1WHA and thiophene species.

| **Atom** | **Thio+1WHA** | | | **CR-TS1-1WHA** | | | **TS1-1WHA** | | | **CP-TS1-1WAH1** | | |
| --- | --- | --- | --- | --- | --- | --- | --- | --- | --- | --- | --- | --- |
|  | $f_{X}^{+}$ | $f_{X}^{-}$ | $f_{X}^{0}$ | $f_{X}^{+}$ | $f_{X}^{-}$ | $f_{X}^{0}$ | $f_{X}^{+}$ | $f_{X}^{-}$ | $f_{X}^{0}$ | $f_{X}^{+}$ | $f_{X}^{-}$ | $f_{X}^{0}$ |
| C1 | -0.177 | -0.109 | -0.143 | -0.079 | -0.004 | -0.041 | -0.129 | -0.148 | -0.139 | -0.186 | -0.199 | -0.193 |
| C2 | -0.108 | -0.073 | -0.090 | -0.050 | -0.022 | -0.036 | -0.051 | -0.043 | -0.047 | -0.034 | -0.049 | -0.042 |
| C3 | -0.108 | -0.073 | -0.091 | -0.069 | -0.029 | -0.049 | -0.056 | -0.049 | -0.053 | -0.076 | -0.067 | -0.071 |
| C4 | -0.177 | -0.110 | -0.143 | -0.108 | -0.045 | -0.076 | -0.051 | -0.067 | -0.059 | -0.040 | -0.076 | -0.058 |
| S5 | -0.185 | -0.267 | -0.226 | -0.105 | -0.060 | -0.083 | -0.249 | -0.178 | -0.214 | -0.306 | -0.192 | -0.249 |
| H6 | -0.066 | -0.134 | -0.100 | -0.023 | -0.011 | -0.017 | -0.050 | -0.059 | -0.055 | -0.065 | -0.076 | -0.070 |
| H7 | -0.056 | -0.049 | -0.053 | -0.029 | -0.014 | -0.021 | -0.042 | -0.044 | -0.043 | -0.053 | -0.056 | -0.055 |
| H8 | -0.056 | -0.050 | -0.053 | -0.037 | -0.023 | -0.030 | -0.039 | -0.038 | -0.039 | -0.047 | -0.047 | -0.047 |
| H9 | -0.066 | -0.135 | -0.101 | -0.044 | -0.028 | -0.036 | -0.039 | -0.046 | -0.043 | -0.039 | -0.052 | -0.045 |
| O10 | -0.387 | -0.285 | -0.336 | -0.188 | -0.239 | -0.214 | -0.103 | -0.089 | -0.096 | -0.048 | -0.050 | -0.049 |
| H11 | -0.128 | -0.132 | -0.130 | -0.038 | -0.066 | -0.052 | -0.036 | -0.038 | -0.037 | -0.024 | -0.027 | -0.026 |
| O12 | -0.486 | -0.583 | -0.535 | -0.188 | -0.418 | -0.303 | -0.113 | -0.138 | -0.126 | -0.053 | -0.049 | -0.051 |
| O13 | -0.653 | -0.342 | -0.497 | -0.016 | -0.007 | -0.012 | -0.016 | -0.020 | -0.018 | -0.011 | -0.018 | -0.014 |
| H14 | -0.173 | -0.329 | -0.251 | -0.021 | -0.026 | -0.024 | -0.023 | -0.027 | -0.025 | -0.018 | -0.026 | -0.022 |
| H15 | -0.173 | -0.329 | -0.251 | -0.005 | -0.008 | -0.007 | -0.002 | -0.015 | -0.008 | 0.000 | -0.017 | -0.009 |
| **Atom** | **CP-TS1-1WAH2** | | | **P1+H_2_O** | | | **TS5-1WHA** | | | **CP-TS5-1WHA** | | |
|  | $f_{X}^{+}$ | $f_{X}^{-}$ | $f_{X}^{0}$ | $f_{X}^{+}$ | $f_{X}^{-}$ | $f_{X}^{0}$ | $f_{X}^{+}$ | $f_{X}^{-}$ | $f_{X}^{0}$ | $f_{X}^{+}$ | $f_{X}^{-}$ | $f_{X}^{0}$ |
| C1 | -0.183 | -0.203 | -0.193 | -0.183 | -0.203 | -0.193 | -0.106 | -0.157 | -0.132 | -0.038 | -0.024 | -0.031 |
| C2 | -0.034 | -0.048 | -0.041 | -0.036 | -0.049 | -0.042 | -0.033 | -0.029 | -0.031 | -0.032 | -0.019 | -0.025 |
| C3 | -0.073 | -0.058 | -0.065 | -0.074 | -0.059 | -0.067 | -0.054 | -0.041 | -0.048 | -0.151 | -0.031 | -0.091 |
| C4 | -0.039 | -0.064 | -0.052 | -0.041 | -0.065 | -0.053 | -0.042 | -0.045 | -0.044 | -0.090 | -0.025 | -0.058 |
| S5 | -0.310 | -0.206 | -0.258 | -0.313 | -0.209 | -0.261 | -0.259 | -0.198 | -0.229 | -0.271 | -0.086 | -0.178 |
| H6 | -0.066 | -0.081 | -0.074 | -0.066 | -0.082 | -0.074 | -0.047 | -0.062 | -0.054 | -0.041 | -0.027 | -0.034 |
| H7 | -0.056 | -0.057 | -0.057 | -0.057 | -0.059 | -0.058 | -0.047 | -0.040 | -0.044 | -0.040 | -0.025 | -0.032 |
| H8 | -0.045 | -0.042 | -0.043 | -0.046 | -0.043 | -0.044 | -0.036 | -0.033 | -0.035 | -0.052 | -0.024 | -0.038 |
| H9 | -0.039 | -0.048 | -0.043 | -0.039 | -0.049 | -0.044 | -0.036 | -0.038 | -0.037 | -0.041 | -0.023 | -0.032 |
| O10 | -0.042 | -0.050 | -0.046 | -0.047 | -0.062 | -0.054 | -0.086 | -0.032 | -0.059 | -0.068 | -0.020 | -0.044 |
| O11 | -0.064 | -0.074 | -0.069 | -0.065 | -0.079 | -0.072 | -0.169 | -0.221 | -0.195 | -0.112 | -0.515 | -0.313 |
| H12 | -0.023 | -0.029 | -0.026 | -0.032 | -0.042 | -0.037 | -0.050 | -0.069 | -0.060 | -0.023 | -0.128 | -0.075 |
| O13 | -0.016 | -0.017 | -0.017 | -0.653 | -0.342 | -0.497 | -0.021 | -0.018 | -0.020 | -0.021 | -0.010 | -0.016 |
| H14 | 0.003 | -0.007 | -0.002 | -0.173 | -0.329 | -0.251 | 0.004 | 0.001 | 0.002 | -0.001 | -0.013 | -0.007 |
| H15 | -0.013 | -0.017 | -0.015 | -0.173 | -0.329 | -0.251 | -0.016 | -0.017 | -0.017 | -0.020 | -0.030 | -0.025 |
| **Atom** | **P5+H_2_O** | | |  |  |  |  |  |  |  |  |  |
|  | $f_{X}^{+}$ | $f_{X}^{-}$ | $f_{X}^{0}$ |  |  |  |  |  |  |  |  |  |
| C1 | -0.039 | -0.067 | -0.053 |  |  |  |  |  |  |  |  |  |
| C2 | -0.039 | -0.071 | -0.055 |  |  |  |  |  |  |  |  |  |
| C3 | -0.162 | -0.112 | -0.137 |  |  |  |  |  |  |  |  |  |
| C4 | -0.090 | -0.117 | -0.104 |  |  |  |  |  |  |  |  |  |
| S5 | -0.383 | -0.238 | -0.311 |  |  |  |  |  |  |  |  |  |
| H6 | -0.044 | -0.117 | -0.080 |  |  |  |  |  |  |  |  |  |
| H7 | -0.045 | -0.063 | -0.054 |  |  |  |  |  |  |  |  |  |
| H8 | -0.064 | -0.064 | -0.064 |  |  |  |  |  |  |  |  |  |
| H9 | -0.049 | -0.077 | -0.063 |  |  |  |  |  |  |  |  |  |
| O10 | -0.085 | -0.074 | -0.079 |  |  |  |  |  |  |  |  |  |
| O11 | -0.801 | -0.757 | -0.779 |  |  |  |  |  |  |  |  |  |
| H12 | -0.199 | -0.243 | -0.221 |  |  |  |  |  |  |  |  |  |
| O13 | -0.653 | -0.342 | -0.497 |  |  |  |  |  |  |  |  |  |
| H14 | -0.173 | -0.329 | -0.251 |  |  |  |  |  |  |  |  |  |
| H15 | -0.173 | -0.329 | -0.251 |  |  |  |  |  |  |  |  |  |

**Table S15:** The reactivity parameter values by means of the condensed Fukui function for the stationary points of the P1 and P5 formation path started from the 1WTB and HO_2_ species.

| **Atom** | **HO_2_+1WTB** | | | **CR-TS1-1WTB** | | | **TS1-1WTB** | | | **CP-TS1-1WTB1** | | |
| --- | --- | --- | --- | --- | --- | --- | --- | --- | --- | --- | --- | --- |
|  | $f_{X}^{+}$ | $f_{X}^{-}$ | $f_{X}^{0}$ | $f_{X}^{+}$ | $f_{X}^{-}$ | $f_{X}^{0}$ | $f_{X}^{+}$ | $f_{X}^{-}$ | $f_{X}^{0}$ | $f_{X}^{+}$ | $f_{X}^{-}$ | $f_{X}^{0}$ |
| C1 | -0.177 | -0.109 | -0.143 | -0.024 | 0.005 | -0.009 | -0.130 | -0.151 | -0.141 | -0.179 | -0.196 | -0.188 |
| C2 | -0.108 | -0.073 | -0.090 | -0.067 | -0.003 | -0.035 | -0.053 | -0.042 | -0.048 | -0.035 | -0.049 | -0.042 |
| C3 | -0.108 | -0.073 | -0.091 | -0.083 | -0.014 | -0.048 | -0.053 | -0.044 | -0.049 | -0.073 | -0.054 | -0.064 |
| C4 | -0.177 | -0.110 | -0.143 | -0.046 | -0.030 | -0.038 | -0.051 | -0.061 | -0.056 | -0.040 | -0.062 | -0.051 |
| S5 | -0.185 | -0.267 | -0.226 | -0.225 | -0.051 | -0.138 | -0.252 | -0.188 | -0.220 | -0.311 | -0.208 | -0.260 |
| H6 | -0.066 | -0.134 | -0.100 | -0.024 | -0.008 | -0.016 | -0.053 | -0.064 | -0.058 | -0.065 | -0.079 | -0.072 |
| H7 | -0.056 | -0.049 | -0.053 | -0.027 | -0.005 | -0.016 | -0.036 | -0.037 | -0.037 | -0.053 | -0.055 | -0.054 |
| H8 | -0.056 | -0.050 | -0.053 | -0.040 | -0.017 | -0.028 | -0.037 | -0.036 | -0.037 | -0.040 | -0.036 | -0.038 |
| H9 | -0.066 | -0.135 | -0.101 | -0.035 | -0.023 | -0.029 | -0.039 | -0.044 | -0.042 | -0.039 | -0.047 | -0.043 |
| O10 | -0.387 | -0.285 | -0.336 | -0.153 | -0.219 | -0.186 | -0.099 | -0.090 | -0.095 | -0.063 | -0.077 | -0.070 |
| H11 | -0.128 | -0.132 | -0.130 | -0.030 | -0.070 | -0.050 | -0.041 | -0.045 | -0.043 | -0.030 | -0.040 | -0.035 |
| O12 | -0.486 | -0.583 | -0.535 | -0.186 | -0.506 | -0.346 | -0.118 | -0.161 | -0.139 | -0.047 | -0.066 | -0.056 |
| O13 | -0.653 | -0.342 | -0.497 | -0.028 | -0.018 | -0.023 | -0.015 | -0.010 | -0.013 | -0.008 | -0.009 | -0.008 |
| H14 | -0.173 | -0.329 | -0.251 | -0.005 | -0.011 | -0.008 | -0.001 | -0.004 | -0.003 | 0.001 | -0.003 | -0.001 |
| H15 | -0.173 | -0.329 | -0.251 | -0.028 | -0.031 | -0.029 | -0.022 | -0.022 | -0.022 | -0.018 | -0.019 | -0.019 |
| **Atom** | **CP-TS1-1WTB2** | | | **P1+H_2_O** | | | **TS5-1WTB** | | | **CP-TS5-1WTB** | | |
|  | $f_{X}^{+}$ | $f_{X}^{-}$ | $f_{X}^{0}$ | $f_{X}^{+}$ | $f_{X}^{-}$ | $f_{X}^{0}$ | $f_{X}^{+}$ | $f_{X}^{-}$ | $f_{X}^{0}$ | $f_{X}^{+}$ | $f_{X}^{-}$ | $f_{X}^{0}$ |
| C1 | -0.181 | -0.195 | -0.188 | -0.183 | -0.203 | -0.193 | -0.101 | -0.151 | -0.126 | -0.030 | -0.005 | -0.018 |
| C2 | -0.036 | -0.048 | -0.042 | -0.036 | -0.049 | -0.042 | -0.034 | -0.030 | -0.032 | -0.029 | -0.005 | -0.017 |
| C3 | -0.074 | -0.056 | -0.065 | -0.074 | -0.059 | -0.067 | -0.054 | -0.042 | -0.048 | -0.137 | -0.005 | -0.071 |
| C4 | -0.040 | -0.064 | -0.052 | -0.041 | -0.065 | -0.053 | -0.043 | -0.045 | -0.044 | -0.082 | -0.026 | -0.054 |
| S5 | -0.309 | -0.205 | -0.257 | -0.313 | -0.209 | -0.261 | -0.255 | -0.207 | -0.231 | -0.308 | -0.060 | -0.184 |
| H6 | -0.065 | -0.078 | -0.071 | -0.066 | -0.082 | -0.074 | -0.045 | -0.060 | -0.052 | -0.036 | -0.008 | -0.022 |
| H7 | -0.050 | -0.050 | -0.050 | -0.057 | -0.059 | -0.058 | -0.039 | -0.035 | -0.037 | -0.027 | -0.002 | -0.014 |
| H8 | -0.045 | -0.042 | -0.043 | -0.046 | -0.043 | -0.044 | -0.036 | -0.034 | -0.035 | -0.055 | -0.012 | -0.033 |
| H9 | -0.039 | -0.048 | -0.043 | -0.039 | -0.049 | -0.044 | -0.036 | -0.038 | -0.037 | -0.044 | -0.020 | -0.032 |
| O10 | -0.044 | -0.066 | -0.055 | -0.047 | -0.062 | -0.054 | -0.094 | -0.046 | -0.070 | -0.062 | 0.033 | -0.014 |
| O11 | -0.056 | -0.075 | -0.066 | -0.065 | -0.079 | -0.072 | -0.145 | -0.188 | -0.166 | -0.045 | -0.614 | -0.330 |
| H12 | -0.032 | -0.045 | -0.038 | -0.032 | -0.042 | -0.037 | -0.056 | -0.075 | -0.066 | -0.003 | -0.151 | -0.077 |
| O13 | -0.009 | -0.006 | -0.007 | -0.653 | -0.342 | -0.497 | -0.028 | -0.016 | -0.022 | -0.083 | -0.042 | -0.062 |
| H14 | 0.000 | -0.003 | -0.002 | -0.173 | -0.329 | -0.251 | -0.006 | -0.011 | -0.009 | -0.019 | -0.042 | -0.031 |
| H15 | -0.018 | -0.019 | -0.019 | -0.173 | -0.329 | -0.251 | -0.027 | -0.025 | -0.026 | -0.040 | -0.042 | -0.041 |
| **Atom** | **P5+H_2_O** | | |  |  |  |  |  |  |  |  |  |
|  | $f_{X}^{+}$ | $f_{X}^{-}$ | $f_{X}^{0}$ |  |  |  |  |  |  |  |  |  |
| C1 | -0.039 | -0.067 | -0.053 |  |  |  |  |  |  |  |  |  |
| C2 | -0.039 | -0.071 | -0.055 |  |  |  |  |  |  |  |  |  |
| C3 | -0.162 | -0.112 | -0.137 |  |  |  |  |  |  |  |  |  |
| C4 | -0.090 | -0.117 | -0.104 |  |  |  |  |  |  |  |  |  |
| S5 | -0.383 | -0.238 | -0.311 |  |  |  |  |  |  |  |  |  |
| H6 | -0.044 | -0.117 | -0.080 |  |  |  |  |  |  |  |  |  |
| H7 | -0.045 | -0.063 | -0.054 |  |  |  |  |  |  |  |  |  |
| H8 | -0.064 | -0.064 | -0.064 |  |  |  |  |  |  |  |  |  |
| H9 | -0.049 | -0.077 | -0.063 |  |  |  |  |  |  |  |  |  |
| O10 | -0.085 | -0.074 | -0.079 |  |  |  |  |  |  |  |  |  |
| O11 | -0.653 | -0.342 | -0.497 |  |  |  |  |  |  |  |  |  |
| H12 | -0.173 | -0.329 | -0.251 |  |  |  |  |  |  |  |  |  |
| O13 | -0.173 | -0.329 | -0.251 |  |  |  |  |  |  |  |  |  |

**Table S16:** The reactivity parameter values by means of the condensed Fukui function for the stationary points of the P1 formation path proceed by the TS4-1WHA saddle point.

| **Atom** | **Thio+1WHA** | | | | | **CR-TS4-1WHA** | | | **TS4-1WHA** | | | **CP-TS4-1WHA** | | |
| --- | --- | --- | --- | --- | --- | --- | --- | --- | --- | --- | --- | --- | --- | --- |
|  | $f_{X}^{+}$ | | $f_{X}^{-}$ | | $f_{X}^{0}$ | $f_{X}^{+}$ | $f_{X}^{-}$ | $f_{X}^{0}$ | $f_{X}^{+}$ | $f_{X}^{-}$ | $f_{X}^{0}$ | $f_{X}^{+}$ | $f_{X}^{-}$ | $f_{X}^{0}$ |
| C1 | -0.177 | | -0.109 | | -0.143 | -0.088 | -0.030 | -0.059 | -0.040 | -0.061 | -0.051 | -0.040 | -0.062 | -0.051 |
| C2 | -0.108 | | -0.073 | | -0.090 | -0.057 | -0.012 | -0.034 | -0.071 | -0.054 | -0.063 | -0.074 | -0.057 | -0.066 |
| C3 | -0.108 | | -0.073 | | -0.091 | -0.037 | -0.022 | -0.030 | -0.032 | -0.049 | -0.041 | -0.035 | -0.048 | -0.041 |
| C4 | -0.177 | | -0.110 | | -0.143 | -0.088 | -0.039 | -0.063 | -0.172 | -0.192 | -0.182 | -0.178 | -0.198 | -0.188 |
| S5 | -0.185 | | -0.267 | | -0.226 | -0.108 | -0.081 | -0.095 | -0.304 | -0.207 | -0.255 | -0.310 | -0.209 | -0.260 |
| H6 | -0.066 | | -0.134 | | -0.100 | -0.036 | -0.019 | -0.027 | -0.038 | -0.046 | -0.042 | -0.039 | -0.047 | -0.043 |
| H7 | -0.056 | | -0.049 | | -0.053 | -0.027 | -0.011 | -0.019 | -0.043 | -0.040 | -0.041 | -0.044 | -0.041 | -0.043 |
| H8 | -0.056 | | -0.050 | | -0.053 | -0.027 | -0.019 | -0.023 | -0.052 | -0.052 | -0.052 | -0.058 | -0.056 | -0.057 |
| H9 | -0.066 | | -0.135 | | -0.101 | -0.038 | -0.025 | -0.032 | -0.063 | -0.076 | -0.069 | -0.065 | -0.079 | -0.072 |
| O10 | -0.387 | | -0.285 | | -0.336 | -0.194 | -0.221 | -0.208 | -0.039 | -0.047 | -0.043 | -0.044 | -0.050 | -0.047 |
| H11 | -0.128 | | -0.132 | | -0.130 | -0.038 | -0.059 | -0.049 | -0.015 | -0.021 | -0.018 | -0.025 | -0.030 | -0.027 |
| O12 | -0.486 | | -0.583 | | -0.535 | -0.220 | -0.419 | -0.320 | -0.100 | -0.111 | -0.105 | -0.063 | -0.073 | -0.068 |
| O13 | -0.653 | | -0.342 | | -0.497 | -0.019 | -0.012 | -0.016 | -0.004 | -0.006 | -0.005 | -0.010 | -0.014 | -0.012 |
| H14 | -0.173 | | -0.329 | | -0.251 | -0.015 | -0.019 | -0.017 | -0.014 | -0.017 | -0.015 | -0.018 | -0.023 | -0.021 |
| H15 | -0.173 | | -0.329 | | -0.251 | -0.008 | -0.012 | -0.010 | -0.015 | -0.020 | -0.018 | 0.003 | -0.013 | -0.005 |
| **Atom** | **P1+H_2_O** | | | | |  |  |  |  |  |  |  |  |  |
|  | $f_{X}^{+}$ | $f_{X}^{-}$ | | $f_{X}^{0}$ | |  |  |  |  |  |  |  |  |  |
| C1 | -0.183 | -0.203 | | -0.193 | |  |  |  |  |  |  |  |  |  |
| C2 | -0.036 | -0.049 | | -0.042 | |  |  |  |  |  |  |  |  |  |
| C3 | -0.074 | -0.059 | | -0.067 | |  |  |  |  |  |  |  |  |  |
| C4 | -0.041 | -0.065 | | -0.053 | |  |  |  |  |  |  |  |  |  |
| S5 | -0.313 | -0.209 | | -0.261 | |  |  |  |  |  |  |  |  |  |
| H6 | -0.066 | -0.082 | | -0.074 | |  |  |  |  |  |  |  |  |  |
| H7 | -0.057 | -0.059 | | -0.058 | |  |  |  |  |  |  |  |  |  |
| H8 | -0.046 | -0.043 | | -0.044 | |  |  |  |  |  |  |  |  |  |
| H9 | -0.039 | -0.049 | | -0.044 | |  |  |  |  |  |  |  |  |  |
| O10 | -0.047 | -0.062 | | -0.054 | |  |  |  |  |  |  |  |  |  |
| H11 | -0.032 | -0.042 | | -0.037 | |  |  |  |  |  |  |  |  |  |
| O12 | -0.065 | -0.079 | | -0.072 | |  |  |  |  |  |  |  |  |  |
| O13 | -0.653 | -0.342 | | -0.497 | |  |  |  |  |  |  |  |  |  |
| H14 | -0.173 | -0.329 | | -0.251 | |  |  |  |  |  |  |  |  |  |
| H15 | -0.173 | -0.329 | | -0.251 | |  |  |  |  |  |  |  |  |  |

**Table S17:** The reactivity parameter values by means of the condensed Fukui function for the stationary points of the P1 formation path proceed by the TS4-1WTB saddle point.

| **Atom** | **HO_2_+1WTB** | | | | | | **CR-TS4-1WTB** | | | **TS4-1WTB** | | | **CP-TS4-1WTB** | | |
| --- | --- | --- | --- | --- | --- | --- | --- | --- | --- | --- | --- | --- | --- | --- | --- |
|  | $f_{X}^{+}$ | | $f_{X}^{-}$ | | $f_{X}^{0}$ | | $f_{X}^{+}$ | $f_{X}^{-}$ | $f_{X}^{0}$ | $f_{X}^{+}$ | $f_{X}^{-}$ | $f_{X}^{0}$ | $f_{X}^{+}$ | $f_{X}^{-}$ | $f_{X}^{0}$ |
| C1 | -0.177 | | -0.109 | | -0.143 | | -0.079 | 0.007 | -0.036 | -0.039 | -0.059 | -0.049 | -0.040 | -0.061 | -0.050 |
| C2 | -0.108 | | -0.073 | | -0.090 | | -0.038 | -0.006 | -0.022 | -0.070 | -0.048 | -0.059 | -0.074 | -0.054 | -0.064 |
| C3 | -0.108 | | -0.073 | | -0.091 | | -0.097 | -0.013 | -0.055 | -0.036 | -0.051 | -0.044 | -0.037 | -0.050 | -0.043 |
| C4 | -0.177 | | -0.110 | | -0.143 | | -0.104 | -0.029 | -0.067 | -0.161 | -0.171 | -0.166 | -0.176 | -0.195 | -0.186 |
| S5 | -0.185 | | -0.267 | | -0.226 | | -0.143 | -0.049 | -0.096 | -0.302 | -0.205 | -0.254 | -0.312 | -0.211 | -0.261 |
| H6 | -0.066 | | -0.134 | | -0.100 | | -0.029 | -0.003 | -0.016 | -0.038 | -0.044 | -0.041 | -0.039 | -0.047 | -0.043 |
| H7 | -0.056 | | -0.049 | | -0.053 | | -0.031 | -0.010 | -0.020 | -0.034 | -0.029 | -0.032 | -0.038 | -0.034 | -0.036 |
| H8 | -0.056 | | -0.050 | | -0.053 | | -0.044 | -0.017 | -0.031 | -0.050 | -0.051 | -0.050 | -0.058 | -0.059 | -0.059 |
| H9 | -0.066 | | -0.135 | | -0.101 | | -0.045 | -0.023 | -0.034 | -0.060 | -0.069 | -0.064 | -0.065 | -0.079 | -0.072 |
| O10 | -0.387 | | -0.285 | | -0.336 | | -0.136 | -0.247 | -0.191 | -0.043 | -0.061 | -0.052 | -0.047 | -0.066 | -0.056 |
| H11 | -0.128 | | -0.132 | | -0.130 | | -0.025 | -0.074 | -0.050 | -0.027 | -0.040 | -0.034 | -0.032 | -0.043 | -0.038 |
| O12 | -0.486 | | -0.583 | | -0.535 | | -0.147 | -0.446 | -0.297 | -0.101 | -0.132 | -0.117 | -0.056 | -0.072 | -0.064 |
| O13 | -0.653 | | -0.342 | | -0.497 | | -0.041 | -0.030 | -0.036 | -0.014 | -0.012 | -0.013 | -0.013 | -0.012 | -0.012 |
| H14 | -0.173 | | -0.329 | | -0.251 | | -0.010 | -0.027 | -0.018 | -0.003 | -0.007 | -0.005 | 0.000 | -0.003 | -0.002 |
| H15 | -0.173 | | -0.329 | | -0.251 | | -0.032 | -0.033 | -0.032 | -0.020 | -0.021 | -0.021 | -0.014 | -0.014 | -0.014 |
| **Atom** | **P1+H_2_O** | | | | |  |  |  |  |  |  |  |  |  |  |
|  | $f_{X}^{+}$ | $f_{X}^{-}$ | | $f_{X}^{0}$ | |  |  |  |  |  |  |  |  |  |  |
| C1 | -0.183 | -0.203 | | -0.193 | |  |  |  |  |  |  |  |  |  |  |
| C2 | -0.036 | -0.049 | | -0.042 | |  |  |  |  |  |  |  |  |  |  |
| C3 | -0.074 | -0.059 | | -0.067 | |  |  |  |  |  |  |  |  |  |  |
| C4 | -0.041 | -0.065 | | -0.053 | |  |  |  |  |  |  |  |  |  |  |
| S5 | -0.313 | -0.209 | | -0.261 | |  |  |  |  |  |  |  |  |  |  |
| H6 | -0.066 | -0.082 | | -0.074 | |  |  |  |  |  |  |  |  |  |  |
| H7 | -0.057 | -0.059 | | -0.058 | |  |  |  |  |  |  |  |  |  |  |
| H8 | -0.046 | -0.043 | | -0.044 | |  |  |  |  |  |  |  |  |  |  |
| H9 | -0.039 | -0.049 | | -0.044 | |  |  |  |  |  |  |  |  |  |  |
| O10 | -0.047 | -0.062 | | -0.054 | |  |  |  |  |  |  |  |  |  |  |
| H11 | -0.032 | -0.042 | | -0.037 | |  |  |  |  |  |  |  |  |  |  |
| O12 | -0.065 | -0.079 | | -0.072 | |  |  |  |  |  |  |  |  |  |  |
| O13 | -0.653 | -0.342 | | -0.497 | |  |  |  |  |  |  |  |  |  |  |
| H14 | -0.173 | -0.329 | | -0.251 | |  |  |  |  |  |  |  |  |  |  |
| H15 | -0.173 | -0.329 | | -0.251 | |  |  |  |  |  |  |  |  |  |  |

**Table S18:** The reactivity parameter values by means of the condensed Fukui function for the stationary points of the P2 and P4 formation path started from the 1WHA and thiophene species.

| **Atom** | **Thio+1WHA** | | | **CR-TS2-1WHA** | | | **TS2-1WHA** | | | **CP-TS2-1WHA** | | |
| --- | --- | --- | --- | --- | --- | --- | --- | --- | --- | --- | --- | --- |
|  | $f_{X}^{+}$ | $f_{X}^{-}$ | $f_{X}^{0}$ | $f_{X}^{+}$ | $f_{X}^{-}$ | $f_{X}^{0}$ | $f_{X}^{+}$ | $f_{X}^{-}$ | $f_{X}^{0}$ | $f_{X}^{+}$ | $f_{X}^{-}$ | $f_{X}^{0}$ |
| C1 | -0.177 | -0.109 | -0.143 | -0.079 | -0.004 | -0.041 | -0.053 | -0.028 | -0.040 | -0.030 | -0.035 | -0.032 |
| C2 | -0.108 | -0.073 | -0.090 | -0.050 | -0.022 | -0.036 | -0.102 | -0.116 | -0.109 | -0.145 | -0.159 | -0.152 |
| C3 | -0.108 | -0.073 | -0.091 | -0.069 | -0.029 | -0.049 | -0.064 | -0.053 | -0.058 | -0.066 | -0.067 | -0.066 |
| C4 | -0.177 | -0.110 | -0.143 | -0.108 | -0.045 | -0.076 | -0.134 | -0.128 | -0.131 | -0.165 | -0.177 | -0.171 |
| S5 | -0.185 | -0.267 | -0.226 | -0.105 | -0.060 | -0.083 | -0.170 | -0.144 | -0.157 | -0.242 | -0.170 | -0.206 |
| H6 | -0.066 | -0.134 | -0.100 | -0.023 | -0.011 | -0.017 | -0.040 | -0.039 | -0.040 | -0.046 | -0.045 | -0.046 |
| H7 | -0.056 | -0.049 | -0.053 | -0.029 | -0.014 | -0.021 | -0.045 | -0.051 | -0.048 | -0.058 | -0.068 | -0.063 |
| H8 | -0.056 | -0.050 | -0.053 | -0.037 | -0.023 | -0.030 | -0.042 | -0.040 | -0.041 | -0.045 | -0.046 | -0.045 |
| H9 | -0.066 | -0.135 | -0.101 | -0.044 | -0.028 | -0.036 | -0.055 | -0.061 | -0.058 | -0.065 | -0.079 | -0.072 |
| O10 | -0.387 | -0.285 | -0.336 | -0.188 | -0.239 | -0.214 | -0.112 | -0.102 | -0.107 | -0.056 | -0.052 | -0.054 |
| H11 | -0.128 | -0.132 | -0.130 | -0.038 | -0.066 | -0.052 | -0.034 | -0.036 | -0.035 | -0.019 | -0.020 | -0.020 |
| O12 | -0.486 | -0.583 | -0.535 | -0.188 | -0.418 | -0.303 | -0.113 | -0.154 | -0.134 | -0.041 | -0.040 | -0.040 |
| O13 | -0.653 | -0.342 | -0.497 | -0.016 | -0.007 | -0.012 | -0.013 | -0.015 | -0.014 | -0.007 | -0.012 | -0.009 |
| H14 | -0.173 | -0.329 | -0.251 | -0.021 | -0.026 | -0.024 | -0.024 | -0.026 | -0.025 | -0.019 | -0.025 | -0.022 |
| H15 | -0.173 | -0.329 | -0.251 | -0.005 | -0.008 | -0.007 | 0.001 | -0.007 | -0.003 | 0.003 | -0.006 | -0.001 |
| **Atom** | **P2+H_2_O** | | | **IN8a-1WHA** | | | **TS8a-1WHA** | | | **CP-TS8a-1WHA** | | |
|  | $f_{X}^{+}$ | $f_{X}^{-}$ | $f_{X}^{0}$ | $f_{X}^{+}$ | $f_{X}^{-}$ | $f_{X}^{0}$ | $f_{X}^{+}$ | $f_{X}^{-}$ | $f_{X}^{0}$ | $f_{X}^{+}$ | $f_{X}^{-}$ | $f_{X}^{0}$ |
| C1 | -0.031 | -0.036 | -0.034 | -0.030 | -0.033 | -0.032 | -0.030 | -0.026 | -0.028 | -0.037 | -0.026 | -0.032 |
| C2 | -0.146 | -0.164 | -0.155 | -0.148 | -0.167 | -0.158 | -0.067 | -0.126 | -0.097 | -0.049 | -0.048 | -0.048 |
| C3 | -0.067 | -0.070 | -0.068 | -0.066 | -0.068 | -0.067 | -0.068 | -0.064 | -0.066 | -0.037 | -0.039 | -0.038 |
| C4 | -0.165 | -0.177 | -0.171 | -0.164 | -0.174 | -0.169 | -0.091 | -0.129 | -0.110 | -0.064 | -0.057 | -0.061 |
| S5 | -0.244 | -0.172 | -0.208 | -0.222 | -0.146 | -0.184 | -0.170 | -0.185 | -0.178 | -0.316 | -0.120 | -0.218 |
| H6 | -0.046 | -0.046 | -0.046 | -0.045 | -0.044 | -0.045 | -0.046 | -0.038 | -0.042 | -0.017 | -0.011 | -0.014 |
| H7 | -0.060 | -0.072 | -0.066 | -0.061 | -0.074 | -0.067 | -0.039 | -0.058 | -0.049 | -0.037 | -0.034 | -0.035 |
| H8 | -0.046 | -0.047 | -0.046 | -0.045 | -0.046 | -0.045 | -0.042 | -0.044 | -0.043 | -0.038 | -0.031 | -0.034 |
| H9 | -0.065 | -0.079 | -0.072 | -0.064 | -0.077 | -0.070 | -0.045 | -0.059 | -0.052 | -0.052 | -0.033 | -0.042 |
| O10 | -0.058 | -0.055 | -0.057 | -0.055 | -0.062 | -0.059 | -0.181 | -0.158 | -0.169 | -0.117 | -0.408 | -0.262 |
| H11 | -0.028 | -0.036 | -0.032 | -0.017 | -0.020 | -0.019 | -0.053 | -0.051 | -0.052 | -0.022 | -0.093 | -0.058 |
| O12 | -0.044 | -0.047 | -0.045 | -0.038 | -0.037 | -0.038 | -0.130 | -0.029 | -0.079 | -0.099 | -0.051 | -0.075 |
| O13 | -0.653 | -0.342 | -0.497 | -0.021 | -0.020 | -0.020 | -0.018 | -0.012 | -0.015 | -0.064 | -0.009 | -0.037 |
| H14 | -0.173 | -0.329 | -0.251 | -0.006 | -0.013 | -0.010 | 0.001 | 0.000 | 0.001 | -0.016 | -0.011 | -0.014 |
| H15 | -0.173 | -0.329 | -0.251 | -0.018 | -0.019 | -0.018 | -0.022 | -0.020 | -0.021 | -0.034 | -0.030 | -0.032 |
| **Atom** | **P4+H_2_O** | | |  |  |  |  |  |  |  |  |  |
|  | $f_{X}^{+}$ | $f_{X}^{-}$ | $f_{X}^{0}$ |  |  |  |  |  |  |  |  |  |
| C1 | -0.047 | -0.064 | -0.056 |  |  |  |  |  |  |  |  |  |
| C2 | -0.068 | -0.108 | -0.088 |  |  |  |  |  |  |  |  |  |
| C3 | -0.039 | -0.070 | -0.055 |  |  |  |  |  |  |  |  |  |
| C4 | -0.080 | -0.120 | -0.100 |  |  |  |  |  |  |  |  |  |
| S5 | -0.438 | -0.330 | -0.384 |  |  |  |  |  |  |  |  |  |
| H6 | -0.034 | -0.026 | -0.030 |  |  |  |  |  |  |  |  |  |
| H7 | -0.043 | -0.054 | -0.049 |  |  |  |  |  |  |  |  |  |
| H8 | -0.044 | -0.049 | -0.047 |  |  |  |  |  |  |  |  |  |
| H9 | -0.065 | -0.059 | -0.062 |  |  |  |  |  |  |  |  |  |
| O10 | -0.142 | -0.119 | -0.130 |  |  |  |  |  |  |  |  |  |
| H11 | -0.199 | -0.243 | -0.221 |  |  |  |  |  |  |  |  |  |
| O12 | -0.801 | -0.757 | -0.779 |  |  |  |  |  |  |  |  |  |
| O13 | -0.653 | -0.342 | -0.497 |  |  |  |  |  |  |  |  |  |
| H14 | -0.173 | -0.329 | -0.251 |  |  |  |  |  |  |  |  |  |
| H15 | -0.173 | -0.329 | -0.251 |  |  |  |  |  |  |  |  |  |

**Table S19:** The reactivity parameter values by means of the condensed Fukui function for the stationary points of the P2 and P4 formation path started from the HO_2_ and 1WTA species.

| **Atom** | **HO_2_+1WTA** | | | **CR-TS2-1WTA** | | | **TS2-1WTA** | | | **CP-TS2-1WTA** | | |
| --- | --- | --- | --- | --- | --- | --- | --- | --- | --- | --- | --- | --- |
|  | $f_{X}^{+}$ | $f_{X}^{-}$ | $f_{X}^{0}$ | $f_{X}^{+}$ | $f_{X}^{-}$ | $f_{X}^{0}$ | $f_{X}^{+}$ | $f_{X}^{-}$ | $f_{X}^{0}$ | $f_{X}^{+}$ | $f_{X}^{-}$ | $f_{X}^{0}$ |
| C1 | -0.177 | -0.109 | -0.143 | -0.094 | 0.001 | -0.047 | -0.058 | -0.029 | -0.043 | -0.030 | -0.036 | -0.033 |
| C2 | -0.108 | -0.073 | -0.090 | -0.052 | -0.004 | -0.028 | -0.101 | -0.117 | -0.109 | -0.143 | -0.161 | -0.152 |
| C3 | -0.108 | -0.073 | -0.091 | -0.072 | -0.012 | -0.042 | -0.067 | -0.054 | -0.061 | -0.067 | -0.068 | -0.068 |
| C4 | -0.177 | -0.110 | -0.143 | -0.121 | -0.035 | -0.078 | -0.134 | -0.126 | -0.130 | -0.163 | -0.174 | -0.169 |
| S5 | -0.185 | -0.267 | -0.226 | -0.128 | -0.064 | -0.096 | -0.172 | -0.146 | -0.159 | -0.240 | -0.168 | -0.204 |
| H6 | -0.066 | -0.134 | -0.100 | -0.032 | -0.006 | -0.019 | -0.032 | -0.031 | -0.032 | -0.038 | -0.037 | -0.038 |
| H7 | -0.056 | -0.049 | -0.053 | -0.032 | -0.010 | -0.021 | -0.046 | -0.053 | -0.050 | -0.058 | -0.070 | -0.064 |
| H8 | -0.056 | -0.050 | -0.053 | -0.040 | -0.015 | -0.028 | -0.043 | -0.041 | -0.042 | -0.045 | -0.046 | -0.046 |
| H9 | -0.066 | -0.135 | -0.101 | -0.049 | -0.024 | -0.037 | -0.055 | -0.060 | -0.058 | -0.064 | -0.077 | -0.071 |
| O10 | -0.387 | -0.285 | -0.336 | -0.145 | -0.213 | -0.179 | -0.097 | -0.088 | -0.092 | -0.054 | -0.053 | -0.053 |
| H11 | -0.128 | -0.132 | -0.130 | -0.029 | -0.068 | -0.049 | -0.040 | -0.046 | -0.043 | -0.025 | -0.035 | -0.030 |
| O12 | -0.486 | -0.583 | -0.535 | -0.169 | -0.491 | -0.330 | -0.118 | -0.173 | -0.145 | -0.044 | -0.048 | -0.046 |
| O13 | -0.653 | -0.342 | -0.497 | -0.009 | -0.017 | -0.013 | -0.012 | -0.009 | -0.011 | -0.009 | -0.005 | -0.007 |
| H14 | -0.173 | -0.329 | -0.251 | -0.002 | -0.011 | -0.006 | -0.002 | -0.005 | -0.003 | 0.000 | -0.002 | -0.001 |
| H15 | -0.173 | -0.329 | -0.251 | -0.024 | -0.031 | -0.027 | -0.022 | -0.022 | -0.022 | -0.019 | -0.019 | -0.019 |
| **Atom** | **P2+H_2_O** | | | **IN8a-1WTA** | | | **TS8a-1WTA** | | | **CP-TS8a-1WTA** | | |
|  | $f_{X}^{+}$ | $f_{X}^{-}$ | $f_{X}^{0}$ | $f_{X}^{+}$ | $f_{X}^{-}$ | $f_{X}^{0}$ | $f_{X}^{+}$ | $f_{X}^{-}$ | $f_{X}^{0}$ | $f_{X}^{+}$ | $f_{X}^{-}$ | $f_{X}^{0}$ |
| C1 | -0.031 | -0.036 | -0.034 | -0.032 | -0.035 | -0.033 | -0.030 | -0.026 | -0.028 | -0.058 | -0.038 | -0.048 |
| C2 | -0.146 | -0.164 | -0.155 | -0.146 | -0.158 | -0.152 | -0.069 | -0.122 | -0.095 | -0.037 | -0.036 | -0.036 |
| C3 | -0.067 | -0.070 | -0.068 | -0.067 | -0.069 | -0.068 | -0.067 | -0.064 | -0.066 | -0.053 | -0.051 | -0.052 |
| C4 | -0.165 | -0.177 | -0.171 | -0.163 | -0.165 | -0.164 | -0.093 | -0.126 | -0.110 | -0.050 | -0.048 | -0.049 |
| S5 | -0.244 | -0.172 | -0.208 | -0.243 | -0.167 | -0.205 | -0.176 | -0.190 | -0.183 | -0.255 | -0.143 | -0.199 |
| H6 | -0.046 | -0.046 | -0.046 | -0.040 | -0.039 | -0.039 | -0.036 | -0.033 | -0.035 | -0.034 | -0.012 | -0.023 |
| H7 | -0.060 | -0.072 | -0.066 | -0.060 | -0.071 | -0.066 | -0.038 | -0.056 | -0.047 | -0.034 | -0.029 | -0.032 |
| H8 | -0.046 | -0.047 | -0.046 | -0.045 | -0.047 | -0.046 | -0.041 | -0.044 | -0.043 | -0.042 | -0.032 | -0.037 |
| H9 | -0.065 | -0.079 | -0.072 | -0.064 | -0.074 | -0.069 | -0.045 | -0.058 | -0.052 | -0.044 | -0.032 | -0.038 |
| O10 | -0.058 | -0.055 | -0.057 | -0.051 | -0.066 | -0.058 | -0.156 | -0.132 | -0.144 | -0.082 | -0.354 | -0.218 |
| H11 | -0.028 | -0.036 | -0.032 | -0.027 | -0.037 | -0.032 | -0.061 | -0.058 | -0.060 | -0.034 | -0.114 | -0.074 |
| O12 | -0.044 | -0.047 | -0.045 | -0.039 | -0.049 | -0.044 | -0.127 | -0.046 | -0.086 | -0.151 | -0.041 | -0.096 |
| O13 | -0.653 | -0.342 | -0.497 | -0.004 | -0.002 | -0.003 | -0.027 | -0.013 | -0.020 | -0.073 | -0.024 | -0.049 |
| H14 | -0.173 | -0.329 | -0.251 | -0.001 | -0.003 | -0.002 | -0.006 | -0.009 | -0.008 | -0.017 | -0.021 | -0.019 |
| H15 | -0.173 | -0.329 | -0.251 | -0.018 | -0.018 | -0.018 | -0.027 | -0.022 | -0.024 | -0.037 | -0.026 | -0.032 |
| **Atom** | **P4++H_2_O** | | |  |  |  |  |  |  |  |  |  |
|  | $f_{X}^{+}$ | $f_{X}^{-}$ | $f_{X}^{0}$ |  |  |  |  |  |  |  |  |  |
| C1 | -0.047 | -0.064 | -0.056 |  |  |  |  |  |  |  |  |  |
| C2 | -0.068 | -0.108 | -0.088 |  |  |  |  |  |  |  |  |  |
| C3 | -0.039 | -0.070 | -0.055 |  |  |  |  |  |  |  |  |  |
| C4 | -0.080 | -0.120 | -0.100 |  |  |  |  |  |  |  |  |  |
| S5 | -0.438 | -0.330 | -0.384 |  |  |  |  |  |  |  |  |  |
| H6 | -0.034 | -0.026 | -0.030 |  |  |  |  |  |  |  |  |  |
| H7 | -0.043 | -0.054 | -0.049 |  |  |  |  |  |  |  |  |  |
| H8 | -0.044 | -0.049 | -0.047 |  |  |  |  |  |  |  |  |  |
| H9 | -0.065 | -0.059 | -0.062 |  |  |  |  |  |  |  |  |  |
| O10 | -0.142 | -0.119 | -0.130 |  |  |  |  |  |  |  |  |  |
| H11 | -0.199 | -0.243 | -0.221 |  |  |  |  |  |  |  |  |  |
| O12 | -0.801 | -0.757 | -0.779 |  |  |  |  |  |  |  |  |  |
| O13 | -0.653 | -0.342 | -0.497 |  |  |  |  |  |  |  |  |  |
| H14 | -0.173 | -0.329 | -0.251 |  |  |  |  |  |  |  |  |  |
| H15 | -0.173 | -0.329 | -0.251 |  |  |  |  |  |  |  |  |  |

**Table S20:** The reactivity parameter values by means of the condensed Fukui function for the stationary points of the P3 formation path started from the thiophene and 1WHA species.

| **Atom** | **Thio+1WHA** | | | **CR-TS3-1WHA** | | | **TS3-1WHA** | | | **CP-TS3-1WHA** | | |
| --- | --- | --- | --- | --- | --- | --- | --- | --- | --- | --- | --- | --- |
|  | $f_{X}^{+}$ | $f_{X}^{-}$ | $f_{X}^{0}$ | $f_{X}^{+}$ | $f_{X}^{-}$ | $f_{X}^{0}$ | $f_{X}^{+}$ | $f_{X}^{-}$ | $f_{X}^{0}$ | $f_{X}^{+}$ | $f_{X}^{-}$ | $f_{X}^{0}$ |
| C1 | -0.177 | -0.109 | -0.143 | -0.103 | -0.042 | -0.072 | -0.039 | -0.071 | -0.055 | -0.114 | -0.030 | -0.072 |
| C2 | -0.108 | -0.073 | -0.090 | -0.066 | -0.017 | -0.041 | -0.078 | -0.090 | -0.084 | -0.104 | -0.041 | -0.073 |
| C3 | -0.108 | -0.073 | -0.091 | -0.033 | -0.003 | -0.018 | -0.079 | -0.089 | -0.084 | -0.054 | -0.030 | -0.042 |
| C4 | -0.177 | -0.110 | -0.143 | -0.086 | -0.022 | -0.054 | -0.037 | -0.069 | -0.053 | -0.111 | -0.060 | -0.086 |
| S5 | -0.185 | -0.267 | -0.226 | -0.122 | -0.088 | -0.105 | -0.196 | -0.170 | -0.183 | -0.113 | -0.075 | -0.094 |
| H6 | -0.066 | -0.134 | -0.100 | -0.043 | -0.027 | -0.035 | -0.033 | -0.043 | -0.038 | -0.046 | -0.028 | -0.037 |
| H7 | -0.056 | -0.049 | -0.053 | -0.033 | -0.015 | -0.024 | -0.043 | -0.051 | -0.047 | -0.045 | -0.027 | -0.036 |
| H8 | -0.056 | -0.050 | -0.053 | -0.016 | -0.007 | -0.012 | -0.043 | -0.051 | -0.047 | -0.042 | -0.028 | -0.035 |
| H9 | -0.066 | -0.135 | -0.101 | -0.034 | -0.015 | -0.025 | -0.030 | -0.039 | -0.034 | -0.049 | -0.036 | -0.042 |
| O10 | -0.387 | -0.285 | -0.336 | -0.194 | -0.422 | -0.308 | -0.127 | -0.105 | -0.116 | -0.108 | -0.042 | -0.075 |
| H11 | -0.128 | -0.132 | -0.130 | -0.190 | -0.237 | -0.214 | -0.194 | -0.128 | -0.161 | -0.134 | -0.445 | -0.290 |
| O12 | -0.486 | -0.583 | -0.535 | -0.038 | -0.066 | -0.052 | -0.060 | -0.044 | -0.052 | -0.027 | -0.106 | -0.066 |
| O13 | -0.653 | -0.342 | -0.497 | -0.016 | -0.007 | -0.011 | -0.015 | -0.013 | -0.014 | -0.020 | -0.009 | -0.014 |
| H14 | -0.173 | -0.329 | -0.251 | -0.021 | -0.026 | -0.023 | -0.025 | -0.026 | -0.025 | -0.026 | -0.030 | -0.028 |
| H15 | -0.173 | -0.329 | -0.251 | -0.004 | -0.007 | -0.006 | -0.001 | -0.011 | -0.006 | -0.007 | -0.013 | -0.010 |
| **Atom** | **P3+H_2_O** | | |  |  |  |  |  |  |  |  |  |
|  | $f_{X}^{+}$ | $f_{X}^{-}$ | $f_{X}^{0}$ |  |  |  |  |  |  |  |  |  |
| C1 | -0.049 | -0.120 | -0.084 |  |  |  |  |  |  |  |  |  |
| C2 | -0.078 | -0.108 | -0.093 |  |  |  |  |  |  |  |  |  |
| C3 | -0.078 | -0.108 | -0.093 |  |  |  |  |  |  |  |  |  |
| C4 | -0.049 | -0.120 | -0.084 |  |  |  |  |  |  |  |  |  |
| S5 | -0.238 | -0.174 | -0.206 |  |  |  |  |  |  |  |  |  |
| H6 | -0.038 | -0.062 | -0.050 |  |  |  |  |  |  |  |  |  |
| H7 | -0.052 | -0.062 | -0.057 |  |  |  |  |  |  |  |  |  |
| H8 | -0.052 | -0.062 | -0.057 |  |  |  |  |  |  |  |  |  |
| H9 | -0.038 | -0.062 | -0.050 |  |  |  |  |  |  |  |  |  |
| O10 | -0.326 | -0.123 | -0.224 |  |  |  |  |  |  |  |  |  |
| O11 | -0.199 | -0.243 | -0.221 |  |  |  |  |  |  |  |  |  |
| H12 | -0.801 | -0.757 | -0.779 |  |  |  |  |  |  |  |  |  |
| O13 | -0.653 | -0.342 | -0.497 |  |  |  |  |  |  |  |  |  |
| H14 | -0.173 | -0.329 | -0.251 |  |  |  |  |  |  |  |  |  |
| H15 | -0.173 | -0.329 | -0.251 |  |  |  |  |  |  |  |  |  |

**Table S21:** The reactivity parameter values by means of the condensed Fukui function for the stationary points of the P3 formation path started from the HO_2_ + 1WTA species.

| **Atom** | **HO_2_+1WTA** | | | **CR-TS2-1WTA** | | | **TS3-1WTA** | | | **CP-TS3-1WTA** | | |
| --- | --- | --- | --- | --- | --- | --- | --- | --- | --- | --- | --- | --- |
|  | $f_{X}^{+}$ | $f_{X}^{-}$ | $f_{X}^{0}$ | $f_{X}^{+}$ | $f_{X}^{-}$ | $f_{X}^{0}$ | $f_{X}^{+}$ | $f_{X}^{-}$ | $f_{X}^{0}$ | $f_{X}^{+}$ | $f_{X}^{-}$ | $f_{X}^{0}$ |
| C1 | -0.177 | -0.109 | -0.143 | -0.094 | 0.001 | -0.047 | -0.036 | -0.066 | -0.051 | -0.064 | -0.006 | -0.035 |
| C2 | -0.108 | -0.073 | -0.090 | -0.052 | -0.004 | -0.028 | -0.068 | -0.076 | -0.072 | -0.061 | -0.039 | -0.050 |
| C3 | -0.108 | -0.073 | -0.091 | -0.072 | -0.012 | -0.042 | -0.093 | -0.096 | -0.094 | -0.083 | -0.038 | -0.061 |
| C4 | -0.177 | -0.110 | -0.143 | -0.121 | -0.035 | -0.078 | -0.041 | -0.067 | -0.054 | -0.069 | -0.034 | -0.052 |
| S5 | -0.185 | -0.267 | -0.226 | -0.128 | -0.064 | -0.096 | -0.201 | -0.177 | -0.189 | -0.145 | -0.040 | -0.093 |
| H6 | -0.066 | -0.134 | -0.100 | -0.032 | -0.006 | -0.019 | -0.025 | -0.033 | -0.029 | -0.026 | -0.005 | -0.016 |
| H7 | -0.056 | -0.049 | -0.053 | -0.032 | -0.010 | -0.021 | -0.041 | -0.047 | -0.044 | -0.044 | -0.025 | -0.034 |
| H8 | -0.056 | -0.050 | -0.053 | -0.040 | -0.015 | -0.028 | -0.047 | -0.054 | -0.050 | -0.045 | -0.027 | -0.036 |
| H9 | -0.066 | -0.135 | -0.101 | -0.049 | -0.024 | -0.037 | -0.034 | -0.043 | -0.039 | -0.039 | -0.023 | -0.031 |
| O10 | -0.387 | -0.285 | -0.336 | -0.145 | -0.213 | -0.179 | -0.132 | -0.138 | -0.135 | -0.167 | 0.017 | -0.075 |
| H11 | -0.128 | -0.132 | -0.130 | -0.029 | -0.068 | -0.049 | -0.178 | -0.119 | -0.149 | -0.083 | -0.544 | -0.314 |
| O12 | -0.486 | -0.583 | -0.535 | -0.169 | -0.491 | -0.330 | -0.061 | -0.048 | -0.054 | -0.012 | -0.124 | -0.068 |
| O13 | -0.653 | -0.342 | -0.497 | -0.009 | -0.017 | -0.013 | -0.018 | -0.010 | -0.014 | -0.097 | -0.036 | -0.067 |
| H14 | -0.173 | -0.329 | -0.251 | -0.002 | -0.011 | -0.006 | -0.002 | -0.005 | -0.004 | -0.021 | -0.037 | -0.029 |
| H15 | -0.173 | -0.329 | -0.251 | -0.024 | -0.031 | -0.027 | -0.023 | -0.022 | -0.022 | -0.046 | -0.036 | -0.041 |
| **Atom** | **P3+H_2_O** | | |  |  |  |  |  |  |  |  |  |
|  | $f_{X}^{+}$ | $f_{X}^{-}$ | $f_{X}^{0}$ |  |  |  |  |  |  |  |  |  |
| C1 | -0.049 | -0.120 | -0.084 |  |  |  |  |  |  |  |  |  |
| C2 | -0.078 | -0.108 | -0.093 |  |  |  |  |  |  |  |  |  |
| C3 | -0.078 | -0.108 | -0.093 |  |  |  |  |  |  |  |  |  |
| C4 | -0.049 | -0.120 | -0.084 |  |  |  |  |  |  |  |  |  |
| S5 | -0.238 | -0.174 | -0.206 |  |  |  |  |  |  |  |  |  |
| H6 | -0.038 | -0.062 | -0.050 |  |  |  |  |  |  |  |  |  |
| H7 | -0.052 | -0.062 | -0.057 |  |  |  |  |  |  |  |  |  |
| H8 | -0.052 | -0.062 | -0.057 |  |  |  |  |  |  |  |  |  |
| H9 | -0.038 | -0.062 | -0.050 |  |  |  |  |  |  |  |  |  |
| O10 | -0.326 | -0.123 | -0.224 |  |  |  |  |  |  |  |  |  |
| H11 | -0.199 | -0.243 | -0.221 |  |  |  |  |  |  |  |  |  |
| O12 | -0.801 | -0.757 | -0.779 |  |  |  |  |  |  |  |  |  |
| O13 | -0.653 | -0.342 | -0.497 |  |  |  |  |  |  |  |  |  |
| H14 | -0.173 | -0.329 | -0.251 |  |  |  |  |  |  |  |  |  |
| H15 | -0.173 | -0.329 | -0.251 |  |  |  |  |  |  |  |  |  |

**Table S22**. Optimized cartesian Coordinates of all stationary points of thiophene and hydroperoxyl radical reaction in the presence and absence of water molecule.

| Thiophene | C -0.01034100 -1.24339100 0.00030600  C -1.27454400 -0.71515600 0.00024900  C -1.27460100 0.71526700 -0.00070000  C -0.01008400 1.24338900 0.00063200  S 1.19996000 -0.00004800 -0.00026500  H 0.28207300 -2.28434800 0.00036100  H -2.17300600 -1.32180200 0.00008900  H -2.17322000 1.32179100 -0.00062800  H 0.28221000 2.28447000 0.00149700 |
| --- | --- |
| OH | O 0.00000000 0.00000000 0.10884400  H 0.00000000 0.00000000 -0.87075000 |
| HO_2_ | O 0.05556600 0.72150300 0.00000000  O 0.05556600 -0.61225300 0.00000000  H -0.88904900 -0.87399600 0.00000000 |
| H_2_O | O 0.00000000 0.11648200 0.00000000  H 0.76947600 -0.46591500 0.00000000  H -0.76947600 -0.46593800 0.00000000 |
| CW | O -1.21405100 0.63691400 0.02176100  O -0.93438900 -0.66678500 -0.00264200  H 0.06142700 -0.69107600 -0.01947800  O 1.66919200 0.01251900 -0.08345100  H 1.43013700 0.95035700 -0.06138700  H 2.34242300 -0.12046600 0.59551900 |
| 1WHA | O 1.21463200 0.63671400 0.02180500  O 0.93404500 -0.66682700 -0.00269100  H -0.06182100 -0.69035500 -0.01954600  O -1.66926600 0.01264800 -0.08341400  H -1.43107900 0.95071100 -0.06166400  H -2.34238600 -0.12063300 0.59561300 |
| 1WHB | O 0.79922100 0.64251700 0.00044400  O 1.49476400 -0.49366400 0.00009600  H 2.43666600 -0.22131400 -0.00310600  O -2.16748900 -0.02090000 -0.00067300  H -1.21880200 0.18103500 0.00120000  H -2.22983200 -0.98334900 0.00297000 |
| 1WHC | O -0.77260300 0.38717600 0.00331200  H -1.12976800 1.29981200 -0.00779800  O -1.82731300 -0.42874000 -0.00155600  O 2.25301000 0.00289500 -0.00145200  H 2.61382000 -0.89162600 0.00617500  H 1.29120100 -0.09883700 -0.00080900 |
| 1WTA | C 0.32634400 0.35115100 -0.00303500  C -0.54905100 1.40585300 -0.00129700  C -1.91936600 0.99381000 0.00165600  C -2.06073000 -0.36936700 0.00210900  S -0.51827500 -1.16606600 -0.00106800  H 1.40923100 0.36458100 -0.00535300  H -0.22798500 2.44154800 -0.00213200  H -2.75939300 1.67952000 0.00334200  H -2.97334300 -0.94950900 0.00410500  O 3.69567700 0.11973400 0.00082600  H 4.23965600 -0.06110500 0.77750000  H 4.25564000 -0.06453800 -0.76357300 |
| 1WTB | C 0.00557900 -0.37251600 1.05837800  C -0.13683900 0.98131600 0.88213100  C 0.64993200 1.48136400 -0.20369700  C 1.37527000 0.49903200 -0.82532500  S 1.10843500 -1.05303000 -0.09653600  H -0.45793400 -1.00425300 1.80407100  H -0.77299700 1.59922300 1.50592800  H 0.67310600 2.52205700 -0.50577100  H 2.04744700 0.59330200 -1.66724300  O -3.15622400 -0.22791000 -0.44233900  H -3.09519000 -0.49445600 -1.36769900  H -2.24325200 -0.07930100 -0.15490700 |
| 1WTC | C -0.27856300 -1.03139300 -0.00003100  C 0.53578400 0.07087900 -0.00016800  C -0.20655100 1.29479100 -0.00009900  C -1.56279500 1.09784300 0.00009200  S -1.95907200 -0.59209200 0.00003800  H 0.00687200 -2.07455200 -0.00002600  H 1.61916300 0.01509200 -0.00028500  H 0.25088200 2.27790200 -0.00015600  H -2.34986700 1.83950400 0.00019900  O 3.97339200 -0.10530100 0.00002400  H 4.55144400 -0.16708300 0.77067100  H 4.55226600 -0.16770400 -0.76995500 |
| 1WTD | C 0.70015000 -0.12877000 -1.24522500  C 1.35923500 0.94848800 -0.71672800  C 1.35895600 0.94974800 0.71537600  C 0.69967100 -0.12658200 1.24551300  S 0.07206600 -1.16574800 0.00092800  H 0.54740900 -0.37998600 -2.28583100  H 1.82934300 1.71421000 -1.32327000  H 1.82884200 1.71653700 1.32074300  H 0.54652700 -0.37598400 2.28649700  O -3.17285900 0.57508400 -0.00070400  H -2.37194900 0.03165300 -0.00034700  H -2.85843700 1.48755500 -0.00062000 |
| CR1 | C 1.98449400 0.35436900 -0.63752300  C 1.32853400 1.43000800 -0.09837800  C 0.27822500 1.04154600 0.79247300  C 0.15666300 -0.32360800 0.90538500  S 1.33157500 -1.14861800 -0.06967900  H 2.80834100 0.35568800 -1.33815600  H 1.58166500 2.45914800 -0.32502800  H -0.34575100 1.74029600 1.33904600  H -0.53480300 -0.88463400 1.52016700  O -3.21064600 -0.28911400 0.17090800  O -2.50474900 0.21590700 -0.84025700  H -1.57897800 0.27914800 -0.49812100 |
| CR-TS1-1WHA | C 0.49433500 -0.41774300 0.59962700  C 1.01790800 0.73958800 1.12447200  C 2.30511400 1.06010700 0.58705300  C 2.73579500 0.13844100 -0.33103800  S 1.57715500 -1.13400500 -0.55435900  H -0.44978500 -0.89554600 0.83316000  H 0.51035100 1.33120600 1.87856900  H 2.88168200 1.93216200 0.87345000  H 3.66283600 0.13129400 -0.88805800  O -3.64597000 -0.07710600 -0.04043600  H -2.95441300 0.53326800 -0.43051200  O -2.95392800 -0.97255100 0.66332000  O -1.64063300 1.46386700 -0.97329900  H -1.46061400 1.52537200 -1.92005100  H -0.81920000 1.15028800 -0.55416100 |
| CR-TS1-1WHB | C 0.97180500 0.57272000 -0.14988200  C 1.83280400 0.70233600 -1.20846600  C 3.05173300 -0.02638000 -1.03089800  C 3.09579100 -0.69869100 0.16259100  S 1.64569800 -0.44715100 1.08387800  H -0.01132400 1.00758500 -0.01131800  H 1.60547300 1.29846200 -2.08523300  H 3.85724100 -0.04804700 -1.75667100  H 3.88907200 -1.32120900 0.55337300  O -3.64656400 -1.19362700 -0.72997500  H -4.27260800 -1.94113700 -0.83521100  O -4.23028000 -0.36327000 0.13020800  O -2.10698500 1.72428300 0.44115900  H -2.39063700 2.41651700 1.04985200  H -2.85056200 1.10324500 0.37195400 |
| CR-TS1-1WTA | C 0.94864300 -0.19050100 -1.03059000  C 0.38906500 0.84856200 -0.32631100  C 1.22458700 1.29276400 0.74843100  C 2.39736500 0.58880900 0.83317700  S 2.50423800 -0.63071400 -0.39660300  H 0.55317100 -0.69886700 -1.89995900  H -0.57825900 1.27774600 -0.56649100  H 0.96385700 2.10167300 1.42125900  H 3.20445000 0.71399700 1.54219800  O -3.78042100 -0.10294100 -0.00486100  H -3.02929800 -0.67949000 0.31982100  O -3.18495600 0.97525700 -0.51469700  O -1.61664400 -1.51134100 0.77314600  H -0.83100300 -1.03581300 0.44894900  H -1.45250900 -1.71343500 1.70292400 |
| CR-TS1-1WTA2 | C 0.83428400 1.32348500 0.11028900  C 0.85208100 0.51684200 1.22432600  C -0.36039500 -0.22685800 1.38302500  C -1.27735800 0.03203900 0.39736300  S -0.67213500 1.19047600 -0.74415200  H 1.59667000 2.01276300 -0.22802500  H 1.69452800 0.46351800 1.90522000  H -0.53987800 -0.92469800 2.19274100  H -2.26644400 -0.38894000 0.26171100  O 2.71946300 -1.16737500 -0.85536400  H 2.02854800 -0.56943800 -0.47487800  O 3.79560000 -0.98101900 -0.09156700  O -4.31316300 -1.18869600 -0.25852500  H -5.17755800 -0.85579100 0.01344200  H -4.48857900 -1.96137100 -0.81014000 |
| CR-TS1-1WTB | C -0.68415900 0.15222200 0.98881900  C -0.29986800 -1.03188600 0.40239900  C -1.20960500 -1.45468300 -0.61881000  C -2.26360400 -0.59359700 -0.77866000  S -2.16459800 0.75371600 0.30912900  H -0.20763900 0.68206200 1.80329500  H 0.59470200 -1.57735300 0.68648800  H -1.08071400 -2.35703500 -1.20510800  H -3.08728600 -0.66545800 -1.47591500  O 1.96139800 0.93136300 -0.74617000  H 1.08768900 0.60476500 -0.40883400  O 2.14729400 2.12730800 -0.18474700  O 3.01712900 -1.60644500 0.34727200  H 3.18699400 -0.77022500 -0.11056300  H 3.87667700 -2.02636200 0.47123900 |
| CR-TS2-1WHA | C 0.49346200 -0.41598400 0.59669200  C 1.01701400 0.74074800 1.12288600  C 2.30586200 1.05988600 0.58865300  C 2.73774800 0.13783100 -0.32849200  S 1.57829600 -1.13332200 -0.55476000  H -0.45186300 -0.89264200 0.82775600  H 0.50817300 1.33295600 1.87565500  H 2.88273700 1.93124500 0.87655000  H 3.66619000 0.12969500 -0.88315900  O -3.64675400 -0.07911300 -0.03895300  H -2.95669700 0.53070500 -0.43257800  O -2.95231700 -0.97118700 0.66665800  O -1.64346000 1.46191900 -0.97597400  H -1.46373300 1.52566800 -1.92262800  H -0.82181500 1.14768800 -0.55772200 |
| CR-TS2-1WHB | C 0.38373600 -0.75690500 0.44250600  C 0.78620700 0.20863900 1.33717500  C 1.98359100 0.87941400 0.93199800  C 2.47279700 0.40518200 -0.25735100  S 1.47958200 -0.86279100 -0.90094800  H -0.46688700 -1.42576600 0.51360200  H 0.25523400 0.41626200 2.25998000  H 2.45530700 1.67438200 1.49791600  H 3.35193500 0.73180500 -0.79602300  O -1.53371500 1.69452500 -0.47549600  H -0.92205900 1.00951700 -0.09561900  O -2.73939500 1.13953100 -0.52947400  O -2.68714700 -1.60760900 0.46474200  H -2.90910600 -0.73066900 0.10538100  H -3.51367100 -2.10042800 0.52579500 |
| CR-TS2-1WHC | C -0.68471600 0.15146200 0.98929500  C -0.29879800 -1.03162900 0.40190000  C -1.20773900 -1.45455800 -0.62001400  C -2.26264900 -0.59453400 -0.77945700  S -2.16553200 0.75190800 0.30960600  H -0.20893900 0.68132000 1.80417500  H 0.59613400 -1.57653200 0.68596000  H -1.07761500 -2.35624700 -1.20706000  H -3.08605700 -0.66673600 -1.47699800  O 1.96348700 0.93135600 -0.74441800  H 1.08985200 0.60164700 -0.40993400  O 2.14086800 2.13018000 -0.18642700  O 3.01950000 -1.60418900 0.34887800  H 3.19022800 -0.76699400 -0.10686300  H 3.87748800 -2.03020900 0.46242000 |
| CR-TS2-1WTA | C 0.43478000 -0.63314600 0.59888500  C 0.67660800 0.61041000 1.13653300  C 1.84252800 1.23439600 0.58996600  C 2.46774500 0.45118500 -0.34447000  S 1.64215800 -1.05654600 -0.57600300  H -0.36048900 -1.32991900 0.83782900  H 0.05343400 1.05698200 1.90374000  H 2.19494600 2.21783800 0.87849400  H 3.35875500 0.67431100 -0.91548900  O -2.02880400 0.78420300 -0.78646600  H -1.13067300 0.61263900 -0.40151800  O -2.41437100 1.96695900 -0.30493800  O -2.63061700 -1.84456700 0.41846200  H -2.96566400 -1.09177700 -0.09020500  H -3.36446900 -2.46515900 0.50123900 |
| CR-TS2-1WTD | C -0.21581900 -0.47670300 0.37035500  C -0.39298500 0.66164600 1.11856000  C 0.30838800 1.79071800 0.58457900  C 1.00681600 1.48669100 -0.55292800  S 0.81472400 -0.18177700 -0.99889500  H -0.61353500 -1.46660700 0.55245500  H -0.98673000 0.68847300 2.02587700  H 0.29207700 2.77931700 1.02838900  H 1.61657100 2.14084900 -1.16113500  O -3.33774700 -0.03939600 -0.33076000  H -2.38600100 0.17603300 -0.17510100  O -3.52512500 -1.21557500 0.26671100  O 4.06146100 -1.26313000 0.45475200  H 3.20129600 -1.03075200 0.07846900  H 4.01363000 -1.00818500 1.38435000 |
| CR3 | C -0.54860400 1.02336100 -0.58288200  C -1.61780500 1.28464300 0.22749600  C -2.23311500 0.09533600 0.74000100  C -1.61748000 -1.04812400 0.31254600  S -0.27916800 -0.69021900 -0.74345600  H 0.10452300 1.71863800 -1.09145600  H -1.95734600 2.28822200 0.45669900  H -3.09547700 0.09571500 1.39692500  H -1.87296900 -2.07511600 0.53442000  O 3.04802800 0.53483200 -0.09254800  O 2.65849100 -0.34944700 0.82424300  H 1.73782100 -0.59833000 0.56218500 |
| CR-TS3-1WHA | C 2.40603500 0.79969100 0.29611200  C 2.83063500 -0.10663200 -0.63662500  C 1.89526500 -1.17373000 -0.84101200  C 0.77961400 -1.06131500 -0.05810500  S 0.85817400 0.35153600 0.95630800  H 2.91118500 1.68739800 0.65135000  H 3.77625600 -0.02227000 -1.16026600  H 2.04920700 -1.98751300 -1.54054600  H -0.09036900 -1.70137800 -0.00634800  O -2.68376800 -1.15597200 0.43936400  O -3.53629000 -0.29341200 -0.11303800  H -2.96787800 0.48370700 -0.38760500  O -1.81700400 1.68258500 -0.75394800  H -0.97698100 1.45793600 -0.31641400  H -1.60500400 1.84384800 -1.68234000 |
| CR-TS3-1WHB | C -0.87206600 1.07814100 0.39191200  C -2.00132500 0.72446200 1.07464800  C -2.65469400 -0.42750300 0.52212900  C -2.00711800 -0.93150800 -0.57095800  S -0.59313900 0.01195400 -0.95969300  H -0.16589200 1.87656100 0.57204600  H -2.35899800 1.26138200 1.94595700  H -3.56511800 -0.85716500 0.92478800  H -2.28043600 -1.78081600 -1.18206500  O 2.97073600 -0.84900200 0.55104000  O 1.95694800 -1.68422500 0.35360200  H 1.21173800 -1.14231800 -0.02053000  O 2.13137600 1.83003200 -0.24794800  H 2.78418600 2.38352900 -0.69243800  H 2.60348300 1.03157500 0.04739500 |
| CR-TS3-1WTA | C 0.43453800 -0.63472200 0.59717800  C 0.67513500 0.60826900 1.13669900  C 1.84128500 1.23358100 0.59214400  C 2.46795800 0.45188000 -0.34258300  S 1.64339100 -1.05591400 -0.57702200  H -0.36087100 -1.33201900 0.83403600  H 0.05054700 1.05363000 1.90345500  H 2.19293700 2.21679900 0.88235300  H 3.35955500 0.67624000 -0.91220600  O -2.41148100 1.96864800 -0.30576300  O -2.02925300 0.78435300 -0.78612700  H -1.13065600 0.61164200 -0.40275500  O -2.63238800 -1.84391700 0.41869200  H -2.97004900 -1.09148900 -0.08874900  H -3.36424100 -2.46690000 0.50118500 |
| CR-TS3-1WTB | C -0.43506100 -0.96604800 1.04132300  C 0.83362000 -0.50955000 1.26507000  C 1.54152800 -0.19172200 0.05811200  C 0.79027500 -0.41198600 -1.06262300  S -0.79214700 -1.02815600 -0.66361000  H -1.18633700 -1.26517500 1.75892700  H 1.25602200 -0.39787600 2.25729800  H 2.55947100 0.18294000 0.03160900  H 1.06781800 -0.26783100 -2.09781100  O -3.72014700 0.85844500 0.52711300  O -2.95208100 1.50037900 -0.35197300  H -2.21542300 0.87093500 -0.55511300  O 4.71179900 1.04760200 -0.00133900  H 4.94445500 1.98320200 0.04962100  H 5.54960900 0.56871900 -0.02846600 |
| CR-TS4-1WHA | C 1.33815900 -1.37561100 -0.15975700  C 0.85240300 -1.01884900 1.07023600  C 1.15784500 0.33784800 1.40935900  C 1.86505200 0.98399200 0.42430700  S 2.17751400 -0.06364600 -0.92555400  H 1.24285800 -2.32592200 -0.66694200  H 0.29056500 -1.69170400 1.70730200  H 0.87763000 0.80711300 2.34602200  H 2.24890100 1.99540000 0.42830800  O -3.36794200 -0.09892300 0.46097500  H -2.66379100 0.57734100 0.24150900  O -3.46722100 -0.83994900 -0.64431600  O -1.43705400 1.59402300 -0.36355200  H -1.38604400 1.46685200 -1.32040400  H -0.55335600 1.38376700 -0.01666300 |
| CR-TS4-1WTA | C 1.11224400 -0.18287200 0.19706900  C 0.09196700 -0.97396200 0.56314700  C -1.25791700 -0.30842400 0.57352200  C -1.01456400 1.13578400 0.27518400  S 0.62728600 1.49146700 -0.13692100  H 2.15908500 -0.44824600 0.09666700  H 0.20128600 -2.02600900 0.80010600  H -1.81652900 -0.45531000 1.50859700  H -1.76969200 1.90923400 0.26047000  O -2.03547200 -1.00305000 -0.46113500  H -3.53582500 -0.07987500 -1.15647700  O -3.42218800 -0.56724900 -0.32430100  O 4.36680900 -0.80424600 -0.24718200  H 5.10471500 -0.66934500 0.36044300  H 4.75681600 -1.12071200 -1.07166500 |
| CR-TS4-1WTB | C 0.38424600 -0.75567100 0.44329100  C 0.78409200 0.21376600 1.33494500  C 1.98155700 0.88420000 0.92946600  C 2.47352400 0.40582300 -0.25707600  S 1.48272500 -0.86562800 -0.89761400  H -0.46584800 -1.42508700 0.51549600  H 0.25108000 0.42447800 2.25590700  H 2.45135200 1.68187900 1.49316600  H 3.35336400 0.73120000 -0.79535200  O -1.53627500 1.69340800 -0.47819300  H -0.92346800 1.00867100 -0.09963600  O -2.74169000 1.13764100 -0.53064300  O -2.68568900 -1.60922600 0.46516700  H -2.90937700 -0.73268600 0.10598300  H -3.51198500 -2.10170300 0.53184900 |
| IN8a-1WHA | C -0.31740300 0.87860300 -0.43000000  C -1.57069900 1.20167100 0.32683900  C -2.34862300 0.11312700 0.66543000  C -1.87687900 -1.11928300 0.20267700  S -0.41295700 -0.98954800 -0.74837400  H -0.22078800 1.36019100 -1.40863600  H -1.77379800 2.22770200 0.60924600  H -3.26395400 0.19510100 1.24298200  H -2.33701100 -2.08654400 0.35729500  O 1.97757900 1.21818800 -0.46181200  H 2.42831900 0.42786700 -0.07762300  O 0.78101900 1.25017100 0.37136300  O 2.71351900 -1.21457800 0.65605500  H 1.81084000 -1.55130500 0.52686700  H 2.86840400 -1.21520400 1.60934000 |
| IN8a-1WHB | C -0.31756600 0.87883500 -0.42987500  C -1.57057200 1.20136300 0.32753700  C -2.34810600 0.11255800 0.66602000  C -1.87623400 -1.11954600 0.20237300  S -0.41288800 -0.98879700 -0.74934000  H -0.22094700 1.36136800 -1.40804000  H -1.77366900 2.22726300 0.61042500  H -3.26315000 0.19394700 1.24409700  H -2.33615400 -2.08697900 0.35651200  O 1.97738200 1.21902200 -0.46186100  H 2.42915000 0.42981900 -0.07692600  O 0.78113700 1.25006800 0.37176600  O 2.71280700 -1.21611100 0.65651800  H 1.81005200 -1.55195100 0.52545300  H 2.86517300 -1.21579700 1.61020500 |
| IN8a-1WHC | C -0.48523700 0.81180300 -0.44662300  C -1.68751000 1.15368300 0.37837000  C -2.43816200 0.06573900 0.77688500  C -1.98823200 -1.16588700 0.29056100  S -0.58394800 -1.03655900 -0.73800300  H -0.42323100 1.30635700 -1.42196400  H -1.87946100 2.18364700 0.65357100  H -3.31600200 0.14823900 1.40942100  H -2.42753200 -2.13594600 0.48335000  O 1.82881600 1.04760900 -0.55454100  H 2.26633200 1.89914300 -0.38607500  O 0.65470500 1.18605600 0.32196900  O 3.60129800 -0.97286400 0.69410500  H 2.93320700 -0.39984800 0.28387100  H 3.10614900 -1.69508400 1.09845900 |
| IN8a-1WHC2 | C -0.04884100 0.39489000 0.10601600  C 1.01536400 1.44494500 0.18445700  C 2.30622100 0.95431600 0.19107000  C 2.40284000 -0.44057700 0.20470100  S 0.84967300 -1.24064300 0.25775500  H -0.82550500 0.44077300 0.87562600  H 0.74186200 2.49293100 0.17544400  H 3.18509300 1.59094500 0.19801200  H 3.31238700 -1.02677300 0.21333100  O -1.82471900 -0.32380600 -1.26340500  H -1.45216700 -1.08835200 -1.73614400  O -0.66228100 0.55734800 -1.18137700  O -3.20829400 0.11402700 1.25110100  H -4.02822700 0.62151300 1.26189600  H -3.01937300 -0.08275500 0.31973100 |
| IN8a-1WTA2a | C 0.07404100 0.24417100 -0.08139400  C -0.75485600 1.44254100 0.26308700  C -2.11792400 1.22504600 0.23295200  C -2.49854500 -0.05760300 -0.17498400  S -1.14592200 -1.08513600 -0.58451500  H 0.79316200 0.39072000 -0.89333100  H -0.26904400 2.36864200 0.54453400  H -2.84753100 1.98555300 0.49210300  H -3.50916100 -0.43411700 -0.26464700  O 1.78809300 -1.11392700 0.73154300  H 1.54595300 -1.82446500 1.34754700  O 0.79721400 -0.09585200 1.10277800  O 3.30272200 0.83379300 -0.81752900  H 3.16502700 0.11463700 -0.18189800  H 4.13582000 0.64416200 -1.26437800 |
| IN8a-1WTA2b | C 1.36091100 0.35044200 -0.41322100  C 0.73743000 1.68205500 -0.12922800  C -0.62425500 1.64127900 0.10012100  C -1.21251200 0.37979100 -0.03467000  S -0.06928500 -0.85947000 -0.50154800  H 1.92214200 0.28044300 -1.35109900  H 1.35966400 2.56641600 -0.06354000  H -1.20938800 2.51804800 0.35922900  H -2.25750300 0.12547900 0.09996100  O 3.03187300 -1.11057400 0.29590900  H 2.65154100 -1.76500100 0.90639900  O 2.25391400 0.07170000 0.66158500  O -4.42461600 -0.53397200 0.32318500  H -4.79216200 -0.97932700 1.09681100  H -5.02455100 -0.73317500 -0.40644400 |
| IN8a-1WTB2 | C 1.05668700 -0.39194900 0.54802400  C -0.36276100 -0.86957300 0.56960700  C -1.29364000 0.05069300 0.12934900  C -0.75990100 1.30126000 -0.20106600  S 0.96690500 1.41613700 0.05510800  H 1.59263300 -0.45464600 1.50109100  H -0.58197800 -1.88881700 0.86438200  H -2.35578300 -0.16105100 0.04849400  H -1.30784300 2.15951000 -0.56764500  O 3.17565400 -0.88974000 -0.28277900  H 3.32463600 -0.40581700 -1.11300100  O 1.74999500 -1.18892800 -0.40686100  O -4.63025500 -0.54168100 -0.22723800  H -5.01387500 -0.99663800 -0.98744100  H -5.33373300 -0.49053000 0.43190800 |
| TS1 | C 1.15871400 1.25846400 -0.12305800  C -0.04309200 1.15839300 0.49152800  C -0.44403400 -0.22237100 0.76706700  C 0.63169100 -1.10286800 0.46175100  S 1.93394900 -0.30576800 -0.33527900  H 1.65679500 2.15105500 -0.47804400  H -0.67416200 2.00945500 0.71957900  H -1.11728500 -0.45325700 1.58614400  H 0.64378000 -2.17531300 0.59686900  O -1.69588100 -0.63324200 -0.49721000  O -2.86500300 0.12636900 -0.22661800  H -2.78490500 0.86561900 -0.85318500 |
| TS1-1WHA | C 1.04835500 -0.93742800 0.60553100  C 0.01064600 0.03627800 0.69635100  C 0.51558800 1.34048900 0.26540800  C 1.75583800 1.28005500 -0.27233200  S 2.44650300 -0.33760800 -0.20191400  H 0.96834800 -1.98196500 0.87158200  H -0.73239000 -0.04302100 1.48376100  H -0.07278100 2.24853100 0.32422800  H 2.33373000 2.08489100 -0.70744500  O -1.92442700 -1.50237200 -0.22533600  H -2.72609700 -1.06826600 0.13540700  O -1.14937900 -0.38486100 -0.64057300  O -3.44174100 0.86719400 0.13975800  H -4.14955500 1.16565100 -0.44334800  H -2.62349100 0.83984500 -0.39409800 |
| TS1-1WHB | C 0.54364700 -0.39530000 -1.15438700  C 0.05394500 -1.26132200 -0.13341000  C 0.88298200 -1.13872500 1.06443800  C 1.79291800 -0.14058100 0.97776200  S 1.77714300 0.65850100 -0.58952600  H 0.13922300 -0.28903700 -2.15118300  H -0.40517000 -2.20586200 -0.40758200  H 0.73052200 -1.74211400 1.95095400  H 2.48484400 0.19788600 1.73768300  O -2.53586200 -0.76830200 -0.52702100  H -2.98372200 -1.55260000 -0.16721200  O -1.52116000 -0.52524900 0.44694300  O -1.46770900 2.37264800 0.48375200  H -2.36020100 2.64121600 0.23669500  H -1.48287900 1.39727700 0.47726300 |
| TS1-1WTA | C -0.22493000 0.77752200 0.39621600  C -0.14214300 -0.60699600 0.72496100  C -1.43958800 -1.25113600 0.52316700  C -2.34487000 -0.44256400 -0.07487700  S -1.72442100 1.18304500 -0.34515900  H 0.57895400 1.49964800 0.47373300  H 0.52668500 -0.92508500 1.51921400  H -1.62632600 -2.28986500 0.76745300  H -3.35394900 -0.68458100 -0.38185600  O 2.22397800 -1.16099300 -0.41521100  H 2.50670600 -1.92986600 0.10749100  O 0.82814800 -1.39153600 -0.61388800  O 2.89672400 1.54260500 0.28018400  H 3.49597600 1.98402400 -0.33279400  H 2.78108100 0.63544100 -0.05618000 |
| TS1-1WTA2 | C 0.96796000 1.21477500 0.29342800  C 1.20014700 -0.09146400 0.80698500  C -0.06498700 -0.81544200 0.91897000  C -1.11091900 -0.16116300 0.36161700  S -0.65375200 1.42651900 -0.24886400  H 1.71528800 1.97577800 0.11837800  H 2.00733300 -0.25481000 1.51432500  H -0.13746300 -1.80980000 1.34319900  H -2.13878100 -0.48747600 0.25445000  O 3.37305600 -0.58440200 -0.64421300  H 3.84409100 -1.25043200 -0.11502100  O 2.01992000 -1.01154500 -0.54863700  O -4.28650800 -0.89807700 -0.34221500  H -5.12720000 -0.65970900 0.06794800  H -4.50818400 -1.26589200 -1.20693200 |
| TS1-1WTB | C 1.03728800 0.19609500 -1.17690100  C -0.10251600 -0.24689800 -0.44698400  C 0.32638800 -0.85374000 0.81447000  C 1.65013400 -0.71367900 1.05740600  S 2.49201200 0.08875700 -0.26377300  H 1.03627700 0.64830200 -2.15885800  H -0.97974200 -0.61907800 -0.96776000  H -0.36320500 -1.34207600 1.49314600  H 2.20959700 -1.03202000 1.92725200  O -2.08148700 1.10752600 0.75669300  H -1.77907400 1.13457400 1.67986700  O -0.88493500 1.34921500 0.01551400  O -3.31265300 -1.11446500 -0.53967100  H -3.10406400 -0.28642300 -0.07191400  H -4.12713400 -0.95225700 -1.02960900 |
| TS2 | C -0.24523400 -0.11597100 0.71392100  C 0.10681200 1.24404800 0.52830700  C 1.29354100 1.41613900 -0.20677100  C 1.92047700 0.21950300 -0.50390600  S 1.05615600 -1.15161000 0.12536600  H -0.85786400 -0.48361400 1.52610300  H -0.51969000 2.05363100 0.88412000  H 1.68486200 2.37939900 -0.51399000  H 2.82830400 0.07209900 -1.07358400  O -2.82271000 0.10386700 -0.11075100  H -2.88363400 0.90841300 -0.65395200  O -1.62779700 -0.48967800 -0.55973100 |
| TS2-1WHA | C 0.39691800 -0.64602700 0.68658600  C 0.31435200 0.71121800 1.09552800  C 1.21093500 1.55280900 0.41424100  C 2.05718700 0.86411100 -0.43694900  S 1.76299900 -0.85144500 -0.41399100  H 0.11852200 -1.48724900 1.30750100  H -0.42254700 1.05035500 1.81360400  H 1.24096800 2.62984900 0.53341700  H 2.81892900 1.27393500 -1.08686400  O -2.21064600 -1.24272600 0.38997100  H -2.71628500 -0.40057900 0.36714000  O -1.11098100 -0.94564600 -0.43890700  O -2.67260500 1.34592300 -0.53670600  H -3.26601400 1.53068400 -1.27456900  H -1.90404400 0.87305400 -0.90769000 |
| TS2-1WHB | C 0.07520400 0.08292800 0.64813500  C 1.04903300 1.10912800 0.77436000  C 2.25130500 0.81620300 0.11257700  C 2.26300900 -0.45510800 -0.44015200  S 0.78995200 -1.32387500 -0.14113400  H -0.73297400 -0.09472500 1.34581900  H 0.84063400 2.03660400 1.29465000  H 3.08850100 1.50088500 0.03720100  H 3.05660400 -0.91500600 -1.01434800  O -1.81759400 1.84369600 -0.27245500  H -1.32345000 2.55832800 -0.70880300  O -1.13754400 0.68533000 -0.71919700  O -2.71282500 -1.41602300 0.33392700  H -2.39048600 -0.66546400 -0.20063200  H -3.66566700 -1.46156400 0.19653300 |
| TS2-1WHC | C 0.51049200 0.07137400 0.76484200  C 1.20249800 1.27143300 0.47387200  C 2.33968800 1.07471300 -0.32932000  C 2.59284700 -0.26189500 -0.58228200  S 1.43145000 -1.30904500 0.17411300  H -0.13914200 -0.07373800 1.61721000  H 0.84467500 2.23767700 0.80960700  H 2.95920100 1.87618000 -0.71515200  H 3.38719000 -0.68128600 -1.18533900  O -1.96007500 0.98005800 0.00074200  H -1.84180800 1.72530400 -0.61290200  O -1.00766700 0.03589300 -0.44523700  O -4.53063900 -0.48206900 -0.07709400  H -3.68358300 -0.01144800 -0.01151400  H -4.31583800 -1.33278200 -0.47766900 |
| TS2-1WTA | C -0.03871100 -0.26309000 -0.35293200  C -0.50306600 0.96089200 -0.89797700  C -1.86688700 1.19780000 -0.65557700  C -2.49031500 0.13777100 -0.01951800  S -1.40125900 -1.18105200 0.28622200  H 0.82557700 -0.80402800 -0.72058100  H 0.15973200 1.64406400 -1.41638700  H -2.38740500 2.10666300 -0.93553000  H -3.52344800 0.07191300 0.29508900  O 2.06835600 0.85226600 1.06325000  H 1.84120100 1.79638000 1.10498400  O 0.83498000 0.20163900 1.30945000  O 3.10353100 -0.64817200 -1.13004400  H 2.99669000 -0.12186800 -0.31834300  H 3.84671900 -1.24239500 -0.97400900 |
| TS2-1WTD | C -0.43478900 -0.45230200 0.24127900  C -0.39040100 0.58365000 1.20499400  C 0.29420300 1.73164700 0.76016900  C 0.87133300 1.56719400 -0.48407500  S 0.57924500 -0.02124900 -1.14027600  H -0.55435600 -1.50222100 0.47321100  H -0.88759300 0.49775800 2.16416700  H 0.36549200 2.65391500 1.32566300  H 1.42718200 2.29544300 -1.05945600  O -3.09920000 -0.94730200 0.34941600  H -3.53455900 -0.17305400 0.74642200  O -2.18069400 -0.37211200 -0.54394300  O 3.48585600 -1.43414100 0.56156200  H 2.78388200 -1.11147200 -0.02173700  H 3.44226300 -0.87308300 1.34565800 |
| TS3 | C -0.65886000 0.96447600 -0.83316700  C -1.67856500 1.05204100 0.05984800  C -1.83174500 -0.13762200 0.87325300  C -0.92638700 -1.11127300 0.57698800  S 0.11480100 -0.61040500 -0.74171600  H -0.29921800 1.68872100 -1.54954800  H -2.32034200 1.92100800 0.14871700  H -2.60675000 -0.25062500 1.62295500  H -0.81985100 -2.10199700 0.99547200  O 1.75272700 -0.33841900 -0.25254900  O 2.29151400 0.80615000 0.87715900  H 2.42876100 0.16179300 1.59145500 |
| TS3-1WHA | C 1.75121800 -0.86680100 -0.37081800  C 2.39395800 -0.07272800 0.52313200  C 1.70254500 1.17782500 0.78554600  C 0.55114600 1.31527600 0.07931300  S 0.30307100 -0.07426200 -0.96817400  H 2.02084000 -1.84127000 -0.75145800  H 3.33624700 -0.34046200 0.98749100  H 2.07550300 1.93088000 1.47044000  H -0.18389500 2.10834500 0.07563900  O -1.09808700 -0.97610300 -0.38662700  O -1.42227800 -1.59226800 1.17042200  H -1.77393600 -0.76304200 1.54214800  O -2.79387100 1.21608400 0.02975900  H -3.73935600 1.23691200 -0.15810200  H -2.46384900 0.35369500 -0.28684200 |
| TS3-1WHB | C -0.46472800 1.25451900 0.04067100  C -1.61482800 1.24753100 0.75823900  C -2.41360500 0.05080000 0.55800400  C -1.85102900 -0.83432300 -0.30773200  S -0.34962400 -0.19629100 -0.95022300  H 0.35627700 1.95843900 0.00665200  H -1.91012700 2.05557000 1.41769900  H -3.37336700 -0.11102400 1.03594600  H -2.22667100 -1.78374000 -0.66186500  O 1.06986000 -1.08698300 -0.34316500  O 1.63136800 -1.31610200 1.24375400  H 1.32992300 -2.23704100 1.30858500  O 2.74769700 1.29876500 -0.21525400  H 3.41967500 1.41433700 0.46662000  H 2.47201600 0.36751500 -0.14782600 |
| TS3-1WTA | C -0.30081600 1.05919500 -0.47020300  C -1.27133300 1.57295400 0.32359600  C -2.27717500 0.60092500 0.71582600  C -2.04722200 -0.64133400 0.21172000  S -0.62276200 -0.64049200 -0.81480900  H 0.61106600 1.50654000 -0.84720600  H -1.28702500 2.60900800 0.64203900  H -3.13435500 0.84641100 1.33321100  H -2.63402700 -1.54336600 0.31330700  O 0.79454900 -1.50813200 -0.14101500  O 1.96594100 -1.04773500 0.98231600  H 1.47001800 -1.25800900 1.78975300  O 2.75378200 1.39150200 -0.17561600  H 2.55151100 0.56170000 0.30497300  H 3.65210900 1.29006800 -0.51024400 |
| TS3-1WTB | C -0.63556100 -1.28802400 0.84509300  C 0.68099900 -1.01534100 1.03768900  C 1.27797300 -0.24730400 -0.03882300  C 0.39434700 0.04330100 -1.03359100  S -1.18061800 -0.65212100 -0.69906500  H -1.33531200 -1.81719900 1.47552700  H 1.23684900 -1.34736900 1.90718000  H 2.32368600 0.04281100 -0.05805900  H 0.55639700 0.57216900 -1.96217300  O -2.39196600 0.55305900 -0.35328800  O -2.38443900 1.68563900 0.90697700  H -2.08339500 2.42615000 0.35484000  O 4.50086300 0.72797700 0.01327600  H 4.79468500 1.55392800 0.41788500  H 5.29478000 0.31425300 -0.34808700 |
| TS4 | C -1.19050200 1.26018400 -0.09298200  C 0.08950800 1.18572100 0.29818100  C 0.57794800 -0.21023900 0.53470200  C -0.57783800 -1.11931400 0.34873000  S -2.00141700 -0.31713700 -0.19817500  H -1.75105700 2.14977600 -0.35112600  H 0.75760400 2.03208100 0.39684000  H 1.12006600 -0.34439500 1.47770600  H -0.56307100 -2.19142300 0.48814600  O 1.61000200 -0.51671200 -0.52937700  H 2.45125600 -1.02261900 -0.15866100  O 2.96664500 0.23579400 -0.12236000 |
| TS4-1WHA | C -2.13112700 0.95526700 0.00610400  C -0.88200600 1.30342600 0.34407000  C 0.07117400 0.15063300 0.46759500  C -0.71767100 -1.09057800 0.25130800  S -2.36265600 -0.79733400 -0.18079900  H -2.97491600 1.61074200 -0.16955300  H -0.53931200 2.32219000 0.47592900  H 0.64884200 0.15247800 1.40023900  H -0.32749200 -2.09702900 0.30907500  O 1.09370900 0.26019900 -0.61290100  H 2.14008900 0.06589300 -0.25600600  O 2.10764500 1.38847200 -0.19698500  O 3.42054700 -1.06193200 0.17492200  H 4.05236000 -0.35701600 0.37586300  H 3.78549500 -1.54632100 -0.57751500 |
| TS4-1WTA | C 1.12593600 -0.19672800 0.19158300  C 0.11428500 -0.99580100 0.56958400  C -1.22784800 -0.32997200 0.59986300  C -1.01218200 1.10241000 0.28599200  S 0.62143200 1.47349100 -0.12854500  H 2.17388600 -0.45281300 0.07682200  H 0.23194400 -2.04625700 0.80885400  H -1.82370600 -0.50132400 1.50277200  H -1.78501300 1.85754400 0.27759100  O -2.07956300 -1.03825400 -0.44017300  H -2.47233000 -0.39340600 -1.18286800  O -3.54728100 -0.42761200 -0.37614600  O 4.36142100 -0.75926500 -0.25964700  H 5.07982500 -0.65549300 0.37694800  H 4.77471900 -1.06252200 -1.07781600 |
| TS4-1WTB | C -1.23834900 1.40978800 -0.13714700  C -0.04032300 0.94267200 0.24225100  C -0.02814400 -0.52815600 0.52451000  C -1.41430300 -1.02415700 0.38201300  S -2.51602200 0.17262200 -0.17306600  H -1.49162200 2.42322900 -0.42145200  H 0.87045600 1.53113400 0.29847000  H 0.47821600 -0.81789500 1.45155300  H -1.74116900 -2.03762500 0.57042700  O 0.81315400 -1.20346900 -0.56300800  H 1.32248400 -2.08223300 -0.22709300  O 2.35235900 -1.28481300 -0.09619000  O 3.12822500 1.40326100 0.10584900  H 2.98329200 0.44214700 -0.02157000  H 3.81151500 1.65856000 -0.52423800 |
| TS5 | C 0.50782500 -1.05519100 0.48166900  C -0.58225500 -0.08488600 0.75180000  C -0.05087900 1.28413200 0.40033700  C 1.16205600 1.27317000 -0.16185100  S 1.86182600 -0.37040700 -0.31297400  H 0.46741600 -2.10544100 0.73181400  H -1.10559700 -0.14788100 1.71382400  H -0.64312800 2.17912600 0.54854900  H 1.72746600 2.10662900 -0.55955700  O -1.27092400 -0.72058100 -0.29279300  O -2.89257500 0.17689000 -0.34296700  H -3.14786600 -0.25973200 -1.17270100 |
| TS5-1WHA | C 0.99104800 -0.24921500 -1.16413900  C 0.13307300 -1.13913100 -0.34347000  C 0.58475100 -0.98852100 1.08937000  C 1.51371500 -0.04444300 1.27408900  S 2.00855000 0.77445500 -0.24354400  H 0.96931100 -0.20803100 -2.24379600  H -0.00303700 -2.17401800 -0.67888800  H 0.14594300 -1.57511500 1.88760000  H 1.95619600 0.29320900 2.20305700  O -0.94784700 -0.31502900 -0.70422500  O -2.41809900 -1.17575200 0.06961900  H -2.92632000 -0.34247400 0.13123700  O -2.56772400 1.81681900 0.19355600  H -1.75598300 1.35188200 -0.07702800  H -2.38906100 2.18282900 1.06782400 |
| TS5-1WHA2 | C 1.00679100 -0.53483300 -1.07871700  C 0.06247800 -1.20039000 -0.14771500  C 0.43360600 -0.75683800 1.24643000  C 1.38670700 0.17960000 1.28829200  S 2.00773400 0.63330300 -0.33249900  H 1.04998700 -0.72596500 -2.14153800  H -0.09728900 -2.27845500 -0.26374200  H -0.08213300 -1.13878100 2.11917200  H 1.77730800 0.70647200 2.14972200  O -0.96638000 -0.44269000 -0.74377400  O -2.51317600 -1.04710700 0.12530700  H -2.81460900 -0.13804500 0.32472300  O -2.17074900 1.93354000 0.13627400  H -2.55714000 2.44681300 -0.58323400  H -1.53491200 1.31992700 -0.27731000 |
| TS5-1WHB | C 0.36679100 -0.97982500 0.21854200  C -0.16670600 0.33967100 0.64118500  C 0.98449300 1.31402400 0.60922700  C 2.11748800 0.80452300 0.11446100  S 1.97382500 -0.92190100 -0.34978500  H -0.21906200 -1.89004400 0.22441400  H -0.77552500 0.36465100 1.55263000  H 0.86809000 2.34401400 0.92497800  H 3.06054800 1.30558900 -0.06474300  O -0.95230000 0.34414800 -0.53279200  O -2.06769500 1.80803700 -0.36770300  H -1.94272700 2.05511100 -1.29957800  O -2.76560300 -1.65150900 0.21521600  H -3.69419000 -1.47765300 0.40604400  H -2.40594200 -0.82701400 -0.16544600 |
| TS5-1WTA | C 0.97128800 1.41363100 0.19497200  C 1.36710900 0.07219500 0.69243900  C 0.10242200 -0.74464900 0.80336800  C -0.99293500 -0.14140600 0.32914500  S -0.66851000 1.51559700 -0.28100400  H 1.64445700 2.25394300 0.10552300  H 2.03130500 0.02082300 1.56449600  H 0.11387200 -1.75747700 1.18841500  H -2.00235200 -0.52602700 0.23208700  O 2.04990400 -0.07804600 -0.52498600  O 2.85854200 -1.74194700 -0.36206300  H 3.17845000 -1.70869100 -1.27875400  O -4.12340000 -1.09825300 -0.21764200  H -4.89117800 -1.11722100 0.36731600  H -4.34605100 -1.66756200 -0.96505100 |
| TS5-1WTB | C -0.77875700 -1.11176900 0.11260400  C 0.20706300 -0.11324100 -0.37148700  C -0.57736000 1.09704800 -0.81714800  C -1.88828100 1.02628700 -0.56459200  S -2.37495400 -0.51624300 0.21068200  H -0.52375300 -2.11705500 0.41631400  H 0.97665700 -0.46547000 -1.06882000  H -0.09155600 1.95920600 -1.25861900  H -2.65080000 1.77702200 -0.73004100  O 0.67945500 -0.02894700 0.95662400  O 2.15295800 1.06167700 0.88677000  H 2.24729100 1.04539600 1.85297600  O 3.30159600 -0.85540700 -0.77776200  H 3.05815200 -0.18609500 -0.10213800  H 4.13521400 -0.56165600 -1.16190700 |
| TS5-1WTB2 | C 1.37074300 -0.91204300 0.57028200  C -0.01921000 -0.40624800 0.71050200  C -0.01738600 1.02944900 0.23798100  C 1.14793800 1.42964100 -0.28027500  S 2.41588800 0.15912600 -0.24263500  H 1.69911500 -1.88684800 0.90158000  H -0.52222200 -0.57385500 1.67130800  H -0.91661400 1.63585700 0.28310000  H 1.39275400 2.37910100 -0.73937100  O -0.39262200 -1.34885600 -0.26961500  O -2.22777700 -1.30546300 -0.38910800  H -2.19447800 -1.59578700 -1.31503600  O -3.13945200 1.28411400 0.07579400  H -4.03066100 1.25863300 0.44172800  H -2.89580400 0.35372500 -0.12865200 |
| TS8a | C -0.45700400 0.45865500 0.47856600  C 0.67191000 1.41739400 0.29632700  C 1.86273700 0.90778100 -0.16657300  C 1.88197600 -0.49705000 -0.26302200  S 0.44827300 -1.31115400 0.10868900  H -0.88292700 0.30927200 1.48017900  H 0.47065800 2.47241900 0.44364300  H 2.72283400 1.51625800 -0.42325100  H 2.75859200 -1.05881900 -0.57240400  O -2.78508000 -0.29309000 -0.01445200  H -3.24189400 -0.06174900 -0.83973400  O -1.30958800 0.80314000 -0.47295300 |
| TS8a-1WHA | C 0.24672800 -0.15530000 0.98479700  C 1.18535900 1.00398200 1.02902000  C 2.06849300 1.11984000 -0.02190300  C 2.03987100 0.02114900 -0.89875500  S 0.88989500 -1.18003000 -0.57054900  H 0.25535200 -0.87806000 1.81119000  H 1.06392200 1.73446000 1.82049400  H 2.73915000 1.95964100 -0.16485900  H 2.69425500 -0.07980600 -1.75922100  O -2.09774500 -1.07017000 0.87441400  H -2.66285600 -0.72038300 0.15750400  O -0.93432500 0.43745300 0.76522500  O -2.74941100 0.91415000 -1.31936300  H -1.97835100 1.02452500 -0.73426900  H -3.34064700 1.65060800 -1.12322000 |
| TS8a-1WHB | C -0.10921400 -1.09377400 -0.17134200  C -1.12464800 -0.77469600 -1.21764500  C -1.98363800 0.26357500 -0.92460000  C -1.86567000 0.72549500 0.39525300  S -0.65722800 -0.00163700 1.34074600  H -0.03764000 -2.11918500 0.21141400  H -1.07064800 -1.28756300 -2.17052700  H -2.69717300 0.68134700 -1.62541600  H -2.48280100 1.50908700 0.82274200  O 2.33419100 -0.98708700 0.49659300  H 2.95658200 -1.28237300 -0.18886300  O 1.00973200 -0.63289100 -0.77398900  O 1.71660800 2.17140100 -0.53537800  H 2.35524800 2.15884300 0.18796600  H 1.50684400 1.23104500 -0.67704300 |
| TS8a-1WHC | C -0.35862400 0.92805700 -0.43087000  C -1.54377100 1.20176700 0.43390100  C -2.29547400 0.10663000 0.79995300  C -1.90092100 -1.08034800 0.15929200  S -0.54752100 -0.97479200 -0.85608200  H -0.28197300 1.43098100 -1.40357800  H -1.69633400 2.21025600 0.79962700  H -3.12676200 0.14340600 1.49488700  H -2.40119400 -2.03474700 0.29014800  O 2.14631100 0.99130200 -0.66284100  H 2.58146200 1.76472700 -0.26751700  O 0.65028200 1.17868200 0.42390000  O 2.85883300 -1.31653000 0.86913400  H 2.66715100 -0.55106600 0.29061200  H 2.36732700 -1.13115500 1.67792900 |
| TS8a-1WHC2 | C 0.05168700 0.54867700 0.47519300  C 1.41634200 1.10702000 0.70843600  C 2.46879400 0.38500900 0.18929100  C 2.08119700 -0.85063000 -0.35557400  S 0.42251300 -1.20713400 -0.31822900  H -0.60102700 0.33469400 1.32924900  H 1.50002700 2.08374000 1.16981300  H 3.50015300 0.71901800 0.20340200  H 2.77587900 -1.56548300 -0.78567000  O -2.24756000 1.21653200 -0.49437000  H -2.24700300 1.38124200 -1.45068600  O -0.42467200 1.45774500 -0.40413700  O -2.54927300 -1.28687200 0.57739600  H -3.41863000 -1.47116800 0.94974300  H -2.60568900 -0.40759000 0.14062500 |
| TS8a-1WTA | C 0.10060200 0.32620500 -0.08030300  C -0.86127400 1.44674000 0.12478200  C -2.19881200 1.11177000 0.13694500  C -2.44224000 -0.22943300 -0.19878200  S -1.07547300 -1.19871200 -0.47317000  H 0.80746400 0.38230300 -0.91783500  H -0.46258400 2.43225200 0.33370600  H -2.99821100 1.80972000 0.35896300  H -3.43454400 -0.66239300 -0.27814200  O 2.04878500 -0.95392100 0.91678600  H 2.26802800 -0.91330900 1.86118500  O 0.65811900 0.25387600 1.14289500  O 3.12540000 0.68007400 -1.04421500  H 2.93468800 0.09762100 -0.27648600  H 3.84462800 0.26127100 -1.53025700 |
| TS8a-1WTA2 | C 1.37971700 0.39724700 -0.40674400  C 0.64684300 1.67947100 -0.19265700  C -0.70340200 1.59126800 0.05795400  C -1.22526100 0.28974800 -0.07016600  S -0.10596800 -0.93433600 -0.41516500  H 1.88485600 0.22670900 -1.36785800  H 1.22403600 2.59613500 -0.15191100  H -1.32935200 2.43825900 0.31644600  H -2.27805200 0.04349500 0.05074500  O 3.21271000 -1.18120700 0.21136400  H 3.57284800 -1.23024000 1.11166800  O 2.15212100 0.30783000 0.67283300  O -4.39497600 -0.51964300 0.31916400  H -4.72617900 -0.95271700 1.11611200  H -4.99888800 -0.77450900 -0.38977700 |
| TS8a-1WTB | C 1.08689500 -0.44333300 0.54182000  C -0.32750300 -0.91512700 0.60484900  C -1.30537300 -0.04141300 0.18553900  C -0.81379000 1.24353600 -0.11129400  S 0.86045600 1.47356200 0.00319000  H 1.67094700 -0.37671600 1.47043500  H -0.50604600 -1.94664100 0.88686700  H -2.35866000 -0.28993600 0.10170200  H -1.45267800 2.06751800 -0.41511700  O 3.43214300 -0.70220500 -0.26860600  H 3.64508100 -1.15066400 -1.10302900  O 1.61727600 -1.18964200 -0.42058300  O -4.59138700 -0.54331900 -0.26275500  H -4.94870500 -0.93670300 -1.06896900  H -5.32286700 -0.52450800 0.36715400 |
| CP-TS1-WHA | C 1.06468500 -1.02786400 0.37090500  C -0.03386400 -0.03496100 0.57312800  C 0.57335100 1.32129100 0.33477200  C 1.84007200 1.29603800 -0.10930000  S 2.52043100 -0.33195800 -0.25583600  H 0.94113300 -2.10032600 0.42446500  H -0.51600900 -0.13798100 1.55501300  H 0.00869800 2.23317600 0.49324800  H 2.46241800 2.14651800 -0.35938600  O -1.86031900 -1.38204000 -0.06111900  H -2.73738100 -0.97775100 0.11203700  O -1.11212300 -0.17759200 -0.41659600  O -3.75097700 0.68540900 -0.02696900  H -4.44490800 0.83147000 -0.68126000  H -2.91895700 0.98297600 -0.43029800 |
| CP-TS1-WHB | C 0.63187200 -0.51765800 -1.05974600  C -0.00281000 -1.11468900 0.15082100  C 0.82110700 -0.67331600 1.32850800  C 1.79191800 0.20396000 1.02755800  S 1.91068200 0.58631500 -0.69631200  H 0.27285000 -0.63934100 -2.07190500  H -0.12682000 -2.20327600 0.07118000  H 0.60557800 -1.01711000 2.33334000  H 2.48508100 0.67279200 1.71493500  O -2.24140900 -1.11881700 -0.62720100  H -2.87140000 -1.61751000 -0.08092500  O -1.36064600 -0.58919900 0.41314900  O -2.14505600 2.25557300 0.30033300  H -2.58776100 2.31328400 -0.55461800  H -1.82407700 1.33988400 0.35589400 |
| CP-TS1-1WHA2 | C 1.20414700 -0.96882700 -0.66147800  C 0.07775600 -0.95160200 0.31726900  C 0.33857600 0.21094100 1.24294500  C 1.41269600 0.94958800 0.92848700  S 2.30131400 0.35594500 -0.49090200  H 1.31742000 -1.68553900 -1.46260800  H -0.03357000 -1.90487900 0.85466700  H -0.31671300 0.42146500 2.08017800  H 1.77831800 1.83440600 1.43534400  O -1.15372400 -0.79384000 -0.45517300  O -2.26621000 -1.04345100 0.46844400  H -2.76108300 -0.20507900 0.34508100  O -2.86440000 1.45202800 -0.64019500  H -2.02971200 1.13853000 -1.02498500  H -2.70005500 2.34748500 -0.32119000 |
| CP-TS1-WHB2 | C 0.38771600 -0.98115000 0.29760700  C -0.16160100 0.38205900 0.53574000  C 0.98090200 1.34618700 0.36308100  C 2.12864300 0.78065000 -0.04076500  S 2.03176300 -0.98037200 -0.22940500  H -0.19067600 -1.89347700 0.35450000  H -0.67911200 0.47746500 1.49991100  H 0.86306600 2.40872200 0.54246200  H 3.06872800 1.27565700 -0.25156000  O -1.21491400 0.57977000 -0.48736300  O -1.96981100 1.78479400 -0.12026400  H -1.76257800 2.35505100 -0.87878500  O -2.76951300 -1.79580600 0.05512700  H -3.72795200 -1.73064800 0.13541500  H -2.45973300 -0.92336700 -0.24544200 |
| CP-TS1-1WTA2a | C -0.19298800 0.66425200 0.52710100  C -0.14144200 -0.82882000 0.51467500  C -1.50883800 -1.30387600 0.10755900  C -2.35304800 -0.32296000 -0.25140600  S -1.67116100 1.30312800 -0.10738400  H 0.65475700 1.31175600 0.71369200  H 0.21311000 -1.24942500 1.46558500  H -1.75952200 -2.35802700 0.08457700  H -3.37534000 -0.43398900 -0.59161700  O 2.13661400 -1.19080900 0.00002900  H 2.47915500 -2.09265600 -0.10347400  O 0.77445500 -1.36275500 -0.51432000  O 3.03478500 1.57174300 -0.01096000  H 3.26773700 1.94802200 -0.86773500  H 2.86975300 0.62727300 -0.16844700 |
| CP-TS1-1WTA2b | C 0.99444100 1.15350500 0.27474500  C 1.26538500 -0.28823400 0.56098500  C -0.07269000 -0.97678500 0.56822600  C -1.10909800 -0.20248400 0.20944400  S -0.65486600 1.47587100 -0.13845800  H 1.75580900 1.91172400 0.15682800  H 1.83959400 -0.42856500 1.48855000  H -0.16584000 -2.02806300 0.81580000  H -2.15317500 -0.48412500 0.12564300  O 3.40889600 -0.43529400 -0.40444200  H 3.88887900 -1.25891400 -0.22024800  O 2.04198400 -0.94950700 -0.49531200  O -4.36438300 -0.84142000 -0.23203600  H -5.11188900 -0.58233500 0.32113600  H -4.73571500 -1.04990400 -1.09847000 |
| CP-TS1-1WTB1 | C 1.33669500 -0.84356400 0.73251600  C -0.06500300 -0.40241400 0.48453000  C 0.01972500 0.93080000 -0.21401600  C 1.26574500 1.31209600 -0.52917800  S 2.52325700 0.17548200 0.00323600  H 1.61703100 -1.76907700 1.21536800  H -0.67559100 -0.37206000 1.39842300  H -0.86770600 1.51719900 -0.42446100  H 1.56883500 2.21429600 -1.04612700  O -2.08881000 -1.19095700 -0.50826100  H -2.13535400 -1.03011000 -1.46513200  O -0.64832400 -1.45818200 -0.35965800  O -3.28084400 1.27826200 0.39344600  H -3.00416800 0.35281200 0.28176300  H -4.07430700 1.26473100 0.94106700 |
| CP-TS1-1WTB2 | C -0.77494300 -1.10294300 0.23457900  C 0.16183700 -0.03565300 -0.21689800  C -0.69270000 1.10176000 -0.71404700  C -2.01155600 0.92071700 -0.55531700  S -2.43823500 -0.64363900 0.16921100  H -0.47294300 -2.06484100 0.62367400  H 0.89122800 -0.39800400 -0.95520200  H -0.25281300 1.99632200 -1.13919000  H -2.80876900 1.60771900 -0.81148700  O 0.94364100 0.33058200 0.96996800  O 2.05511300 1.18218200 0.48848700  H 2.08117400 1.83190800 1.20854500  O 3.49264200 -1.10996100 -0.58057400  H 3.24031600 -0.30640400 -0.09646900  H 4.30656100 -0.89419100 -1.05020700 |
| CP-TS1-1WTB2a | C 1.33676800 -0.84335700 0.73276300  C -0.06502000 -0.40250300 0.48454600  C 0.01958700 0.93064700 -0.21423200  C 1.26556100 1.31206200 -0.52937700  S 2.52321800 0.17569900 0.00328700  H 1.61719200 -1.76886100 1.21558600  H -0.67568600 -0.37202300 1.39837700  H -0.86792700 1.51687500 -0.42480900  H 1.56857900 2.21419900 -1.04648200  O -0.64801900 -1.45838600 -0.35951200  O -2.08854100 -1.19114900 -0.50842200  H -2.13485000 -1.03072800 -1.46537000  O -3.28094900 1.27834000 0.39368800  H -4.07529400 1.26505200 0.94003500  H -3.00481100 0.35276700 0.28183000 |
| CP-TS1-1WTB2b | C -0.77128400 -1.10283400 0.23139300  C 0.16237600 -0.03281800 -0.21994800  C -0.69543700 1.10410100 -0.71251300  C -2.01356900 0.92029300 -0.55092900  S -2.43559200 -0.64649700 0.17107700  H -0.46662700 -2.06547900 0.61654000  H 0.89087200 -0.39224300 -0.96053000  H -0.25823200 2.00047400 -1.13661500  H -2.81265700 1.60649400 -0.80337300  O 0.94617800 0.33246600 0.96602900  O 2.05520000 1.18685700 0.48387400  H 2.08124700 1.83560300 1.20482100  O 3.48683000 -1.11467200 -0.57503500  H 3.23890300 -0.30814000 -0.09365600  H 4.29778400 -0.90242600 -1.05138400 |
| CP-TS1-1WTB3 | C -1.16075800 1.43058400 0.09890400  C -0.03159800 0.82801200 0.49503600  C -0.10605000 -0.67818900 0.52288700  C -1.50158700 -1.04455300 0.15207900  S -2.48864000 0.30577200 -0.26839700  H -1.33312000 2.49221900 -0.02752600  H 0.88774000 1.35499200 0.72789600  H 0.21178400 -1.10849000 1.48404300  H -1.88333300 -2.05421000 0.09535600  O 0.76627400 -1.29160700 -0.49050400  H 2.40894500 -2.15740600 -0.07152200  O 2.14020500 -1.23167900 0.03890200  O 3.31136500 1.41546400 -0.09183500  H 3.04290900 0.48885000 -0.20905200  H 3.54052400 1.73914800 -0.97079000 |
| CP-TS2-1WHA | C 0.23949000 -0.63079200 0.58241200  C 0.32762800 0.78019700 1.08333500  C 1.33834300 1.52819700 0.51297800  C 2.14462100 0.82286300 -0.38704900  S 1.67039700 -0.85277200 -0.56678400  H 0.26885100 -1.39215200 1.36889200  H -0.39255500 1.14682200 1.80465000  H 1.50689600 2.57734900 0.73417600  H 2.98844400 1.20439600 -0.94658100  O -2.04393700 -1.04292600 0.72070300  H -2.60779100 -0.30055100 0.40817800  O -0.93136100 -0.89952900 -0.22328900  O -2.91278300 1.12288600 -0.79154100  H -3.60287900 1.07934000 -1.46494200  H -2.08316400 0.88291100 -1.23287200 |
| CP-TS2-1WHB | C 0.06698500 0.29527300 0.48447700  C 1.22128900 1.24700100 0.42772800  C 2.39267900 0.68889100 -0.04616800  C 2.31151100 -0.68126700 -0.31315200  S 0.74043600 -1.36192400 0.04397900  H -0.45501700 0.25468300 1.44538800  H 1.09602200 2.28194200 0.72216100  H 3.30879100 1.25080900 -0.19671400  H 3.09896100 -1.32168400 -0.68800600  O -1.66773300 1.75401200 -0.11917100  H -1.41930600 2.36125800 -0.83610600  O -0.94478300 0.54693900 -0.53867500  O -3.25878600 -1.19170900 0.08992300  H -2.53952300 -0.66221300 -0.29088000  H -3.92127000 -0.54733400 0.36657000 |
| CP-TS2-1WHC | C 0.22820600 0.14685300 0.51101700  C 1.13815000 1.32371200 0.33314500  C 2.38644400 1.00684400 -0.16575300  C 2.60607400 -0.36229600 -0.34570800  S 1.23749600 -1.34633000 0.12355800  H -0.21754700 0.06007800 1.50726800  H 0.79435500 2.32390000 0.56804800  H 3.14662000 1.74565500 -0.39804500  H 3.50210500 -0.83712600 -0.72286600  O -1.83087400 1.10707500 -0.03270600  H -1.79918600 1.71055000 -0.79416600  O -0.85898700 0.08862300 -0.45303900  O -4.29416900 -0.54074300 0.00919800  H -3.48550800 -0.00408100 0.04182000  H -4.02177200 -1.38801200 -0.36281600 |
| CP-TS2-1WTD | C -0.47310500 -0.27423000 0.09636900  C -0.57535000 0.76003000 1.17772900  C 0.20480800 1.88200300 0.97903800  C 1.00757700 1.83550400 -0.16416300  S 0.82244200 0.34585400 -1.07119500  H -0.20498300 -1.27497300 0.44988200  H -1.23411500 0.60082600 2.02302700  H 0.21050400 2.73642000 1.64831100  H 1.68799600 2.59828500 -0.51870500  O -2.61895400 -1.16354800 0.07009400  H -3.32797800 -0.50641100 0.16562900  O -1.65870500 -0.39274800 -0.72434400  O 2.22484600 -2.24944900 0.69989000  H 2.33031000 -1.51504300 0.07669000  H 2.81812000 -2.94666200 0.39531900 |
| CP3 | C 0.68816800 -0.12577300 1.27322900  C 1.73664200 0.52393800 0.73549400  C 1.73592300 0.53029700 -0.73192100  C 0.68690400 -0.11463600 -1.27435500  S -0.23733800 -0.97433000 -0.00380800  H 0.43350400 -0.29048900 2.31103300  H 2.51513700 1.00350200 1.31966800  H 2.51395000 1.01476100 -1.31267200  H 0.43158500 -0.27046500 -2.31336900  O -1.73702000 -0.68258000 -0.00146800  O -1.81763800 1.66737100 0.00586800  H -2.74532700 1.37069700 0.00638200 |
| CP-TS3-1WHA | C -0.35216800 -0.63737300 1.09898200  C -0.71016500 -1.65538400 0.28999600  C -1.36764300 -1.21844900 -0.94743700  C -1.49096400 0.11715900 -1.05244800  S -0.98612600 0.92381600 0.47273800  H 0.13653900 -0.66778900 2.06253700  H -0.52871100 -2.69813800 0.52128500  H -1.71197000 -1.91955700 -1.70051200  H -1.93040500 0.71016600 -1.84320200  O 0.11072900 1.97451700 0.25886200  O 2.01239900 -1.77246100 0.23586800  H 2.22553700 -0.87748600 -0.14297700  O 2.41046100 0.83944800 -0.73085600  H 1.64148900 1.36681300 -0.41003400  H 3.20246700 1.33720100 -0.49645800 |
| CP-TS3-1WHB | C -0.22608900 1.09749100 -0.45578900  C -1.27923500 1.60151500 0.21201600  C -2.26412400 0.59216100 0.62048900  C -1.93250900 -0.66518000 0.27126800  S -0.50410500 -0.63523300 -0.80831200  H 0.68051700 1.56119300 -0.82579300  H -1.39185100 2.65442300 0.44754700  H -3.17481900 0.84904600 1.15187200  H -2.47589300 -1.58905100 0.41467600  O 0.64861500 -1.55447000 -0.37779600  O 1.58460100 -0.67471700 1.47473100  H 1.85329900 -1.60648300 1.55374700  O 2.84255600 1.14075000 -0.26957300  H 3.64288200 0.79232800 -0.67820100  H 2.53712100 0.45385400 0.36233500 |
| CP-TS3-1WTB | C -0.72810400 -1.21402900 0.97818300  C 0.61313500 -1.11512500 1.02359100  C 1.19656200 -0.38945900 -0.11236900  C 0.27727800 0.04164000 -0.99598300  S -1.33809000 -0.61889200 -0.59730700  H -1.41207800 -1.69123600 1.66637400  H 1.22235300 -1.52791200 1.82113000  H 2.26436300 -0.21727200 -0.21032000  H 0.40869300 0.59505200 -1.91552800  O -2.44361200 0.43372000 -0.49255800  O -1.52015800 2.09647400 0.90129300  H -2.43710000 2.35515700 0.69991900  O 4.39676500 0.55449100 -0.10583200  H 4.50252000 1.45987400 0.21309000  H 5.24350300 0.31297200 -0.50151800 |
| CP-TS4-1WHA | C -2.15819100 0.92922900 -0.15509100  C -0.94063800 1.33116800 0.23251300  C 0.02663700 0.21418600 0.53175300  C -0.73677100 -1.05732100 0.37732700  S -2.35509100 -0.83746600 -0.18311900  H -2.99603000 1.54779500 -0.45211100  H -0.63282900 2.36833600 0.29263800  H 0.50042100 0.31084900 1.51973800  H -0.34777900 -2.04666900 0.57388700  O 1.12734500 0.18634100 -0.43810100  H 2.82880400 0.82118200 0.08590700  O 2.00620800 1.31583000 -0.11677300  O 3.63001300 -0.96383900 0.04617500  H 4.30459900 -1.23921100 -0.58623700  H 2.76952000 -1.17305800 -0.35334500 |
| CP-TS4-1WTA | C -0.38338300 0.75784800 0.44097100  C -0.78054400 -0.21090000 1.33456000  C -1.97807000 -0.88309000 0.93198100  C -2.47261900 -0.40671000 -0.25427100  S -1.48420500 0.86492300 -0.89816200  H 0.46642000 1.42787600 0.51065300  H -0.24604200 -0.41950100 2.25511600  H -2.44588700 -1.68065400 1.49748600  H -3.35312200 -0.73360400 -0.79054100  O 1.53375700 -1.69575800 -0.47775400  H 0.92056000 -1.01269300 -0.09680600  O 2.73797200 -1.13755000 -0.53168700  O 2.68717900 1.60903100 0.46493800  H 3.51513300 2.09940800 0.52632800  H 2.90664400 0.73172700 0.10493800 |
| CP5 | C -0.17348200 -0.55969100 0.50349000  C -0.26084000 0.91957900 0.50729100  C 1.00315800 1.51413300 0.01817200  C 1.93102900 0.59696800 -0.31003600  S 1.47339400 -1.09106700 -0.02365000  H -0.69830200 -1.22162400 1.18638800  H -0.92574300 1.46015400 1.17538500  H 1.15299300 2.58379700 -0.06961400  H 2.90576600 0.79514200 -0.74055300  O -0.97211100 0.09307300 -0.48496400  O -3.79858100 -0.20401400 -0.15264500  H -2.84267600 -0.09880000 -0.38583500 |
| CP-TS5-1WHA | C -1.56342300 0.79191500 0.09795300  C -0.77344300 0.53724600 1.32607600  C -0.05808200 -0.75532300 1.22411200  C -0.28257900 -1.40200200 0.06126900  S -1.41708900 -0.60699200 -1.04241300  H -2.49082600 1.35515900 0.05047300  H -1.03631700 0.96748400 2.28808200  H 0.58788600 -1.13823100 2.00366800  H 0.18393000 -2.32680500 -0.25324400  O -0.38156100 1.55642800 0.33230300  O 2.51466300 -1.28569100 0.22280800  H 2.56697000 -0.36505100 -0.14046200  O 2.27297500 1.36636000 -0.72847900  H 1.34681800 1.53019100 -0.46803700  H 2.33150000 1.56134100 -1.67138800 |
| CP-TS5-1WHA2 | C 1.00679100 -0.53483300 -1.07871700  C 0.06247800 -1.20039000 -0.14771500  C 0.43360600 -0.75683800 1.24643000  C 1.38670700 0.17960000 1.28829200  S 2.00773400 0.63330300 -0.33249900  H 1.04998700 -0.72596500 -2.14153800  H -0.09728900 -2.27845500 -0.26374200  H -0.08213300 -1.13878100 2.11917200  H 1.77730800 0.70647200 2.14972200  O -0.96638000 -0.44269000 -0.74377400  O -2.51317600 -1.04710700 0.12530700  H -2.81460900 -0.13804500 0.32472300  O -2.17074900 1.93354000 0.13627400  H -2.55714000 2.44681300 -0.58323400  H -1.53491200 1.31992700 -0.27731000 |
| CP-TS5-1WHB | C 0.41467700 -0.53961300 0.61900400  C -0.03930200 0.71322000 -0.02703900  C 1.10682700 1.41135400 -0.65085300  C 2.27979400 0.77438000 -0.48408500  S 2.21091600 -0.70892600 0.48538500  H -0.04817100 -0.99568700 1.48915400  H -0.94185800 1.22845400 0.29068900  H 0.99894900 2.34621600 -1.18835700  H 3.23798400 1.07194400 -0.89410600  O -0.31404400 -0.62701300 -0.60821900  O -3.00357800 -1.32590800 -0.75737000  H -2.01859900 -1.17294900 -0.77497100  O -3.15805200 1.07710900 0.76734600  H -3.38182100 0.27688200 0.25536600  H -3.98772800 1.43841200 1.09985500 |
| CP-TS5-1WTA | C 1.07527000 0.65595900 0.48304000  C 1.00211500 -0.80574000 0.71301800  C -0.38504600 -1.28853700 0.53659900  C -1.25836400 -0.31585800 0.21596700  S -0.57235400 1.31906600 0.14687400  H 1.78257500 1.33106400 0.95642000  H 1.71454500 -1.33005900 1.34430100  H -0.66126000 -2.33050400 0.64938400  H -2.31277900 -0.43198800 -0.01409900  O 1.61332700 -0.23084500 -0.50209100  O 4.45221800 -0.24222100 -0.74044700  H 3.46214000 -0.26086400 -0.75839300  O -4.48727600 -0.39831400 -0.59102900  H -5.26014700 -0.20549400 -0.04560800  H -4.79741300 -0.38111800 -1.50519200 |
| CP-TS5-1WTB | C 0.41467700 -0.53961300 0.61900400  C -0.03930200 0.71322000 -0.02703900  C 1.10682700 1.41135400 -0.65085300  C 2.27979400 0.77438000 -0.48408500  S 2.21091600 -0.70892600 0.48538500  H -0.04817100 -0.99568700 1.48915400  H -0.94185800 1.22845400 0.29068900  H 0.99894900 2.34621600 -1.18835700  H 3.23798400 1.07194400 -0.89410600  O -0.31404400 -0.62701300 -0.60821900  O -3.00357800 -1.32590800 -0.75737000  H -2.01859900 -1.17294900 -0.77497100  O -3.15805200 1.07710900 0.76734600  H -3.38182100 0.27688200 0.25536600  H -3.98772800 1.43841200 1.09985500 |
| CP-TS5-1WTB2 | C 1.37074300 -0.91204300 0.57028200  C -0.01921000 -0.40624800 0.71050200  C -0.01738600 1.02944900 0.23798100  C 1.14793800 1.42964100 -0.28027500  S 2.41588800 0.15912600 -0.24263500  H 1.69911500 -1.88684800 0.90158000  H -0.52222200 -0.57385500 1.67130800  H -0.91661400 1.63585700 0.28310000  H 1.39275400 2.37910100 -0.73937100  O -0.39262200 -1.34885600 -0.26961500  O -2.22777700 -1.30546300 -0.38910800  H -2.19447800 -1.59578700 -1.31503600  O -3.13945200 1.28411400 0.07579400  H -4.03066100 1.25863300 0.44172800  H -2.89580400 0.35372500 -0.12865200 |
| CP8a | C -1.00367000 0.49425300 -0.01742500  C 0.03147300 1.54510000 -0.04574000  C 1.36001600 1.38583000 0.17368600  C 2.11104900 0.15028400 0.36298500  S 1.77980100 -1.33754300 -0.23537800  H -0.76222400 -0.47272900 0.44295000  H -0.35656800 2.54819400 -0.20841100  H 1.96292000 2.29143800 0.24428200  H 3.02763700 0.28266900 0.94530600  O -3.30441000 -1.18340000 0.43232000  H -3.89189400 -0.91102000 -0.29715800  O -2.12682700 0.70956700 -0.45756500 |
| CP-TS8a-1WHA1 | C -0.37898000 1.02573200 0.03829900  C 0.90010900 1.72314800 0.25048900  C 2.11278900 1.14465400 0.43119300  C 2.46517500 -0.26684700 0.32441900  S 1.77400400 -1.40330500 -0.62809100  H -0.44754400 -0.04101200 0.29773800  H 0.81690800 2.80595600 0.31273100  H 2.94108900 1.79709200 0.70851300  H 3.32647200 -0.55167700 0.93575700  O -2.03818300 -1.88454600 1.02998700  H -2.70898000 -1.37070600 0.50341700  O -1.36921400 1.63536400 -0.36451600  O -3.54209800 -0.10728500 -0.44228400  H -2.89959400 0.63265500 -0.49214100  H -4.41101300 0.27217600 -0.26844300 |
| CP-TS8a-1WHA2 | C -0.38020900 -1.02599600 -0.03844600  C 0.89881600 -1.72413500 -0.24854200  C 2.11191900 -1.14646800 -0.42893000  C 2.46519800 0.26499900 -0.32412200  S 1.77436500 1.40356500 0.62603600  H -0.44800700 0.04059900 -0.29856500  H 0.81519200 -2.80699500 -0.30936400  H 2.94011200 -1.79977700 -0.70447700  H 3.32710800 0.54811900 -0.93538800  O -2.03256500 1.88602600 -1.02551400  H -2.70529300 1.37393400 -0.49970400  O -1.37118100 -1.63490700 0.36368400  O -3.54379600 0.10867400 0.43966100  H -4.41151000 -0.27026800 0.25879200  H -2.90145200 -0.63140000 0.48971100 |
| CP-TS8a-1WHB | C 0.52061900 -0.93054200 0.02268900  C -0.68830800 -1.74831800 0.23312700  C -1.94178200 -1.27538100 0.43770400  C -2.39339500 0.10826700 0.35491800  S -1.78736500 1.29297300 -0.59632400  H 0.52365100 0.11553500 0.36108400  H -0.51218500 -2.82092600 0.26875300  H -2.71313000 -1.99459000 0.71383400  H -3.25795300 0.33114900 0.98710000  O 3.44640300 -0.10351900 -0.14416800  H 3.48235900 -0.38796600 -1.07537100  O 1.53114700 -1.43140500 -0.46793100  O 1.71916800 1.94863700 0.64625100  H 2.44540200 1.36317600 0.34763000  H 2.07315200 2.47219400 1.37430200 |
| CP-TS8a-1WHC | C 0.52028400 -0.92981200 0.02244800  C -0.68838200 -1.74792900 0.23319100  C -1.94196800 -1.27539300 0.43800800  C -2.39415000 0.10805700 0.35508600  S -1.78860100 1.29295300 -0.59623700  H 0.52302100 0.11632400 0.36067600  H -0.51188900 -2.82047200 0.26893000  H -2.71298200 -1.99483200 0.71447000  H -3.25875900 0.33064400 0.98730300  O 3.44758600 -0.10432400 -0.14447700  H 3.48294300 -0.38957100 -1.07547500  O 1.53089600 -1.43045100 -0.46822300  O 1.72127200 1.94830200 0.64635100  H 2.44678000 1.36215800 0.34735100  H 2.07576000 2.47075000 1.37493400 |
| CP-TS8a-1WHC2 | C 0.51986000 -0.92970500 0.02300900  C -0.68891500 -1.74784900 0.23321900  C -1.94254400 -1.27528100 0.43757500  C -2.39458600 0.10825400 0.35481600  S -1.78885700 1.29326400 -0.59622800  H 0.52260700 0.11635000 0.36148100  H -0.51244200 -2.82039800 0.26882300  H -2.71380300 -1.99467400 0.71347900  H -3.25930100 0.33075400 0.98692600  O 3.44926700 -0.10432900 -0.14576100  H 3.48166300 -0.38922200 -1.07698000  O 1.53059000 -1.43021200 -0.46752700  O 1.72209800 1.94713600 0.64715800  H 2.07656100 2.47022800 1.37529700  H 2.44790200 1.36146500 0.34795500 |
| CP-TS8a-1WTA | C -0.53309600 0.90630200 0.02514700  C 0.67282100 1.71039100 0.29375900  C 1.92743300 1.23301000 0.48067800  C 2.40432800 -0.13713900 0.33127400  S 1.84644500 -1.28649100 -0.69072600  H -0.51620900 -0.17056200 0.23734800  H 0.49245400 2.77765800 0.39966900  H 2.68827300 1.94319500 0.80550800  H 3.26646800 -0.37274900 0.96204700  O -3.27622800 -0.11510100 -0.71224300  H -3.73441200 0.70576900 -0.45666900  O -1.56442000 1.45077700 -0.36438700  O -1.91057200 -1.67630200 1.16831500  H -2.53971600 -1.22745900 0.56696500  H -2.01913000 -2.62238400 1.01811700 |
| CP-TS8a-1WTA2 | C 1.50681000 0.61856500 0.09217100  C 0.20684100 1.25769700 0.35943300  C -1.02025700 0.69951600 0.20949300  C -1.35936900 -0.67930500 -0.12249900  S -0.51670400 -2.04245600 0.22381000  H 1.52678200 -0.31430100 -0.48630800  H 0.27563600 2.30354400 0.65106100  H -1.89205600 1.34439500 0.31457400  H -2.33116900 -0.76938300 -0.61356100  O 4.16203300 -0.11492200 -0.79025500  H 4.69009200 0.22657300 -0.04493000  O 2.55323500 1.13729700 0.46725900  O -4.25943500 1.01105100 -0.38793900  H -4.99065500 0.80043000 0.20683500  H -4.66217000 1.44179100 -1.15273600 |
| CP-TS8a-1WTB | C 1.25772900 -0.63683300 0.03851400  C -0.14917000 -1.03275100 -0.12080400  C -1.24349300 -0.25016100 0.05643500  C -1.29763800 1.18413100 0.31542900  S -0.25092400 2.34837200 -0.16933900  H 1.47458900 0.31966600 0.53748600  H -0.29129500 -2.08658300 -0.35039800  H -2.21956800 -0.73625100 0.02930300  H -2.19480500 1.49074800 0.86073500  O 4.84868500 -0.56055300 0.26700300  H 3.95287700 -0.89152700 0.00418100  O 2.17147000 -1.36815400 -0.33343400  O -4.32489100 -1.52752100 0.04973400  H -4.91593900 -1.55756100 -0.71338400  H -4.75774800 -2.04893900 0.73762500 |
| P1 | C 0.55448700 -1.11927300 0.33409500  C -0.60426600 -0.20054400 0.53201900  C -0.08138000 1.19571300 0.30381700  C 1.19874700 1.25842000 -0.08943800  S 1.99096800 -0.32627500 -0.20697100  H 0.50992300 -2.19609400 0.41575700  H -1.08135600 -0.32230800 1.51589800  H -0.71475100 2.06553300 0.43481000  H 1.77245100 2.14381300 -0.33461600  O -1.61441200 -0.59341600 -0.45018300  O -2.85179600 0.10951600 -0.08639300  H -3.01762300 0.59476600 -0.91065900 |
| P2 | C -0.41867700 0.39767000 0.48876000  C 0.65786900 1.41672900 0.27182600  C 1.85170400 0.90270100 -0.19316100  C 1.88108400 -0.48956100 -0.32412600  S 0.39492800 -1.26497900 0.16784000  H -0.84704000 0.37075600 1.49633400  H 0.44539400 2.46723600 0.42892500  H 2.71078400 1.52017100 -0.43519700  H 2.71212600 -1.08825400 -0.67346900  O -2.60649400 -0.14625400 -0.07974100  H -2.63733900 -0.73474400 -0.85314000  O -1.46033700 0.68866000 -0.43384500 |
| P3 | C -0.39358100 1.27150400 0.00242700  C -1.62324800 0.73278600 0.12678900  C -1.62328900 -0.73270400 0.12678500  C -0.39365300 -1.27152000 0.00249400  S 0.81716000 -0.00003900 -0.37186400  H -0.10202400 2.31219500 -0.04742300  H -2.53612400 1.31486100 0.20508900  H -2.53620400 -1.31472900 0.20503000  H -0.10222800 -2.31224400 -0.04743300  O 2.05058000 0.00001800 0.51044900 |
| P4 | C -1.55930000 -0.44366600 0.19848300  C -1.10853300 0.93236300 -0.10562800  C 0.15598800 1.42014100 -0.05621200  C 1.40852900 0.71577300 0.19195100  S 1.78022100 -0.85244700 -0.10365300  H -0.84391600 -1.11858100 0.69515100  H -1.92180400 1.61669200 -0.33871600  H 0.27426400 2.49518600 -0.19437000  H 2.19691200 1.36446200 0.58506700  O -2.69613600 -0.80828400 -0.05753100 |
| P5 | C 0.47207600 -0.95036900 -0.33813100  C 1.26221600 0.30417600 -0.37528200  C 0.39658700 1.46952300 -0.07890500  C -0.88709600 1.13990100 0.15205600  S -1.27764600 -0.57992600 -0.02175400  H 0.66519300 -1.83915500 -0.93281400  H 2.16515100 0.40738900 -0.97172700  H 0.77288000 2.48541400 -0.04178000  H -1.68042700 1.81666700 0.44785700  O 1.38210700 -0.67136200 0.71101300 |
